# Spinal Cord Tau and Protein Copathologies Associated With Chronic Traumatic Encephalopathy

**DOI:** 10.1001/jamaneurol.2025.5421

**Published:** 2026-01-26

**Authors:** Hidetomo Tanaka, Lauren E. Black, Shelley L. Forrest, Krisztina Danics, Nusrat Sadia, Mozhgan Khodadadi, Charles Tator, Douglas H. Smith, Maria Carmela Tartaglia, William Stewart, Gabor G. Kovacs

**Affiliations:** 1 Tanz Centre for Research in Neurodegenerative Disease, University of Toronto, Toronto, Ontario, Canada; 2 School of Psychology and Neuroscience, University of Glasgow, Glasgow, United Kingdom; 3 Krembil Brain Institute, University Health Network, Toronto, Ontario, Canada; 4 Department of Pathology, Forensic and Insurance Medicine, Semmelweis University, Budapest, Hungary; 5 Canadian Concussion Centre, Krembil Brain Institute, University Health Network, Toronto, Ontario, Canada; 6 Department of Neurosurgery, Penn Center for Brain Injury and Repair, Perelman School of Medicine, University of Pennsylvania, Philadelphia; 7 University Health Network Memory Clinic, Toronto Western Hospital, Toronto, Ontario, Canada; 8 Division of Neurology, Department of Medicine, University of Toronto, Toronto, Ontario, Canada; 9 Department of Neuropathology, Queen Elizabeth University Hospital, Glasgow, United Kingdom; 10 Laboratory Medicine Program, University Health Network, Toronto, Ontario, Canada

## Abstract

**Importance:**

Exposure to repetitive head impacts (RHI) is associated with increased risk of a range of neurodegenerative diseases, including Alzheimer disease and amyotrophic lateral sclerosis. However, while the protein pathologies in the brains of individuals with the RHI-associated pathology of chronic traumatic encephalopathy (CTE) are well described, the spinal cord pathology in at-risk individuals remains poorly understood.

**Objective:**

To evaluate spinal cord pathologies associated with RHI exposure or CTE neuropathologic change (CTE-NC) in the brain.

**Design, Setting, and Participants:**

This case-control study of a retrospective autopsy series (June 2019 to August 2025) was performed among autopsied individuals who served as RHI-exposed cases or controls in a multicenter brain bank collaboration. Data analysis was performed from January 2024 to November 2025.

**Exposures:**

RHI history and CTE-NC presence.

**Main Outcomes and Measures:**

Informant-reported clinical history as well as symptoms and immunohistochemistry for phosphorylated tau (p-tau), phosphorylated TAR DNA-binding protein 43 (p-TDP-43), α-synuclein, and amyloid-β (Aβ), as well as amyloid precursor protein and human leukocyte antigen DR.

**Results:**

Of 70 autopsied individuals (62 male, 8 female; mean [SD] age, 64.40 [13.94] years), 20 showed CTE-NC in the brain. All cases with CTE-NC exhibited spinal cord p-tau deposits, especially in cases aged 65 years or older with prior RHI (n = 14), often showing extensive spinal tau pathology as both neuronal (all 14 cases) and astrocytic (12 of 14 cases [86%]) p-tau deposits. Spinal p-tau pathology was associated with microglial activation and motor symptoms. Notably, among the individuals with CTE-NC and prior RHI who were aged 65 years or older, additional spinal protein pathologies were present, comprising p-TDP-43 inclusions (9 of 14 cases [64%]), Aβ deposits (13 of 14 cases [93%]), and α-synuclein deposits (7 of 14 cases [50%]), with all 4 of these pathologies present in 4 individuals (29%). In total, across all 20 CTE-NC cases, p-TDP-43 inclusions were confined to the spinal cord in 5 of the 10 individuals with spinal p-TDP-43 pathology. In contrast, among 50 individuals without CTE-NC, typically sparse p-tau deposits were seen in only 27 (54%). Among the 23 confirmed cases with a history of RHI, 16 (70%) exhibited CTE-NC, while 7 (30%) did not. Spinal tau pathology was more severe in those with CTE-NC; however, astrocytic tau pathology was also present in the group without CTE-NC, unlike in controls without RHI or CTE.

**Conclusions and Relevance:**

This case-control study provides autopsy evidence of a high prevalence of complex spinal pathology in individuals with CTE-NC, supporting the concept of trauma-related encephalomyelopathy. The frequent co-occurrence of p-TDP-43, Aβ, and α-synuclein pathologies in individuals aged 65 years or older with CTE-NC suggests that cumulative trauma might contribute to widespread misfolded protein aggregation.

## Key Points

**Question:**

Are phosphorylated tau (p-tau) and other misfolded protein pathologies present in the spinal cord of individuals with repetitive head impact (RHI) exposure or chronic traumatic encephalopathy neuropathologic change (CTE-NC)?

**Findings:**

In this case-control study, p-tau pathology in the spinal cord was observed in all CTE-NC cases with RHI, and older cases frequently exhibited phosphorylated TAR DNA-binding protein 43 (64%), amyloid-β (93%), and α-synuclein (50%) pathology; all these pathologies were more prevalent than in controls. Severity of p-tau pathology was associated with clinical symptoms and microglial activation in the spinal cord.

**Meaning:**

These findings suggest that the concept of CTE-NC may need to be expanded to encompass chronic trauma–induced encephalomyelopathy.

## Teaser

This case-control study evaluates spinal cord pathologies associated with exposure to repetitive head impacts or chronic traumatic encephalopathy neuropathologic change in the brain among autopsied individuals.

## Introduction

Epidemiological data demonstrate high risk of various neurodegenerative diseases, including Alzheimer disease and amyotrophic lateral sclerosis (ALS), among former elite-level contact sport athletes.^1,2,3^ In parallel, autopsy studies of former contact sport athletes show high prevalence of chronic traumatic encephalopathy (CTE) neuropathologic change (CTE-NC), a progressive tauopathy characterized by abnormal accumulation of misfolded tau protein in the brain, appearing as perivascular tau aggregates in neurons at the depths of cortical sulci, with or without astrocytic involvement.^4,5,6,7^ Indeed, to date, virtually all reported cases of CTE-NC report prior exposure to mild traumatic brain injury (TBI) and/or repetitive head impacts (RHI).^5^ However, in addition to the well-characterized phosphorylated tau (p-tau) pathology of CTE-NC, a number of other neurodegenerative proteinopathies are recognized in individuals with RHI exposure,^8,9,10^ the clinical relevance of which remains uncertain. Nevertheless, while there has been considerable focus on the neuropathology of CTE-NC in the brain, less attention has been paid to spinal pathologies in individuals exposed to RHI despite the known structural susceptibility of the spine to trauma.^11,12^

There is a paucity of data on late neurodegenerative pathologies in the spinal cord in individuals with past mild TBI or RHI exposure, with limited previous work largely focused on contact sport athletes. To address these gaps, we performed a comprehensive neuropathologic analysis of spinal cords from 70 individuals, including those with and without RHI and autopsy-proven CTE-NC. To our knowledge, this multicentric study represents the first systematic characterization of various spinal protein pathologies and highlights the significance of spinal involvement in the broader pathological landscape of CTE.

## Methods

### Study Design and Participants

To assess spinal cord pathology in association with RHI exposure and CTE-NC status, cases were grouped by both factors. Subgroups included participants who had CTE with RHI, those who did not have CTE but did have RHI, and those who did not have CTE or RHI (controls). This design enabled evaluation of whether pathology correlated more strongly with RHI exposure alone or with the presence of CTE-NC. Considering prior evidence that age influences CTE-NC severity in the brain,^13^ age-stratified analyses were also performed (eFigures 1 and 2 in Supplement 1).

Cases were selected from 3 neuropathology centers: consecutive cases (June 2019 to August 2025) referred through the Canadian Concussion Centre to the University Health Network neurodegenerative disease brain collection in Toronto, Ontario, Canada; cases randomly selected from the Glasgow Traumatic Brain Injury Archive in the United Kingdom; and cases from a forensic cohort of homeless individuals in Budapest, Hungary.^14^ Cases included had their history of exposure to RHI noted as confirmed RHI (contact sports or military service, n = 23), indeterminate RHI (homeless individuals without professional sports or military service, n = 34), and no RHI (no known history of RHI or TBI, n = 13). Four of the non-RHI cases had spinal stenosis. Further details on participants are available in the eMethods and eTable 1 in Supplement 1.

This study followed the Strengthening the Reporting of Observational Studies in Epidemiology (STROBE) reporting guideline and was approved by the Research Ethics Board of the University Health Network and by corresponding institutional committees at collaborating centers in Glasgow and Budapest. Written informed consent for brain donation and research use was obtained from the next of kin, except for forensic cases in Budapest, which were authorized by the responsible legal authority and institutional regulations, in accordance with institutional and national ethical standards. Data analysis was performed from January 2024 to November 2025.

### Neuropathologic Examination

Spinal cord sections from 3 levels (cervical, thoracic, and lumbar) and brain regions required for staging of CTE-NC and mixed pathologies^7,15,16,17,18,19,20^ were examined using the following stains: hematoxylin and eosin with Luxol fast blue, and immunohistochemistry for p-tau, 3-repeat (3R) tau, 4-repeat (4R) tau, phosphorylated TAR DNA-binding protein 43 (p-TDP-43), α-synuclein, amyloid-β (Aβ), amyloid precursor protein (APP), and human leukocyte antigen DR (HLA-DR; a marker of activated microglia). The severity of spinal cord pathologies was rated using a semiquantitative scale where each marker was assigned a score reflecting its presence and intensity, ranging from absent (0) to severe or extremely severe (up to 4 or 5 depending on the marker). Details of the neuropathologic methods are provided in the eMethods, eFigure 3, and eTable 2 in Supplement 1.

### Statistical Analysis

Statistical analyses were performed using GraphPad Prism version 10.4.1 (Dotmatics). A significance threshold of *P* < .05 (2-tailed) was applied. Fisher exact test was used for categorical variables, and Mann-Whitney *U* test was used for ordinal and continuous variables. For comparisons among multiple groups, the Kruskal-Wallis test was applied, followed by post hoc analysis with Dunn test. Correlations between variables were assessed using 2-tailed nonparametric Spearman rank correlation. Further details are provided in the eMethods in Supplement 1.

## Results

### Overview of RHI Cohort

Among 70 autopsied individuals (62 male, 8 female; mean [SD] age, 64.40 [13.94] years), brain neuropathology revealed typical CTE-NC in 16 of 23 confirmed RHI cases (70%) and 4 of 34 indeterminate RHI cases (12%) but none of 13 non-RHI cases, which served as controls (eTable 1 in Supplement 1). In total, 20 cases with CTE-NC and 50 without were included in this cohort. Notably, the confirmed RHI group, showing high brain CTE-NC frequency, also exhibited frequent abnormal misfolded protein depositions in the spinal cord: p-tau, 20 of 23 cases (87%); p-TDP-43, 10 of 23 cases (43%); Aβ, 17 of 23 cases (74%); and α-synuclein, 7 of 23 cases (30%). In particular, p-TDP-43 was significantly more frequent than in non-RHI cases (43% vs 0 of 13 cases [0%], respectively; *P* = .006). Spinal p-tau pathology severity was also greater (mean [SD] score, 1.333 [1.178] vs 0.742 [0.514], respectively; *P* = .03) (eTable 1 in Supplement 1).

### Spinal Cord Pathology in CTE-NC and Non–CTE-NC Cases

**Figure 1.**
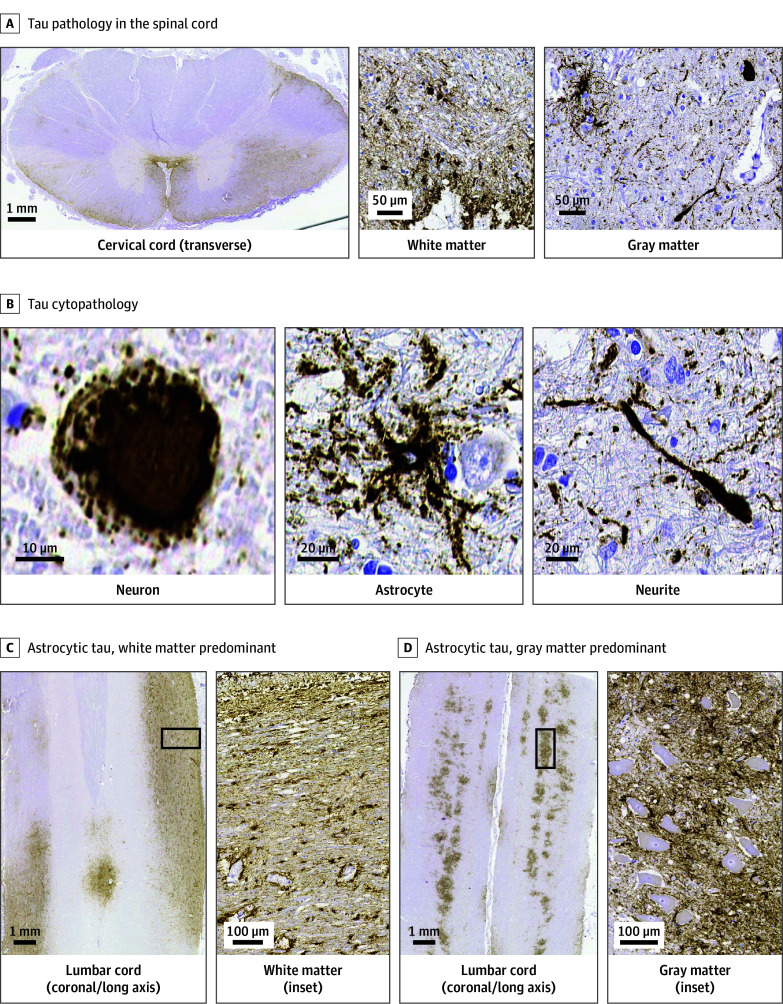
Tau Pathology of the Spinal Cord in Cases With Chronic Traumatic Encephalopathy Neuropathologic Change (CTE-NC) A, All cases with CTE-NC exhibited phosphorylated tau (p-tau) deposition in the spinal cord, involving the white matter (subpial and deeper regions) and the gray matter. The p-tau lesions in the white matter were primarily composed of thorn-shaped astrocytes. B, Tau lesion in the gray matter consisted of p-tau–positive neurons (showing neurofibrillary tangle and pretangle morphology), astrocytes, and spherical neurites with threads. C and D, Tau astrogliopathy-predominant type, in which astrocytic pathology markedly exceeded neuronal p-tau pathology, was observed in 50% of individuals with chronic traumatic encephalopathy (CTE) and repetitive head impacts who were aged 65 years or older. These were further subclassified into white matter–predominant (C) and gray matter–predominant (D) types. C, The white matter–predominant type (representative case: CTE case 8) showed that p-tau–positive astrocytic processes around small vessels were also prominent. D, The gray matter–predominant type (representative case: CTE case 9) showed that many large motor neurons (anterior horn cells) remained p-tau negative despite the abundance of surrounding p-tau–positive astrocytes. All samples shown were immunostained with AT8 (p-tau).

**Figure 2.**
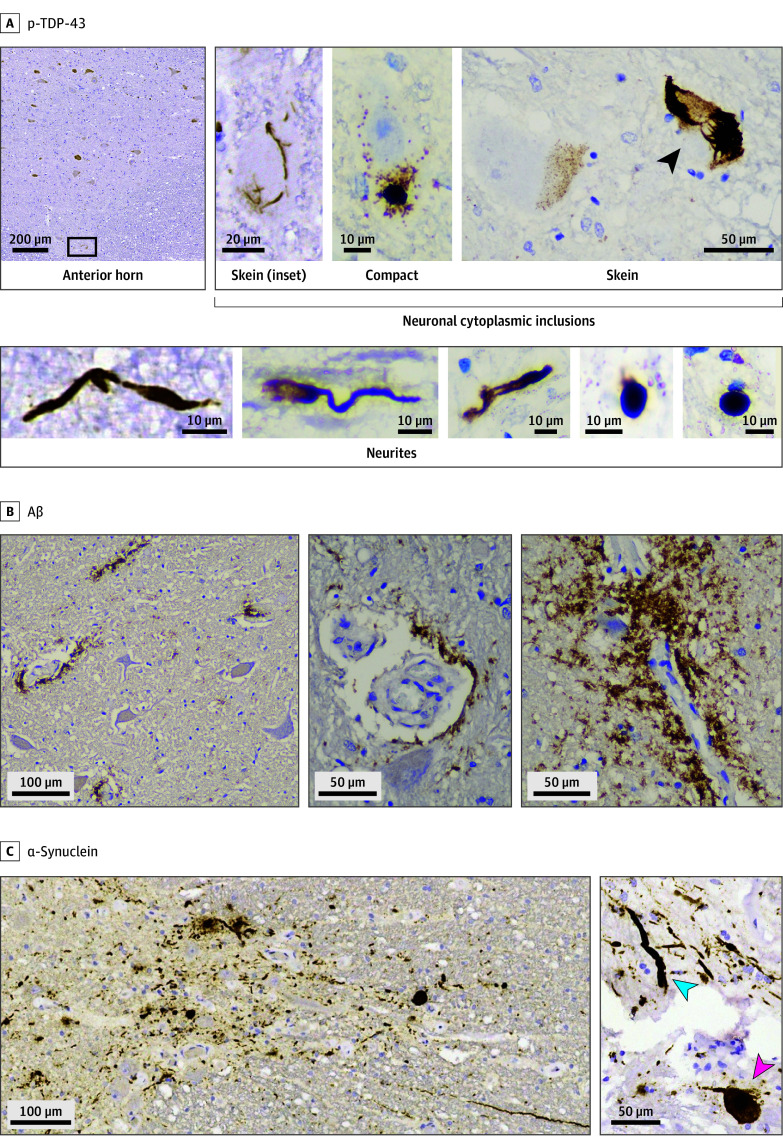
Phosphorylated TAR DNA-Binding Protein 43 (p-TDP-43), Amyloid-β (Aβ), and α-Synuclein Pathology in the Spinal Cord of Cases With Chronic Traumatic Encephalopathy Neuropathologic Change (CTE-NC) A, p-TDP-43–positive inclusions in cases with CTE-NC. In the anterior horn, there were many remaining neurons, and only a few p-TDP-43–positive neuronal cytoplasmic inclusions were observed. These neuronal cytoplasmic inclusions exhibited skeinlike (arrowhead) and compact morphologies, reminiscent of those typically seen in motor neuron disease or amyotrophic lateral sclerosis. Regarding other inclusions, positive neurites with spherical morphology were more frequently found. B, Aβ depositions in the anterior horn. 95% of the CTE-NC cases exhibited Aβ deposition in the spinal cord. The deposition pattern was unique: Aβ was localized predominantly around small vessels, without the presence of typical diffuse or neuritic plaques. Notably, in 2 individuals with chronic traumatic encephalopathy (CTE) with repetitive head impacts who were aged 65 years or older, Aβ was absent in the cerebrum but present in the spinal cord (middle panel, CTE case 3, anterior horn). C, α-Synuclein pathology was found in the lateral horn (5G4 immunostaining). Abundant α-synuclein–positive Lewy bodies (red arrowhead) and long and/or spherical neurites (blue arrowhead) were evident in CTE-NC cases (representative case: CTE case 7).

We next analyzed the RHI cohort by stratifying cases based on the presence or absence of CTE-NC in the brain, forming 2 groups: CTE-NC (n = 20) and non–CTE-NC (n = 50). This comparison further highlighted differences in the spinal cord misfolded protein pathologies. Spinal p-tau pathology was observed in all 20 CTE-NC cases (100%) and was more frequent and severe (Figure 1) compared with non–CTE-NC cases (27 of 50 cases [54%]; *P* < .001 for frequency and severity). Spinal p-tau–positive astrocytic pathology was frequent in CTE-NC cases (15 of 20 cases [75%]), whereas only 3 of 50 non–CTE-NC cases (6%) showed this (*P* < .001). Cases with coincident CTE-NC, compared with non–CTE-NC cases, more frequently had deposits of p-TDP-43 (10 of 20 cases [50%] vs 1 of 50 cases [2%], respectively; *P* < .001) and Aβ (19 of 20 cases [95%] vs 7 of 20 cases [35%], respectively; *P* < .001) in the spinal cord (Figure 2 and Table 1). Spinal p-TDP-43 pathology was notably found in 1 non-CTE case with RHI (eFigure 4 in Supplement 1).

**Table 1.** Summary of Clinicopathological Characteristics in CTE-NC and Non–CTE-NC Cases

Characteristic	Aged ≥65 y at death			All		
	CTE-NC with RHI (n = 14)	Non–CTE-NC, non-RHI controls (n = 11)	*P* value	CTE-NC (n = 20)	Non–CTE-NC (n = 50)	*P* value
Clinical						
Age at death, mean (SD) [range], y	80.50 (5.11) [68-85]	75.82 (4.58) [68-84]	.02	73.20 (12.91) [44-85]	60.88 (12.84) [20-84]	<.001
Sex, No. (%)						
Female	0	2 (18)	.18	0	8 (16)	.09
Male	14 (100)	9 (82)		20 (100)	42 (84)	
Confirmed history of RHI, No. (%)	14 (100)	0	<.001	16 (80)	7 (14)	<.001
Spinal cord						
Total tau						
Frequency, No./total No. (%)^a^	14/14 (100)	10/11 (91)	.44	20/20 (100)	27/50 (54)	<.001
Severity score, mean (SD)^b^	1.917 (1.131)	0.704 (0.542)	<.001	1.574 (1.109)	0.373 (0.541)	<.001
Neuronal tau						
Frequency, No./total No. (%)	14/14 (100)	5/11 (45)	.003	18/20 (90)	15/50 (30)	<.001
Severity score, mean (SD)	1.083 (0.841)	0.370 (0.565)	<.001	0.796 (0.833)	0.176 (0.418)	<.001
Astrocytic tau, No./total No. (%)	12/14 (86)	0/11	<.001	15/20 (75)	3/50 (6)	<.001
In gray matter	10/14 (71)	0/11	<.001	13/20 (65)	3/50 (6)	<.001
In white matter	10/14 (71)	0/11	<.001	12/20 (60)	0/50	<.001
p-TDP-43, No./total No. (%)	9/14 (64)	0/11	.001	10/20 (50)	1/50 (2)	<.001
α-Synuclein, No./total No. (%)	7/14 (50)	2/11 (18)	.21	7/20 (35)	2/20 (10)	.13
Amyloid-β, No./total No. (%)	13/14 (93)	4/11 (36)	.007	19/20 (95)	7/20 (35)	<.001
SAA, No./total No. (%)	2/14 (14)	1/11 (9)	>.99	2/20 (10)	2/20 (10)	>.99
Brain, mixed pathology						
AD-NC						
High-intermediate, No./total No. (%)	11/14 (79)	6/10 (60)	.39	11/20 (55)	10/49 (20)	.008
Severity score, mean (SD)^c^	2.214 (1.122)	2.000 (1.155)	.65	1.700 (1.261)	0.796 (1.118)	.006
Amyloid-β						
Frequency, No./total No. (%)	12/14 (86)	9/10 (90)	>.99	15/20 (75)	22/49 (45)	.03
Severity score, mean (SD)^d^	2.429 (1.089)	2.100 (1.101)	.35	1.850 (1.309)	0.837 (1.124)	.004
CAA, No./total No. (%)	11/14 (79)	7/10 (70)	.67	11/20 (55)	15/49 (31)	.10
AGD, No./total No. (%)	5/14 (36)	0/10	.05	6/20 (30)	6/49 (12)	.09
LBD, No./total No. (%)	8/13 (62)	3/9 (33)	.39	8/19 (42)	10/48 (21)	.12
LATE, No./total No. (%)	8/14 (57)	2/10 (20)	.10	8/20 (40)	5/49 (10)	.007
Other tauopathy, No./total No. (%)^e^	2/14 (14)^f^	0/10	.49	2/20 (10)^f^	1/49 (2)^g^	.20

Abbreviations: AD-NC, Alzheimer disease neuropathologic change; AGD, argyrophilic grain disease; CAA, cerebral amyloid angiopathy; CTE, chronic traumatic encephalopathy; CTE-NC, CTE neuropathologic change; LATE, limbic-predominant age-related TDP-43 encephalopathy; LBD, Lewy body disease; p-TDP-43, phosphorylated TAR DNA-binding protein 43; RHI, repetitive head impacts; SAA, spinal amyloid angiopathy.

^a^ Frequencies were determined as the ratio of positive cases to the number of available cases.

^b^ Severity was determined by averaging scores from all available cases across all spinal cord levels.

^c^ AD-NC severity was determined by averaging the AD-NC scores, which were defined as follows: 0 indicates not AD-NC; 1, low severity; 2, intermediate severity; and 3, high severity.

^d^ Amyloid-β pathology severity was calculated as the average of the A scores of AD-NC.^15^

^e^ Other primary tauopathies included progressive supranuclear palsy, corticobasal degeneration, and globular glial tauopathy.

^f^ Corticobasal degeneration or globular glial tauopathy coexisting with CTE-NC.

^g^ Coexisting progressive supranuclear palsy in a non-CTE case with RHI.

### Comparison of CTE-NC and Non-CTE Cases Within the Confirmed RHI Group

Within the confirmed RHI group (n = 23), we compared cases with and without CTE-NC (CTE-NC with RHI, 16 cases [70%]; non-CTE with RHI, 7 cases [30%]) to determine whether spinal pathology was more strongly correlated with RHI exposure alone or was seen only when CTE-NC was present in the brain. Two non–CTE-NC cases with RHI exhibited not only neuronal but also astrocytic tau pathology in the spinal cord, like CTE-NC cases but unlike controls. The frequency (16 of 16 cases [100%] vs 4 of 7 cases [57%]; *P* = .02) and severity (mean [SD] score, 1.714 [1.175] vs 0.571 [0.746]; *P* < .001) of spinal tau pathology were both higher in the group with both CTE-NC and RHI (eTable 3 in Supplement 1). Therefore, subsequent analyses focused on spinal cord pathology in cases with CTE-NC.

### Details of Spinal Cord Tau Pathology in CTE-NC

All 20 CTE-NC cases exhibited spinal p-tau pathology, ranging from sparse neuronal inclusions and neurites to widespread neuronal and astrocytic tau positivity. Notably, individuals with CTE-RHI aged 65 years or older (n = 14) frequently showed severe spinal tau pathology. Among them, 50% of the cases in which astrocytic tau pathology exceeded neuronal tau pathology were identified as a tau astrogliopathy-predominant type, subclassified into gray matter– and white matter–predominant types (Figure 1; eFigures 5 and 6 and eTable 4 in Supplement 1). Neuronal tau pathology was immunoreactive for 3R tau and 4R tau, while astrocytic tau pathology was mostly 4R tau only, although 3R tau positivity was also detected (eResults and eFigures 7-9 in Supplement 1).

In contrast, controls aged 65 years or older exhibited only mild p-tau deposits, with minimal neuronal and no astrocytic p-tau pathology (total p-tau severity: mean [SD], 1.917 [1.131] vs 0.704 [0.542]; *P* < .001; astrocytic frequency: 12 of 14 cases [86%] vs 0 of 11 cases [0%]; *P* < .001; neuronal frequency: 14 of 14 cases [100%] vs 5 of 11 cases [45%]; *P* = .003; neuronal severity: mean [SD], 1.083 [0.841] vs 0.370 [0.565]; *P* < .001) (Table 1; eTables 5 and 6 in the Supplement). Threadlike p-tau structures in subpial regions were seen in the controls with spinal stenosis,^21^ but distinct p-tau–positive astrocytes were not observed (eFigure 10 in Supplement 1).

In addition, we focused on individuals with CTE-RHI who were aged 65 years or older, in whom spinal cord tau pathology was frequent and often severe, and we compared tau pathology across spinal levels. While total p-tau burden did not significantly differ among levels, neuronal p-tau was more abundant in the cervical cord, and gray matter astrocytic p-tau tended to be higher in the lumbar cord (eResults and eFigure 11 in Supplement 1).

### Spinal Cord TDP-43 Pathology in CTE-NC

Spinal p-TDP-43 pathology was detected in 10 of 20 CTE-NC cases (50%), more frequently in individuals with CTE-RHI who were aged 65 years or older (9 of 14 cases [64%]) (Table 1, Table 2, and Figure 2A). Five had spinal-only p-TDP-43 inclusions with no brain involvement. In 3 cases, skeinlike and compact neuronal cytoplasmic inclusions were seen in the anterior horn, mimicking ALS (eFigures 12 and 14 in Supplement 1). Hyalinlike inclusions and central chromatolysis with axonal spheroids were also present in the anterior horn in the individuals with CTE-NC who were aged 65 years or older. Despite this, no Bunina bodies or overt neuronal loss were observed (eFigure 13 in Supplement 1).

**Table 2.** Summary of Major Clinicopathological Findings in Individuals With Chronic Traumatic Encephalopathy (CTE) and Repetitive Head Impacts Who Were Aged 65 Years or Older

Case No.^a^	Clinical features			Spinal cord pathology							Brain pathology							
	Age at death, y	Duration, y^b^	Documented concussions, No.	Total tau^c^	Astrocytic tau^d^		TDP-43^e^	α-Synuclein^f^	Aβ^c^	SAA^g^	AD-NC	Aβ A score of AD-NC	CAA^g^	AGD^h^	LBD^h^	LATE^h^	Other	CTE level^i^
					GM	WM												
1	Early 80s	18	Multiple	+++	+	+	+	−	++	−	High	3	2	−	Amygdala predominant	2		High
2	Early 70s	10	16	+	−	+	+	−	+++	−	High	3	2	−	−	−	FTLD-TDP, type A	High
3	Late 70s	12	>20	+	+	−	−	−	+++	−	Not	0	−	2	−	−		Low
4	Late 70s	13	6	+	−	−	+	++	+++	2	Not	0	−	1	4	−		Low
5	Mid-80s	26	Multiple	+++	+	+	−	++	+	−	Intermediate	2	2	−	4	2		High
6	Mid-80s	3	3	+	+	−	+	++	+++	−	High	3	2	2	4	−	GGT (II), NIHIBD	High
7	Mid-80s	13	10	++	+	+	−	+++	++	−	Intermediate	3	1	−	5	2		Low
8	Early 80s	8	5	++++	+	+	++	−	+++	−	High	3	2	−	−	2		High
9	Mid-80s	20	7	++++	+	+	+	−	+++	2	Intermediate	3	2	2	−	−	CBD	Low
10	Mid-80s	19	6	+++	+	+	++	−	+++	−	High	3	2	−	−	1		High
11	Mid-80s	11	3	++++	+	+	+	++	++	−	High	3	1	−	4	3		High
12	Late 70s	28	10	++	−	−	+	+++	+	−	Low	2	−	−	4	−		Low
13	Late 60s	30	10	+++	+	+	−	++	++++	−	High	3	2	1	6	1		NA^j^
14	Late 70s	32	>2	+	−	+	−	−	−	−	High	3	1	−	NA	3		High
Mean (SD) or No. (%)^k^	80.50 (5.11)	17.36 (8.89)		14/14 (100)	12/14 (86)^l^		9/14 (64)	7/14 (50)	13/14 (93)	2/14 (14)	High-intermediate, 11 (79)	12/14 (86)	11/14 (79)	5/14 (36)	8/13 (62)	8/14 (57)		Low, 5; high, 8

Abbreviations: Aβ, amyloid-β; AD-NC, Alzheimer disease neuropathologic change; AGD, argyrophilic grain disease; CAA, cerebral amyloid angiopathy; CBD, corticobasal degeneration; FTLD-TDP, frontotemporal lobar degeneration–TDP-43; GGT (II), globular glial tauopathy, type II; GM, gray matter; LATE, limbic-predominant age-related TDP-43 encephalopathy; LBD, Lewy body disease; NA, not available; NIHIBD, neuronal intranuclear hyaline inclusion body disease; SAA, spinal amyloid angiopathy; TDP-43; TAR DNA-binding protein 43; WM, white matter.

^a^ All individuals described herein were male.

^b^ Duration of the exposure (eg, years of contact sports).

^c^ Severity of total tau pathology and Aβ pathology is indicated as follows: −, absent; +, minimal; ++, mild; +++, moderate; ++++, severe.

^d^ Astrocytic tau pathology is indicated as follows: −, absent; +, present.

^e^ TDP-43 pathology is indicated as follows: −, absent; +, present; ++, present with neuronal cytoplasmic inclusions.

^f^ α-Synuclein pathology is indicated as follows: −, absent; +, minimal or mild; ++, moderate; +++, severe.

^g^ For SAA and CAA, − indicates absence; if present, type is shown.

^h^ AGD, LBD, and LATE are presented as staging scores based on their respective established staging criteria.^17,18,19,20^

^i^ Indicates the level of severity of CTE neuropathologic change.^7^

^j^ The level of CTE neuropathologic change could not be reliably assessed due to coexisting very severe AD-NC.

^k^ Data for continuous variables are shown as mean (SD); for categorical variables, frequencies were determined as the ratio of positive cases to the number of available cases.

^l^ Indicates the overall number with astrocytic tau pathology in the GM and/or WM.

### Spinal Aβ and α-Synuclein Pathologies in CTE-NC

Spinal Aβ deposition was present in 19 of 20 CTE-NC cases, typically in a perivascular pattern without classic plaque formation. Interestingly, 2 individuals with CTE-RHI who were aged 65 years or older lacked Aβ in the cerebrum but had spinal deposition. Spinal α-synuclein was present in 7 of 20 CTE-NC cases (35%), more frequently in individuals with CTE-RHI who were aged 65 years or older (7 of 14 cases [50%]), all of whom exhibited brain Lewy body pathology. Morphologically, spinal deposits included both compact neuronal cytoplasmic inclusions and spherical neurites (Figure 2B and C, Table 1, and Table 2).

### Age and Spinal Cord Pathology in CTE-NC

Given prior reports that the severity of CTE-related brain pathology increases with age,^13^ we stratified CTE-RHI cases by age. The CTE-RHI subgroup aged 65 years or older (n = 14) showed more severe spinal tau pathology (mean [SD] total p-tau score, 1.917 [1.131]) along with more frequent spinal p-TDP-43 (9 cases [64%]), α-synuclein (7 cases [50%]), and Aβ (13 cases [93%]) deposition, with all 4 of these pathologies present in 4 of the 14 individuals (29%), compared with the subgroup younger than 65 years (Table 2; eTable 7 in Supplement 1). Based on these findings, we focused subsequent analyses on this CTE-RHI subgroup, where spinal pathology was most pronounced. We conducted further analysis of clinicopathological correlations, including assessments of spinal axonal damage and microglial activation. Further details on individuals younger than 65 years with CTE-NC, indeterminate RHI, or non-CTE with RHI are provided in the eResults, eFigures 14 and 15, and eTables 7 through 16 in Supplement 1.

### Axonal Spheroids, Microglial Activation, and Small Vessel Pathology

**Figure 3.**
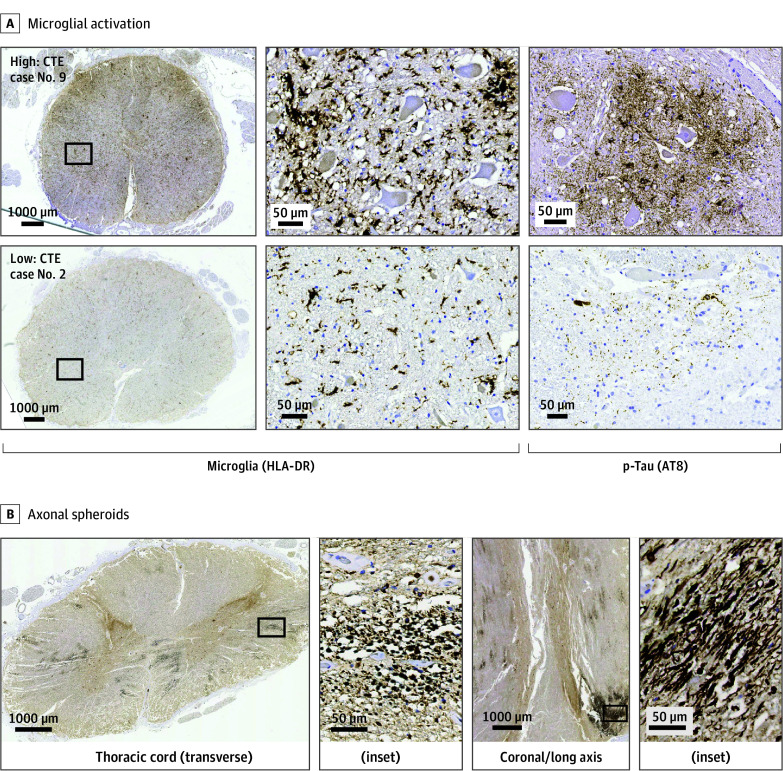
Human Leukocyte Antigen DR (HLA-DR)–Positive Microglial Activation and Amyloid Precursor Protein–Positive Axonal Spheroids in the Spinal Cord of Cases With Chronic Traumatic Encephalopathy Neuropathologic Change A, HLA-DR–positive microglial activation in individuals with chronic traumatic encephalopathy (CTE) and repetitive head impacts who were aged 65 years or older. Top panels are images from a representative case (CTE case 9) showing high HLA-DR–positive microglial activation. The same region exhibited abundant phosphorylated tau (p-tau) pathology (right panel). Bottom panels are images from a representative case (CTE case 2) showing low microglial activation. The p-tau pathology in the same region was minimal (right panel). The middle panels are high-magnification insets of the boxed regions of the left panels. B, Amyloid precursor protein–positive axonal spheroids in a representative individual with CTE and repetitive head impacts who was aged 65 years or older (CTE case 7, thoracic spinal cord). Axonal spheroids were prominent in both transverse (left panels) and coronal and longitudinal (right panels) sections.

APP-positive axonal spheroids, indicative of axonal damage, were found in the spinal cord of all individuals with CTE-RHI (Figure 3B; eFigure 16 in Supplement 1). In individuals aged 65 years or older who had CTE-RHI, HLA-DR–positive microglial activation was observed in the spinal cords and correlated well with the severity of p-tau pathology (Figure 3A; eTable 4 in Supplement 1). In controls, microglial activity was minimal (eFigure 17 in Supplement 1). Small vessel pathology, characterized by fibrotic thickening of vessel walls, was present in the spinal cord in the individuals aged 65 years or older with CTE-RHI and was generally more advanced than in controls (eFigures 18 and 19 in Supplement 1).

### Interactions of Spinal Cord Pathologies

Based on the severity of spinal pathologies in individuals aged 65 years or older with CTE-RHI (eTable 4A in Supplement 1), we analyzed the correlations. As anticipated, total p-tau load and p-tau cytopathology demonstrated a significant positive correlation. Interestingly, HLA-DR–positive microglial activation exhibited a moderate to strong significant positive correlation with total p-tau (*r* = 0.446; *P* = .006) and astrocytic p-tau in the gray matter (*r* = 0.467; *P* = .004) (eFigure 20 in Supplement 1).

### Correlations Between Spinal Cord and Brain Pathologies

Spinal α-synuclein pathology correlated with brain Lewy body disease stage (*r* = 0.886; *P* < .001). However, total p-tau, p-TDP-43, and Aβ pathology loads in the spinal cord did not correlate with their corresponding brain pathologies. The level of CTE-NC in the brain^7^ did not predict the burden of any misfolded protein or the severity of microglial activation and axonal spheroids in the spinal cord (eTable 17 in Supplement 1). Furthermore, because 2 of the present CTE-NC cases had coexisting corticobasal degeneration (CBD) or globular glial tauopathy (GGT) (Table 1), we sought to exclude the potential influence of primary tauopathies other than CTE on spinal cord tau pathology. To address this, CTE-NC cases without coexisting primary tauopathies (eg, argyrophilic grain disease, CBD, or GGT; n = 14) were compared with those with such comorbidities (n = 6), revealing no significant differences. When considering only the non–comorbid CTE group, spinal cord tau pathology remained significantly more prominent compared with controls (eTable 18 in Supplement 1).

### Clinicopathological Correlations

We examined the correlations between clinical factors (eTable 19 in Supplement 1) and both the spinal cord and brain pathology. Spinal microglial activation showed a moderate correlation with age at death (*r* = 0.564; *P* = .04). Spinal cord injury history was strongly correlated with the severity of APP-positive spheroids (*r* = 0.687; *P* = .01) but did not show a significant correlation with misfolded proteins in the brain or spinal cord, including spinal cord p-tau pathology (*r* = 0.245; *P* = .40). Importantly, motor symptoms, encompassing lower motor neuron (LMN) signs and gait disturbance, were significantly correlated with only spinal cord p-tau pathology (total p-tau: *r* = 0.608; *P* = .049; gray matter astrocytic p-tau: *r* = 0.671; *P* = .02) (eTable 20 in Supplement 1). Furthermore, we specifically assessed whether spinal cord tau pathology was associated with LMN-related features, focusing exclusively on LMN signs. LMN signs were documented in 4 individuals with CTE-RHI who were aged 65 years or older (eTable 19 in Supplement 1), but no significant correlation was observed with spinal tau burden (*r* = 0.326; *P* = .22). When the analysis was expanded to include the entire RHI group, the correlation remained nonsignificant, with a higher correlation coefficient (*r* = 0.375) and a lower *P* value (*P* = .09).

## Discussion

In this study, we examined spinal cords from 70 individuals, including 20 with autopsy-confirmed CTE-NC and 50 without. All CTE-NC cases showed more frequent and severe spinal cord p-tau pathology than the non–CTE-NC cases. Specifically, CTE-NC cases showed more extensive total, neuronal, and especially astrocytic p-tau pathology, whereas for non–CTE-NC cases, p-tau pathology was generally mild overall and astrocytic p-tau pathology was rarely noted. From the RHI perspective, spinal tau pathology was more pronounced in CTE cases with RHI than in non-CTE cases with RHI. However, astrocytic tau pathology was still present in non-CTE cases with RHI, distinguishing them from controls (those without both CTE and RHI), where it was absent.

Among main forms of tauopathies, including CBD, progressive supranuclear palsy, and GGT, spinal tau pathology has been reported to involve predominantly neurons.^22,23,24,25,26,27^ Although 2 of the present cases with CTE-NC had coexisting CBD or GGT, their prominent spinal p-tau pathology, especially astrocytic, remained distinct from the findings previously reported.^24,25,26,27^ CTE-NC cases without coexisting primary tauopathies showed no significant differences from those with such comorbidities, yet they still exhibited more pronounced spinal cord tau pathology than controls. Collectively, these observations raise the possibility that the spinal p-tau pathology in CTE-NC reflects processes inherent to CTE and RHI rather than the influence or modification of coexisting primary tauopathies.

A prior report on spinal stenosis, a condition linked to chronic cord compression, noted astrocytic p-tau pathology, mainly in astrocytic processes.^21^ In contrast, the CTE-NC cases in our study showed widespread p-tau–positive astrocytes, a feature absent in spinal stenosis. Notably, the controls with spinal stenosis in our study lacked clear p-tau–positive astrocytes. These distinctions suggest that the spinal cord p-tau pathology in CTE-NC likely reflects a mechanism driven by repetitive central nervous system (CNS) impacts.

While aging-related tau astrogliopathy (ARTAG) in the brain has been linked to TBI and RHI,^28^ spinal ARTAG has not been well studied. We found no evidence of spinal ARTAG-like lesions in the controls aged 65 years or older. In contrast, spinal astrocytic p-tau pathology was more pronounced in the CTE-NC group than in the non–CTE-NC group (Table 1), and likewise in the confirmed RHI group compared with the non-RHI group (eTable 1 in Supplement 1). Taken together, our findings suggest that prominent spinal p-tau cytopathologies represent an underrecognized yet critical feature of the cases with CTE and RHI.

Previously, brain CTE-NC showed 3R tau and 4R tau in neurons and 4R tau in astrocytes.^29^ Surprisingly, spinal astrocytic p-tau in CTE-NC included both isoforms, resembling Pick disease.^30^ These findings raise the possibility that spinal astrocytic tau pathogenesis in CTE-NC shares mechanistic features with that observed in neurons.

Subgroup comparisons revealed that age and exposure history were associated with the severity of spinal pathology. Spinal tau pathology was significantly severe in the confirmed RHI group compared with the non-RHI groups. Furthermore, among individuals with CTE-RHI, those aged 65 years or older exhibited greater spinal tau pathology than those younger than 65 years. These results suggest that older age and higher cumulative repetitive impacts or mechanism of exposure contribute to spinal p-tau pathology. This is consistent with previous brain findings, where age also correlated with p-tau burden.^13^

In individuals with CTE-RHI who were aged 65 years or older, correlation analysis revealed that spinal p-tau pathology was positively associated with both spinal microglial activation and motor symptoms. Notably, inflammatory responses were evident in the spinal cord decades after RHI exposure, aligning with prior findings in CTE- or TBI-affected brains.^31,32^ Given the consistent presence of spinal tau pathology in all CTE-NC cases, these results suggest that CTE may extend beyond the brain to include spinal cord involvement, supporting a broader concept of chronic traumatic encephalomyelopathy. However, motor symptoms in this study primarily involved gait disturbances, which could not be clearly classified as voluntary or involuntary. LMN signs were noted in 4 individuals with CTE-RHI who were aged 65 years or older, but they showed no significant correlation with spinal tau burden; however, interpretation is limited by the small sample size.

Regarding pathogenesis, 2 hypotheses may explain spinal p-tau in CTE: (1) secondary spread from the brain, or (2) independent spinal deposition due to repetitive mild spinal trauma. The lack of correlation between spinal p-tau deposition and brain CTE-NC stage (severity) may be more consistent with the latter. Furthermore, the lack of association between the severity of spinal p-tau in CTE and a history of spinal cord injury, along with its morphological distinction from the p-tau pathology observed in spinal stenosis (chronic compression condition), supports the notion that chronic repetitive impacts to the spinal cord may underlie this pathology. The greater spinal tau pathology in CTE-RHI compared with non-CTE with RHI suggests that CTE-NC development may have a stronger influence than RHI exposure alone. However, this does not imply spinal pathology results from downward spread of CTE-NC. Individuals with CTE-NC may have experienced more intense or repetitive brain and spinal cord impacts than can be captured by concussion counts alone. Although total p-tau burden did not differ significantly, the neuronal p-tau burden was higher in the cervical cord, and the gray matter astrocytic p-tau burden tended to be higher in the lumbar cord. This might reflect the involvement of multiple pathomechanisms contributing to spinal cord pathology.

Beyond p-tau, in the CTE-NC cases, especially in individuals with CTE-RHI who were aged 65 years or older, high rates of spinal p-TDP-43, α-synuclein, and Aβ deposition were observed. In particular, p-tau, p-TDP-43, and α-synuclein exhibited prominent spherical neuritic pathology, reminiscent of the APP-positive spheroids observed in TBI including CTE suggestive of impaired axonal transport.^8,9,33,34,35,36,37,38,39^ However, no clear association was found between the severity of APP-positive spheroids and the overall load of each misfolded protein deposition in this study. Spinal α-synuclein pathology correlated with brain pathology, while p-TDP-43 and Aβ did not, implying possible independent spinal pathology. Indeed, several CTE-NC cases had spinal p-TDP-43 or Aβ without corresponding brain pathology, suggesting a brain-spinal dissociation. Spinal Aβ showed perivascular patterns similar to the distribution of p-tau in the brain.^7^ Given prior evidence of blood-brain barrier dysfunction in brains with CTE or TBI^40,41^ and observed small vessel pathology in spinal cords with CTE, this suggests Aβ deposition may also be associated with vascular barrier dysfunction.

p-TDP-43–positive inclusions were seen in 64% of individuals with CTE-NC who were aged 65 years or older, including skeinlike, compact neuronal inclusions, similar to those seen in motor neuron disease (MND) or ALS. Furthermore, hyalinlike inclusions, commonly seen in ALS,^42^ were observed in the spinal cord of individuals with CTE-NC. However, severe neuronal loss or Bunina bodies were absent, suggesting that spinal cords with CTE may exhibit pathology resembling earliest-stage ALS. Prior work has found an association between trauma and ALS risk, and a subset of CTE-NC cases and MND or ALS cases show overlapping pathology. CTE-MND pathology has been reported in 11.8% of CTE-NC cases,^8^ and CTE-NC has been observed in 5.8% of ALS cases.^43^ Noteworthy, the positive neuron was also observed in 1 case of non-CTE with RHI. Our study focusing on p-TDP-43–positive inclusions could further reinforce the association between CTE or RHI and p-TDP-43 pathology.

### Limitations

This study demonstrated prominent spinal protein pathology—particularly tau—in CTE-NC cases, although several limitations should be considered. Postmortem studies like ours are inherently subject to selection bias and confounding factors, as they include only individuals who undergo autopsy, and the influence of coexisting neurodegenerative diseases cannot be completely ruled out. Many CTE cases had coexisting neurodegenerative brain pathologies, which may have influenced spinal findings. To minimize this, we compared brain and spinal pathology and analyzed pure CTE-NC cases without other primary tauopathies. Some cases also showed spinal-only TDP-43 pathology, suggesting distinct mechanisms. Larger studies focusing on pure CTE-NC cohorts are needed. Additionally, clinical data were limited by the retrospective design. Lifetime concussion counts and repetitive back impact histories were unavailable, and most neurological examinations related to spinal cord function were either single-time assessments or missing. More comprehensive longitudinal neurological assessments over time in individuals with CTE or RHI might have better delineated spinal cord–related neurological signs. Future large-scale studies should aim to clarify clinicopathological correlations, including detailed assessments of CNS trauma type (head vs back impacts) and spinal pathology distribution (cervical, thoracic, or lumbar).

## Conclusions

This case-control study highlights the spinal cord as a key site to develop protein pathology in cases with CTE-NC in the brain. Associations between spinal p-tau pathology, focal inflammation, and motor symptoms suggest that the concept of chronic repetitive trauma affecting the CNS may need to be expanded from being brain-focused CTE to encompassing the spinal cord as well, namely, chronic trauma-induced encephalomyelopathy. Chronic repetitive trauma to the CNS may accelerate age-related neurodegeneration, leading to the deposition of multiple misfolded proteins in both brain and spinal cord. These findings highlight the need to reconsider the spinal cord as a target of repetitive impacts and to further explore its clinical relevance in individuals with histories of repetitive CNS trauma.

## Notes

Supplement 1. **eMethods.** Supplemental Methods **eResults.** Supplemental Results **eFigure 1.** Overview of Cohort in This Study **eFigure 2.** Study Design and Analytical Subgroups Used in This Study **eFigure 3.** Representative Spinal Cord Sections Used in This Study (Transverse and Coronal Sections) **eFigure 4.** Representative Spinal Cord Pathology in nonCTE-RHI Cases **eFigure 5.** Astrocytic Tau Pathology in the Spinal Cord of CTE-NC Cases **eFigure 6.** Neuronal Tau Predominant Lesion in the Spinal Cord of CTE-NC Case **eFigure 7.** Representative Tau Lesions With a Classical 3R/4R Isoform Distribution **eFigure 8.** Representative Photomicrographs of 3R-Tau and 4R-Tau-Positive Astrocytes **eFigure 9.** Representative Photomicrographs of 3R-Tau and 4R-Tau-Positive Threads in the Spinal White Matter **eFigure 10.** Representative p-Tau Immunoreactivity in the Spinal Cord of Aged Controls **eFigure 11.** Regional Comparison of Spinal Cord Tau Pathology in CTE-RHI Cases Aged ≥65 Years **eFigure 12.** Representative p-TDP-43 Immunopositive Inclusions in CTE-NC Cases **eFigure 13.** Representative Photomicrographs of the Anterior Horn in CTE-NC Cases **eFigure 14.** Representative Spinal Cord Pathology in CTE-NC Cases From Indeterminate RHI Group **eFigure 15.** Representative p-Tau Lesions in CTE-RHI Cases Aged <65 Years **eFigure 16.** Representative APP Staining of the Spinal Cord in CTE-NC Cases **eFigure 17.** Representative HLA-DR and APP Staining in Aged Control Cases **eFigure 18.** Small Vessel Changes in the Spinal Cord in CTE-NC Cases **eFigure 19.** Small Vessel Changes in the Spinal Cord in Controls **eFigure 20.** Spinal Cord Pathology Correlation Heatmap in CTE-RHI Cases Aged ≥65 Years **eTable 1.** Summary of Clinicopathological Characteristics in Groups of RHI Cohort **eTable 2.** Summary of Antibodies Used in This Study **eTable 3.** Comparison of CTE-RHI vs nonCTE-RHI Cases in Confirmed RHI Group **eTable 4A.** Severity of Spinal Cord Pathology at Each Spinal Level in CTE-RHI Cases Aged ≥65 **eTable 4B.** Summary of Spinal Cord Pathology by Case in CTE-RHI Cases Aged ≥65 **eTable 5.** Summary of Major Clinicopathological Findings in Controls (nonCTE-nonRHI) **eTable 6.** Tau Pathology of Spinal Cord at Each Level in Control Group (nonCTE-nonRHI) **eTable 7.** Subgroup Comparison in CTE-NC Cases **eTable 8.** Summary of Major Clinicopathological Findings in CTE-NC Cases From Indeterminate RHI Group **eTable 9.** Tau Pathology of Spinal Cord at Each Level in CTE-NC Cases From Indeterminate RHI Group **eTable 10.** Summary of Major Clinicopathological Findings in Non-CTE-NC Cases From Indeterminate RHI Group **eTable 11.** Tau Pathology of Spinal Cord at Each Level in p-Tau-Positive 11 Non-CTE-NC Cases From Indeterminate RHI Group **eTable 12.** Summary of Clinicopathological Characteristics in Indeterminate RHI Group **eTable 13.** Summary of Major Clinicopathological Findings in CTE-RHI Cases Aged <65 **eTable 14.** Severity of Spinal Cord Pathology at Each Level in CTE-RHI Cases Aged <65 **eTable 15.** Summary of Major Clinicopathological Findings in nonCTE-RHI Cases **eTable 16.** Severity of Spinal Cord Pathology at Each Level in nonCTE-RHI Cases **eTable 17.** Correlation Between Spinal Cord and Brain Pathologies in CTE-RHI Cases Aged ≥65 **eTable 18.** Comparison of CTE-NC Cases With and Without Comorbid Primary Tauopathies **eTable 19.** Clinical Features in All 23 Cases in Confirmed RHI Group **eTable 20.** Clinicopathological Correlation in CTE-RHI Cases Aged ≥65
eReferences

Supplement 2. Data Sharing Statement
