# Supplementary material for: Spinal Cord Tau and Protein Copathologies Associated With Chronic Traumatic Encephalopathy
Source: JAMA Neurol. 2026 Jan 26;83(3):231–41. doi: 10.1001/jamaneurol.2025.5421 (PMC12836280; doi:10.1001/jamaneurol.2025.5421)
Supplement: Supplement 1. — eMethods. Supplemental Methods eResults. Supplemental Results eFigure 1. Overview of Cohort in This Study eFigure 2. Study Design and Analytical Subgroups Used in This Study eFigure 3. Representative Spinal Cord Sections Used in This Study (Transverse and Coronal Sections) eFigure 4. Representative Spinal Cord Pathology in nonCTE-RHI Cases eFigure 5. Astrocytic Tau Pathology in the Spinal Cord of CTE-NC Cases eFigure 6. Neuronal Tau Predominant Lesion in the Spinal Cord of CTE-NC Case eFigure 7. Representative Tau Lesions With a Classical 3R/4R Isoform Distribution eFigure 8. Representative Photomicrographs of 3R-Tau and 4R-Tau-Positive Astrocytes eFigure 9. Representative Photomicrographs of 3R-Tau and 4R-Tau-Positive Threads in the Spinal White Matter eFigure 10. Representative p-Tau Immunoreactivity in the Spinal Cord of Aged Controls eFigure 11. Regional Comparison of Spinal Cord Tau Pathology in CTE-RHI Cases Aged ≥65 Years eFigure 12. Representative p-TDP-43 Immunopositive Inclusions in CTE-NC Cases eFigure 13. Representative Photomicrographs of the Anterior Horn in CTE-NC Cases eFigure 14. Representative Spinal Cord Pathology in CTE-NC Cases From Indeterminate RHI Group eFigure 15. Representative p-Tau Lesions in CTE-RHI Cases Aged <65 Years eFigure 16. Representative APP Staining of the Spinal Cord in CTE-NC Cases eFigure 17. Representative HLA-DR and APP Staining in Aged Control Cases eFigure 18. Small Vessel Changes in the Spinal Cord in CTE-NC Cases eFigure 19. Small Vessel Changes in the Spinal Cord in Controls eFigure 20. Spinal Cord Pathology Correlation Heatmap in CTE-RHI Cases Aged ≥65 Years eTable 1. Summary of Clinicopathological Characteristics in Groups of RHI Cohort eTable 2. Summary of Antibodies Used in This Study eTable 3. Comparison of CTE-RHI vs nonCTE-RHI Cases in Confirmed RHI Group eTable 4A. Severity of Spinal Cord Pathology at Each Spinal Level in CTE-RHI Cases Aged ≥65 eTable 4B. Summary of Spinal Cord Pathology by Case in CTE-RHI Cas [file jamaneurol-e255421-s001.pdf]

Supplemental Online Content

Tanaka H, Black LE, Forrest SL, et al. Spinal cord tau and protein copathologies associated with chronic traumatic encephalopathy. *JAMA Neurol*. Published online January 26, 2026. doi:1.1001/jamaneurol.2025.5421

eMethods. Supplemental Methods
eResults. Supplemental Results
eFigure 1. Overview of Cohort in This Study
eFigure 2. Study Design and Analytical Subgroups Used in This Study
eFigure 3. Representative Spinal Cord Sections Used in This Study (Transverse and Coronal Sections)
eFigure 4. Representative Spinal Cord Pathology in nonCTE-RHI Cases
eFigure 5. Astrocytic Tau Pathology in the Spinal Cord of CTE-NC Cases
eFigure 6. Neuronal Tau Predominant Lesion in the Spinal Cord of CTE-NC Case
eFigure 7. Representative Tau Lesions With a Classical 3R/4R Isoform Distribution
eFigure 8. Representative Photomicrographs of 3R-Tau and 4R-Tau-Positive Astrocytes
eFigure 9. Representative Photomicrographs of 3R-Tau and 4R-Tau-Positive Threads in the Spinal White Matter
eFigure 10. Representative p-Tau Immunoreactivity in the Spinal Cord of Aged Controls
eFigure 11. Regional Comparison of Spinal Cord Tau Pathology in CTE-RHI Cases Aged ≥65 Years
eFigure 12. Representative p-TDP-43 Immunopositive Inclusions in CTE-NC Cases
eFigure 13. Representative Photomicrographs of the Anterior Horn in CTE-NC Cases
eFigure 14. Representative Spinal Cord Pathology in CTE-NC Cases From Indeterminate RHI Group
eFigure 15. Representative p-Tau Lesions in CTE-RHI Cases Aged <65 Years
eFigure 16. Representative APP Staining of the Spinal Cord in CTE-NC Cases
eFigure 17. Representative HLA-DR and APP Staining in Aged Control Cases
eFigure 18. Small Vessel Changes in the Spinal Cord in CTE-NC Cases
eFigure 19. Small Vessel Changes in the Spinal Cord in Controls
eFigure 20. Spinal Cord Pathology Correlation Heatmap in CTE-RHI Cases Aged ≥65 Years
eTable 1. Summary of Clinicopathological Characteristics in Groups of RHI Cohort
eTable 2. Summary of Antibodies Used in This Study
eTable 3. Comparison of CTE-RHI vs nonCTE-RHI Cases in Confirmed RHI Group
eTable 4A. Severity of Spinal Cord Pathology at Each Spinal Level in CTE-RHI Cases Aged ≥65
eTable 4B. Summary of Spinal Cord Pathology by Case in CTE-RHI Cases Aged ≥65
eTable 5. Summary of Major Clinicopathological Findings in Controls (nonCTE-nonRHI)
eTable 6. Tau Pathology of Spinal Cord at Each Level in Control Group (nonCTE-nonRHI)

© 2026 Tanaka H et al. *JAMA Neurology*.

eTable 7. Subgroup Comparison in CTE-NC Cases
eTable 8. Summary of Major Clinicopathological Findings in CTE-NC Cases From Indeterminate RHI Group
eTable 9. Tau Pathology of Spinal Cord at Each Level in CTE-NC Cases From Indeterminate RHI Group
eTable 10. Summary of Major Clinicopathological Findings in Non-CTE-NC Cases From Indeterminate RHI Group
eTable 11. Tau Pathology of Spinal Cord at Each Level in p-Tau-Positive 11 Non-CTE-NC Cases From Indeterminate RHI Group
eTable 12. Summary of Clinicopathological Characteristics in Indeterminate RHI Group
eTable 13. Summary of Major Clinicopathological Findings in CTE-RHI Cases Aged <65
eTable 14. Severity of Spinal Cord Pathology at Each Level in CTE-RHI Cases Aged <65
eTable 15. Summary of Major Clinicopathological Findings in nonCTE-RHI Cases
eTable 16. Severity of Spinal Cord Pathology at Each Level in nonCTE-RHI Cases
eTable 17. Correlation Between Spinal Cord and Brain Pathologies in CTE-RHI Cases Aged ≥65
eTable 18. Comparison of CTE-NC Cases With and Without Comorbid Primary Tauopathies
eTable 19. Clinical Features in All 23 Cases in Confirmed RHI Group
eTable 20. Clinicopathological Correlation in CTE-RHI Cases Aged ≥65
eReferences

This supplemental material has been provided by the authors to give readers additional information about their work.

© 2026 Tanaka H et al. *JAMA Neurology*.

eMethods. Supplemental Methods

Study Design

To examine the impact of RHI exposure and CTE-NC status on spinal cord pathology, cases were grouped according to both factors. Multiple datasets were created based on their combinations to determine whether spinal cord pathology was more closely associated with RHI exposure itself or with the development of CTE-NC. The subgroup with both RHI exposure and CTE-NC (CTE-RHI) was analyzed in particular, while cases without either served as controls (nonCTE-nonRHI). Cases with confirmed RHI but without CTE-NC (nonCTE-RHI) were used to assess the effects of RHI itself on the spinal cord. Considering prior evidence that age influences CTE-NC severity in the brain,[1] age-stratified analyses were also performed (**eFigures 1, 2**).

Subjects

We investigated spinal cords from a total of 70 individuals, 20 with autopsy-confirmed chronic traumatic encephalopathy neuropathologic change (CTE-NC) and 50 without. These individuals were primarily drawn from the cohort based on the history of exposure to repetitive head impacts (RHI): **Confirmed RHI** (contact sports participation or military service, n=23), **Indeterminate RHI** (individuals from the homeless population, n=34), and **Non-RHI** (n=13).

Confirmed RHI group included 23 individuals, who had a well-documented history of RHI through participation in contact sports or military service from North-America and the UK. Among them, 16 showed CTE-NC of the brain (70%, CTE-RHI group). The remaining 7 RHI cases did not meet CTE-NC criteria[2] and were categorized as "nonCTE-RHI".

Indeterminate RHI group consisted of 34 individuals, aged 41-67, from the Central European homeless population without contact sport or military history. Although these individuals had no documented history of RHI exposure, potential exposure to head trauma could not be excluded. Nevertheless, the overall risk and nature of traumatic impacts were presumed to be lower and qualitatively different from those observed in the Confirmed RHI group. Among these, four cases showed CTE-NC in the brain (11.76%).[3]

Non-RHI group consisted of 13 individuals from North-America and the UK, none of whom exhibited CTE-NC in the brain (nonCTE-nonRHI), which served as controls. Six individuals aged ≥65 years showed no significant neurodegenerative pathology. The remaining seven individuals exhibited high Alzheimer's disease neuropathologic change (AD-NC). Four of the non-RHI cases had spinal stenosis.

Neuropathological examination

Histopathological analysis was carried out using formalin-fixed, paraffin-embedded, 4.5-µm-thick sections from the spinal cord (cervical, thoracic, and lumbar/sacral segments) and brain regions required for staging of CTE-NC and mixed pathologies[2,4-8]. Particularly for the spinal cord, coronal (longitudinal) sections were prepared at the level of the anterior horn, in addition to standard transverse sections, to facilitate more detailed evaluation. This approach enabled assessment of a broader portion of the anterior horn, encompassing a greater number of anterior horn cells/motor neurons than is typically possible with transverse sections alone (**eFigure 3**).

Histological examination was performed using hematoxylin and eosin with Luxol fast blue (HE-LFB). Immunohistochemistry was performed using for phosphorylated tau (p-tau), 3R-tau, 4R-tau, p-TDP-

© 2026 Tanaka H et al. *JAMA Neurology*.

43, α-synuclein, β-amyloid (Aβ), amyloid precursor protein (APP), and HLA-DR (microglia marker). A complete list of the primary antibodies used in this study, including pretreatment conditions and dilution factors, is provided in **eTable 2**. Immunostaining was primarily performed using the Dako Autostainer Link 48 and EnVision FLEX+ Visualization System, according to manufacturer's instructions. A subset of sections was manually stained using standard protocols. Subsequently, all sections were counterstained with hematoxylin.

The severity of each spinal cord pathology was assessed using the semi-quantitative scale defined below. Tau-Total (severity of total p-tau pathology), Aβ: 0(-) = absent, 1(+) = minimal, 2(++) = mild, 3(+++) = moderate, 4(++++) = severe; p-TDP-43: 0(-) = absent, 1(+) = present, 2(++) = present with neuronal cytoplasmic inclusions; α-Syn, APP: 0(-) = absent, 1(+) = minimal/mild, 2(++) = moderate, 3(+++) = severe; Tau-N (neuronal p-tau pathology), A-GM (astrocytic p-tau pathology in the gray matter), A-WM (astrocytic p-tau pathology in the white matter), HLA-DR: 0 = absent, 1 = minimal, 2 = mild, 3 = moderate, 4 = severe, 5 = extremely severe.

Retrospective Clinical Data Acquisition

Clinical information was retrospectively obtained through telephone interviews with informants of brain donors and review of medical records. Structured postmortem interviews were conducted by clinicians experienced in neurodegenerative diseases or by trained research assistants. Some athletes additionally participated in a longitudinal program with premortem neurological and neuropsychological assessments. The informant interviews inquired about concussion, medical and psychiatric history, and motor, sensory, and constitutional symptoms.

Statistical analysis

Statistical analyses were performed using GraphPad Prism version 10.4.1 (Dotmatics, Boston, MA, USA). A significance threshold of $p < 0.05$ (two-tailed) was applied. Fisher's exact test was used for categorical variables, and the Mann–Whitney U test was used for ordinal and continuous variables. For comparisons among multiple groups, the Kruskal–Wallis test was applied, followed by post-hoc analysis with Dunn's test.

Correlations between variables were assessed using two-tailed non-parametric Spearman's rank correlation, based on the data presented in **eTable 4A** (for spinal cord pathology correlations); **eTable 4B** and **eTable 19** (for clinicopathological correlations); and **Table 2** and **eTable 4B** (for correlations between spinal cord and brain pathologies).

For the clinical findings of number of concussions, spinal cord injury, and motor symptoms (in **eTable 19**), a semi-quantitative weighting system was applied as follows: Number of concussions was categorized based on available descriptions. Since the most affected cases were described only as "multiple" without an exact count, we defined: (+) = 1–3 episodes; (++) = 4–9; (+++) = 10–20; (++++) >20; (+++++) = multiple (exact number unknown). Spinal cord injury was scored as (+-) when symptoms possibly related to injury could not be ruled out, and (+) when a history of definitive spinal cord/spinal column injury or surgery was present. Motor symptoms in this study encompassed both voluntary and involuntary movement disorders, which are often difficult to clearly distinguish. Therefore, we scored cases as "(+-)" when any motor symptoms, including involuntary movements, were present. Cases were scored as "(+)" when motor

© 2026 Tanaka H et al. *JAMA Neurology*.

symptoms more suggestive of spinal cord involvement—such as lower motor neuron (LMN) signs or gait disturbances—were observed. This scoring approach aimed to selectively identify motor symptoms potentially associated with spinal cord pathology. One case of CTE-NC with concomitant cerebral infarction was designated as “not available” for motor symptoms to avoid misclassification.

For brain pathology (mixed pathology) in **Table 2**, the staging of each disease entity was treated as an ordinal variable, following the established hierarchical order of severity. An exception was made for CAA (and SAA), which was scored as 1 for Type 2 (amyloid deposition limited to small vessels only) and 2 for Type 1 (amyloid deposition in both small vessels and capillaries). In the case of LBD, cases with the amygdala-predominant or amygdala-only type were scored as 1.

Regional differences in spinal cord tau pathology were evaluated using the Friedman test, followed by post-hoc analysis with Dunn’s test (**eFigure 11**). To evaluate the relative predominance of astrocytic over neuronal tau pathology, we calculated the A/N ratio (astrocytic tau score divided by neuronal tau score) at each spinal cord level. In cases where both scores were zero (0/0), the ratio was considered undefined and treated as missing. For instances of division by zero, where the neuronal tau score was zero and the astrocytic tau score was greater than zero, a fixed value of 5 was substituted, reflecting the highest valid A/N ratio observed in the dataset. This substitution strategy was applied to preserve the interpretability of the ratio-based analysis while avoiding distortion from undefined or infinite values. When the numerator was zero and the denominator was non-zero (i.e., astrocytic tau = 0 and neuronal tau > 0), the ratio was defined as 0.

© 2026 Tanaka H et al. *JAMA Neurology*.

eResults. Supplemental Results

Absence of histopathological evidence of prior major or severe spinal injury

All spinal cord sections included in this study were histologically examined for evidence of previous acute or major traumatic injury. Specifically, the presence of hemosiderin deposition within the spinal parenchyma, indicative of prior hemorrhagic lesions, as well as large necrotic or infarcted areas, was assessed on H&E sections. These evaluations aimed to identify possible sequelae of past spinal trauma that could influence the interpretation of spinal protein pathology. No such lesions suggestive of prior hemorrhage, necrosis, or infarction were observed in any of the examined sections.

Spinal Cord Pathology in the CTE-NC cases from the Indeterminate RHI Group

CTE-NC was diagnosed in 4 cases (4/34, 11.76%; Age range: 56-67, mean 60.75). All four cases showed spinal cord p-tau pathology; neuronal in all (100%) and astrocytic in three (75%) (**eTables 8, 9**). While the overall patterns resembled that seen in CTE-RHI cases, p-tau pathology was milder (mean total tau score: 1.08 vs 1.71; **eTable 7**). p-TDP-43-positive neurons were present in one case (**eFigure 14**). α-Synuclein pathology was absent. Compared to non-CTE cases in the Indeterminate RHI group (**eTables 10, 11**), cases with CTE-NC in the brain showed significantly higher total p-tau burden ($p < 0.0001$) and frequencies of tau-positive neurons ($p < 0.01$) and astrocytes ($p < 0.01$) in the spinal cord (**eTable 12**).

Spinal Cord Pathology in CTE-RHI cases aged <65 years

Two younger individuals (44 and 50 years) with professional contact sports history were investigated to assess age effects. Both had spinal p-tau pathology, but severity was lower (mean tau score: 0.50) than in CTE-RHI cases aged ≥65 years (**eFigure 15; eTable 7**). p-TDP-43 and α-synuclein pathologies were absent, but Aβ deposition was present in the spinal cord despite low AD-NC in the brain (**eTables 13, 14**).

Tau isoform of the neuronal and astrocytic tau lesions in the spinal cord of CTE-NC cases

Tau isoforms (3R-tau, 4R-tau) in spinal tau pathology of CTE-NC cases were examined using serial sections. As a result, we observed both lesions consistent with previously reported patterns and those showing differing features. In lesions consistent with earlier reports, tau positive neurons were positive for both 3R- and 4R-tau, whereas tau-positive astrocytes were negative for 3R-tau and positive for 4R-tau (**eFigure 7**).

Interestingly, however, upon careful evaluation, some astrocytic tau lesions were found to be positive for both 3R- and 4R-tau (**eFigure 8**).

Furthermore, many tau-positive threads in the white matter were also immunoreactive for both 3R- and 4R-tau (**eFigure 9**).

Severity of the tau pathology at each spinal cord level (total, neuronal, astrocytic) in CTE-RHI cases aged ≥65 years

Of all tau pathologies assessed, including total, neuronal, and astrocytic tau in both gray and white matter, only neuronal tau showed a significant variation among cervical, thoracic, and lumbar levels (P = 0.0161, Friedman test). Post-hoc analysis using Dunn's test indicated no statistically significant pairwise differences

© 2026 Tanaka H et al. *JAMA Neurology*.

after multiple comparison correction, but a trend toward higher neuronal tau burden in the cervical compared to lumbar level was observed (P = 0.099, Z = 2.13), suggesting potential regional variation (**eFigure 11A**).

Although astrocytic tau pathology at each spinal cord level did not show statistically significant differences on its own, its distribution appeared visually distinct from that of neuronal tau pathology. To further investigate this potential disparity, we analyzed the relative burden of astrocytic to neuronal tau pathology by calculating the A-GM/N ratio (astrocytic tau score in gray matter divided by neuronal tau score) at each spinal cord level.

The A-GM/N ratio varied significantly across spinal levels according to the Friedman test (P = 0.0226). Post-hoc Dunn’s tests did not reveal statistically significant pairwise differences after multiple comparison correction; however, the cervical vs lumbar comparison demonstrated a trend toward significance (adjusted P = 0.0975, Z = 2.14), suggesting a possible regional shift in tau subtype predominance (**eFigure 11B**).

© 2026 Tanaka H et al. *JAMA Neurology*.

eFigure 1. Overview of Cohort in This Study

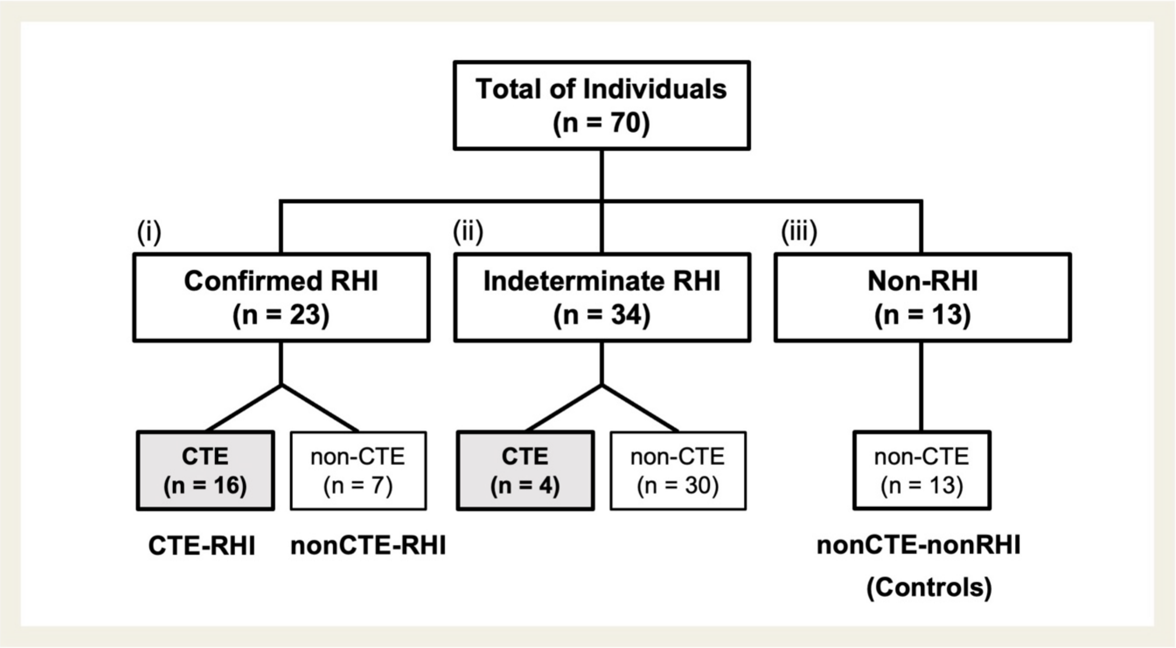

Abbreviations: CTE-NC, chronic traumatic encephalopathy neuropathologic change; RHI, repetitive head impacts; nonCTE-RHI, RHI case did not meet CTE-NC criteria[2].

© 2026 Tanaka H et al. *JAMA Neurology*.

eFigure 2. Study Design and Analytical Subgroups Used in This Study

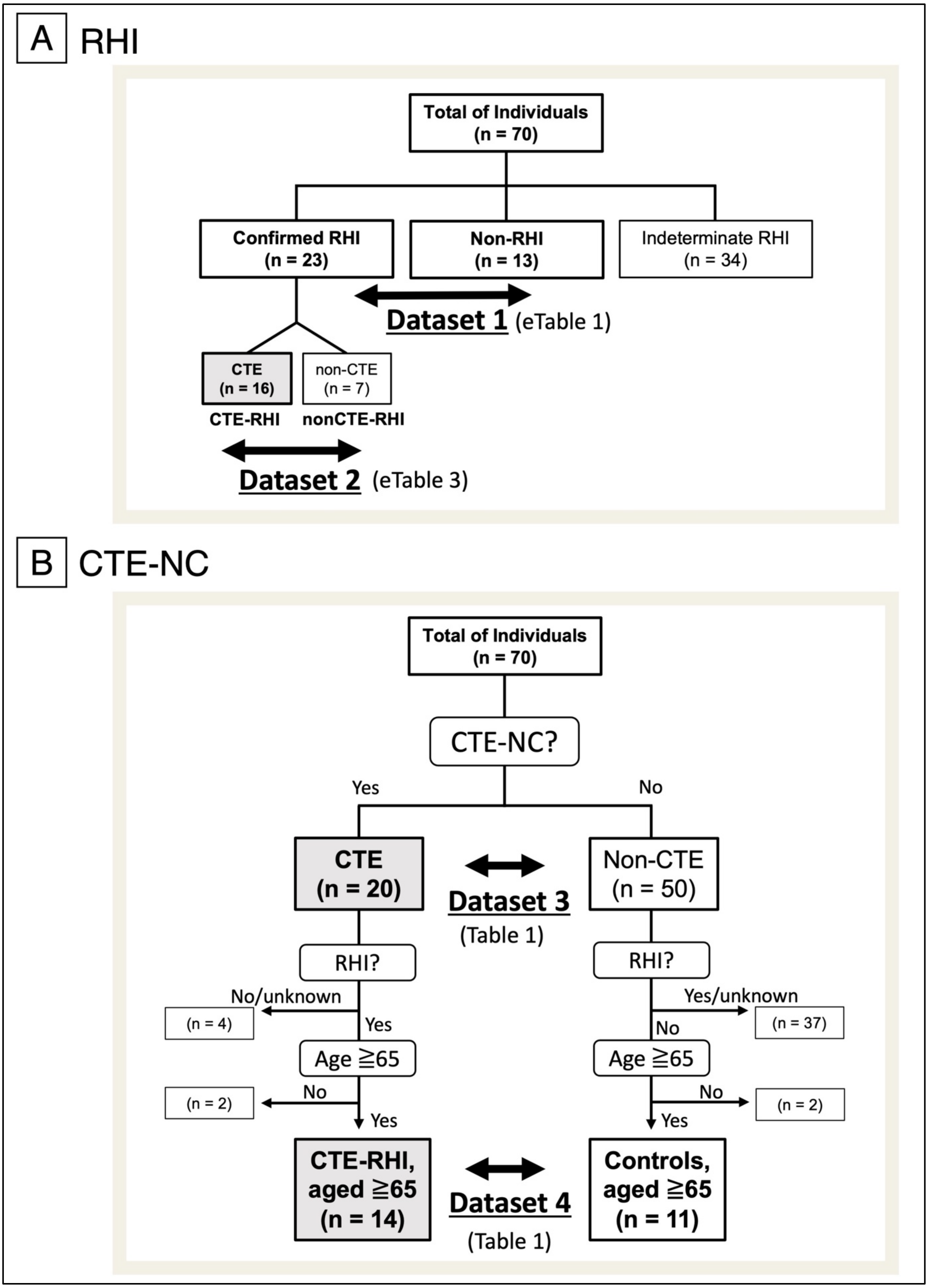

Abbreviations: CTE-NC, chronic traumatic encephalopathy neuropathologic change; RHI, repetitive head impacts; nonCTE-RHI, RHI case did not meet CTE-NC criteria[2].

© 2026 Tanaka H et al. *JAMA Neurology*.

eFigure 3. Representative Spinal Cord Sections Used in This Study (Transverse and Coronal Sections)

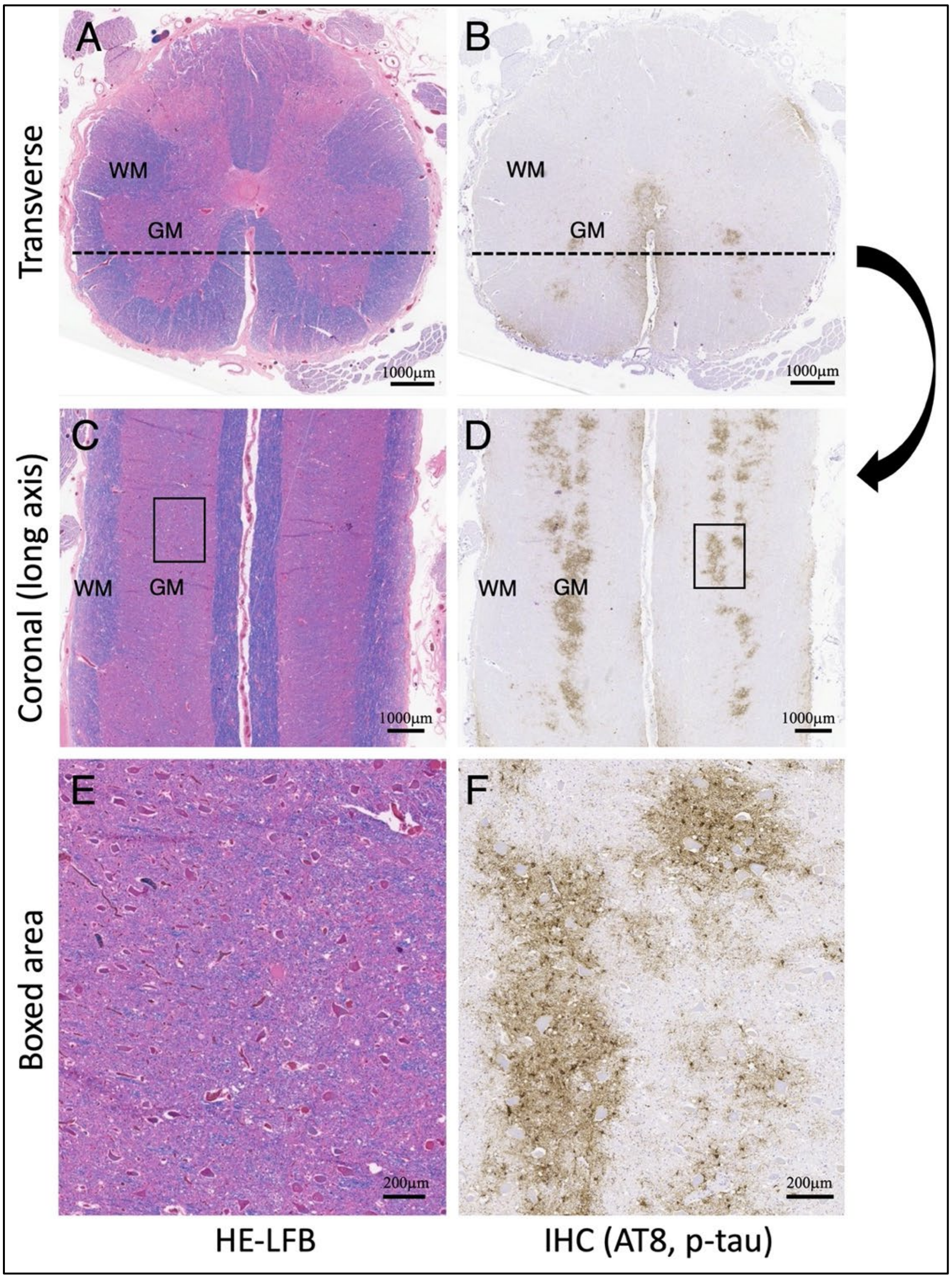

In this study, in addition to standard transverse sections (A, B), coronal (longitudinal) sections (C, D) were prepared at the anterior horn level (dotted lines in A and B) and evaluated. This approach allowed for assessment of a broader portion of the anterior horn containing a larger number of anterior horn cells/motor neurons than is typically possible with transverse sections alone (E, F). GM = gray matter; WM = white matter.
(A–F) CTE #7, lumbar cord. (A, C, E) HE-LFB, (B, D, F) AT8.

© 2026 Tanaka H et al. *JAMA Neurology*.

eFigure 4. Representative Spinal Cord Pathology in nonCTE-RHI Cases

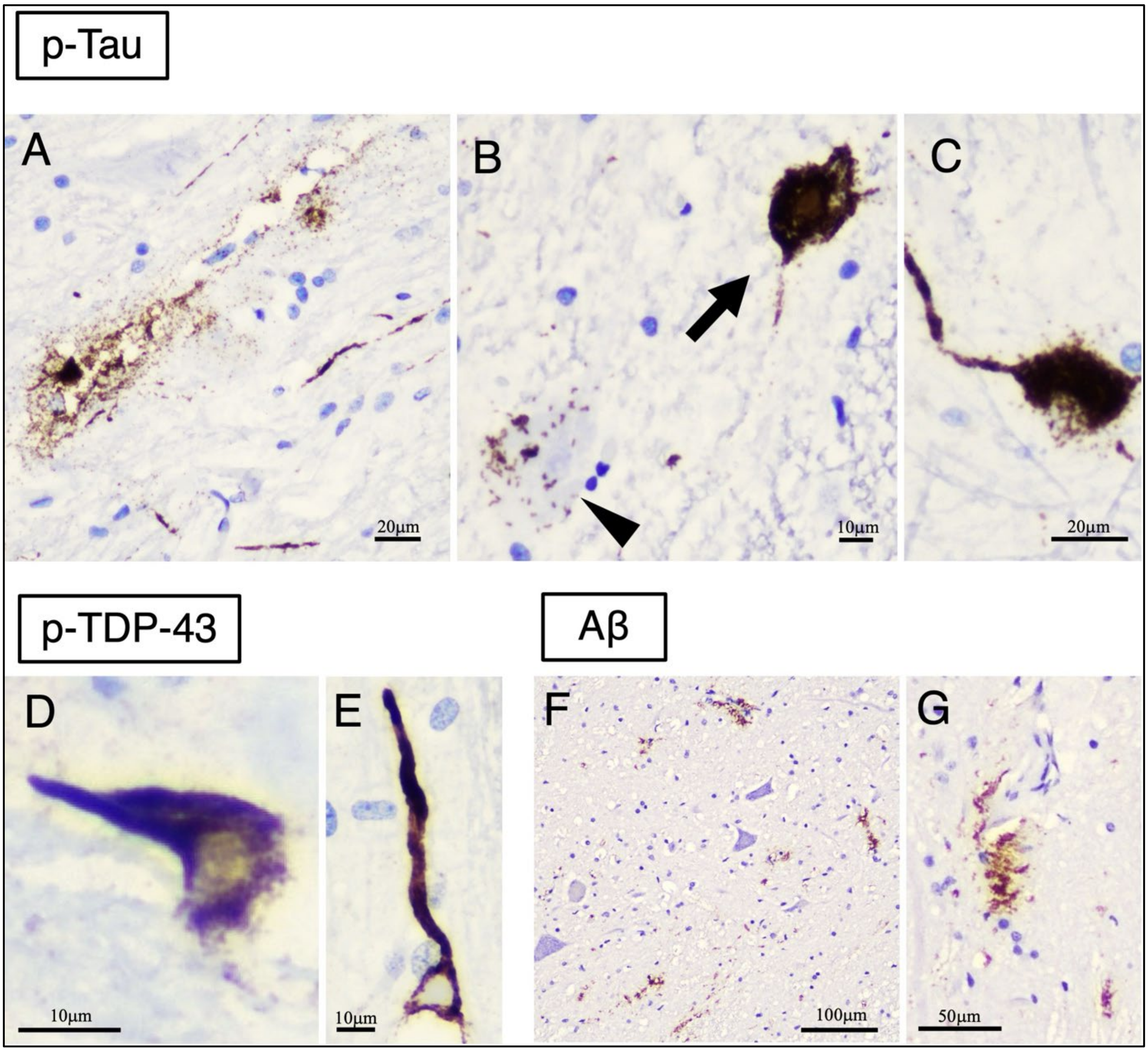

(A) Astrocytic tau pathology in the gray matter. (B, C) Neuronal tau pathology in the gray matter (arrow: neurofibrillary tangle, arrowhead: pre-tangle morphology). (D, E) p-TDP-43-positive neuron (D) and neurites (E) in the anterior horn (F, G) Amyloid-β depositions in the anterior horn.
(A-C) nonCTE-RHI #7 (D,E) nonCTE-RHI #3 (F, G) nonCTE-RHI #5. (A-C) AT8, (D, E) p-TDP-43, (F, G) Aβ.

© 2026 Tanaka H et al. *JAMA Neurology*.

eFigure 5. Astrocytic Tau Pathology in the Spinal Cord of CTE-NC Cases

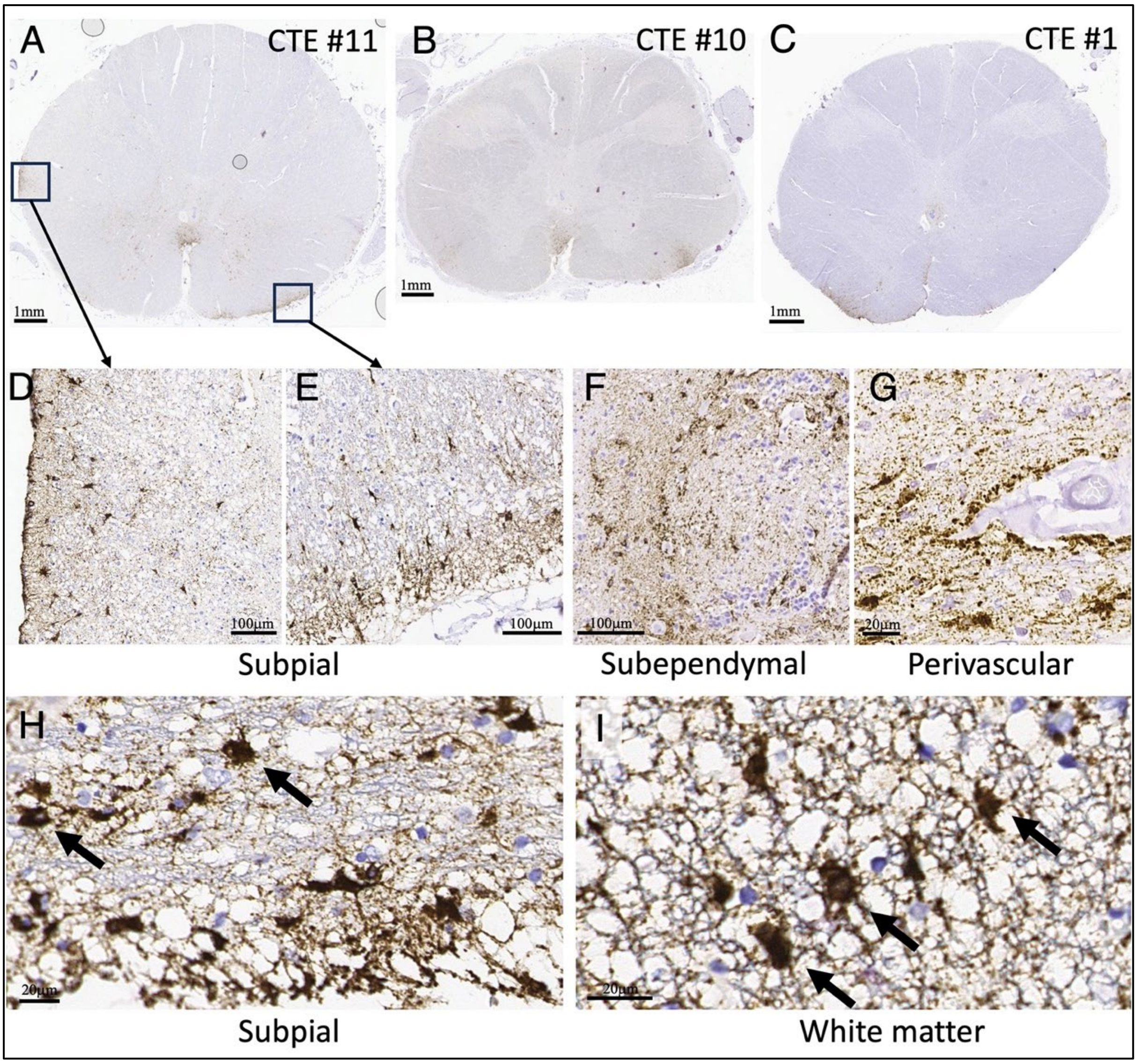

(A–C) Low-magnification images of the spinal cord show phosphorylated abnormal tau (p-tau) deposits in the white matter, especially in the ventral and/or lateral subpial regions. These deposits are composed of abundant tau-positive astrocytes (D, E, H, I). CTE-NC cases frequently exhibit astrocytic tau pathology in the subpial (D, E), subependymal (F), perivascular (G), and deep white matter regions (I). Morphologically, the tau-positive astrocytes are identified as "thorn-shaped" astrocytes (H, I).
(A-I) AT8.

© 2026 Tanaka H et al. *JAMA Neurology*.

eFigure 6. Neuronal Tau Predominant Lesion in the Spinal Cord of CTE-NC Case

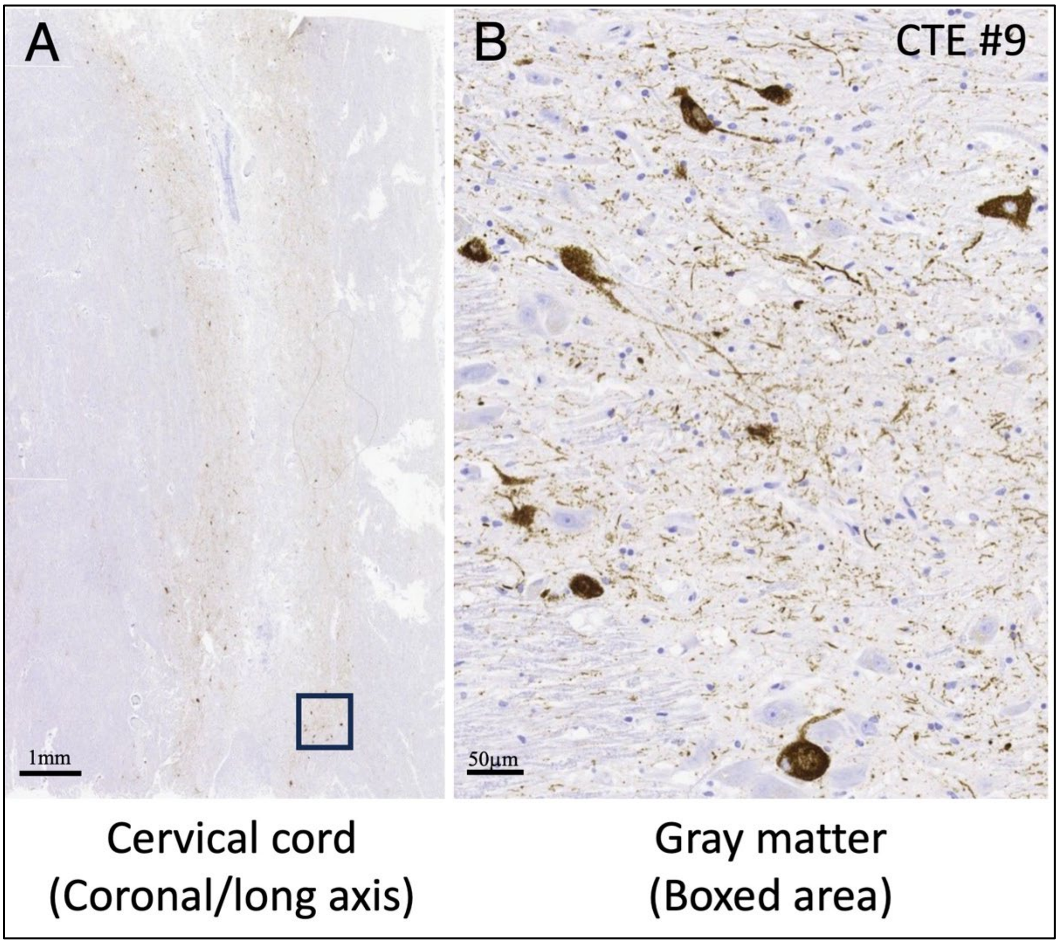

(A) Low magnification of the cervical cord in CTE #9. (B) Boxed area in A. There are relatively many p-tau-positive neurons in the gray matter/anterior horn, while astrocytic tau pathology is not prominent.
(A, B) AT8.

© 2026 Tanaka H et al. *JAMA Neurology*.

eFigure 7. Representative Tau Lesions With a Classical 3R/4R Isoform Distribution

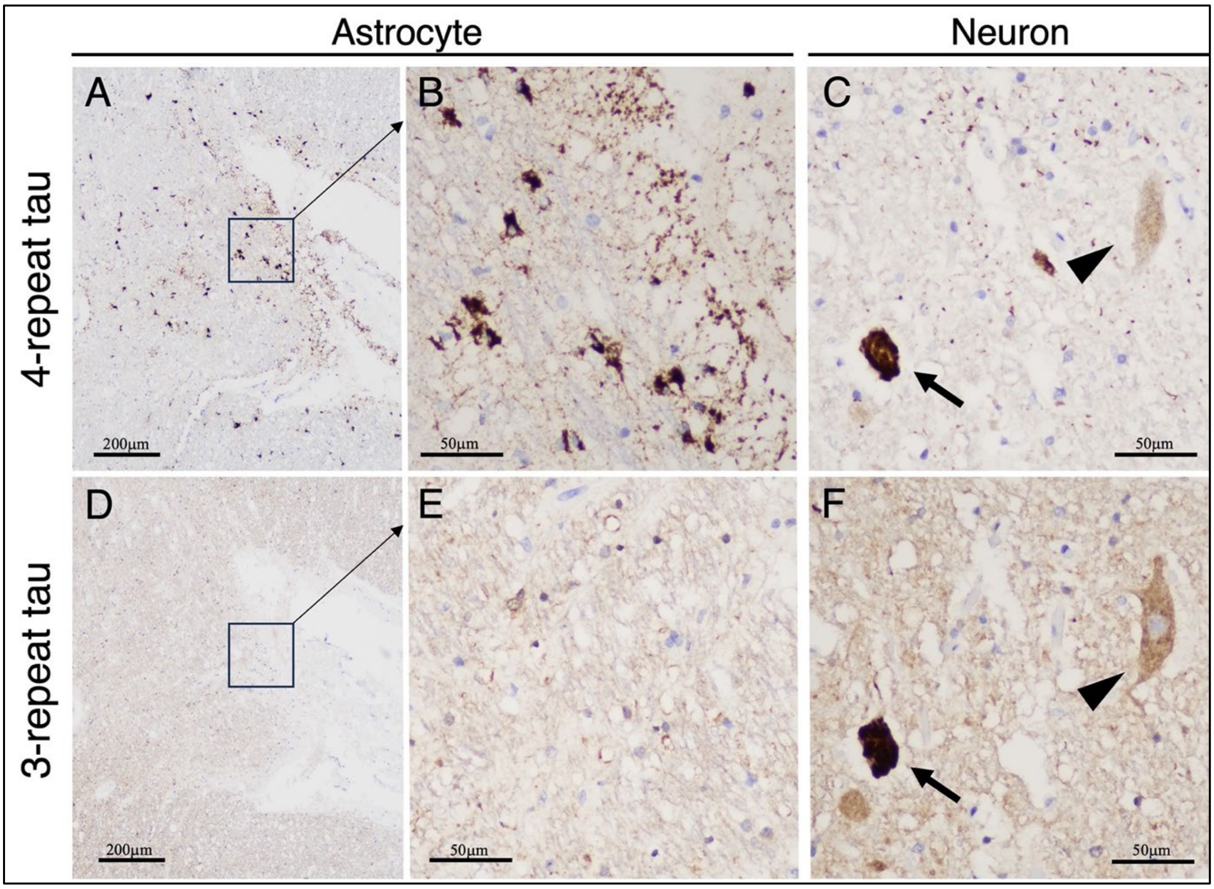

Tau isoform evaluation using serial sections (4R-tau: A–C; 3R-tau: D–F) revealed 4R-tau-positive but 3R-tau-negative astrocytes in the white matter and subpial regions (A, B, D, E). In addition, in the anterior horn (C, F), neurons positive for both 4R- and 3R-tau were observed (arrow: neuronal cytoplasmic inclusions; arrowhead: adjacent tau-negative neurons.
(A-C) RD4, (D-F) RD3.

© 2026 Tanaka H et al. *JAMA Neurology*.

eFigure 8. Representative Photomicrographs of 3R-Tau and 4R-Tau-Positive Astrocytes

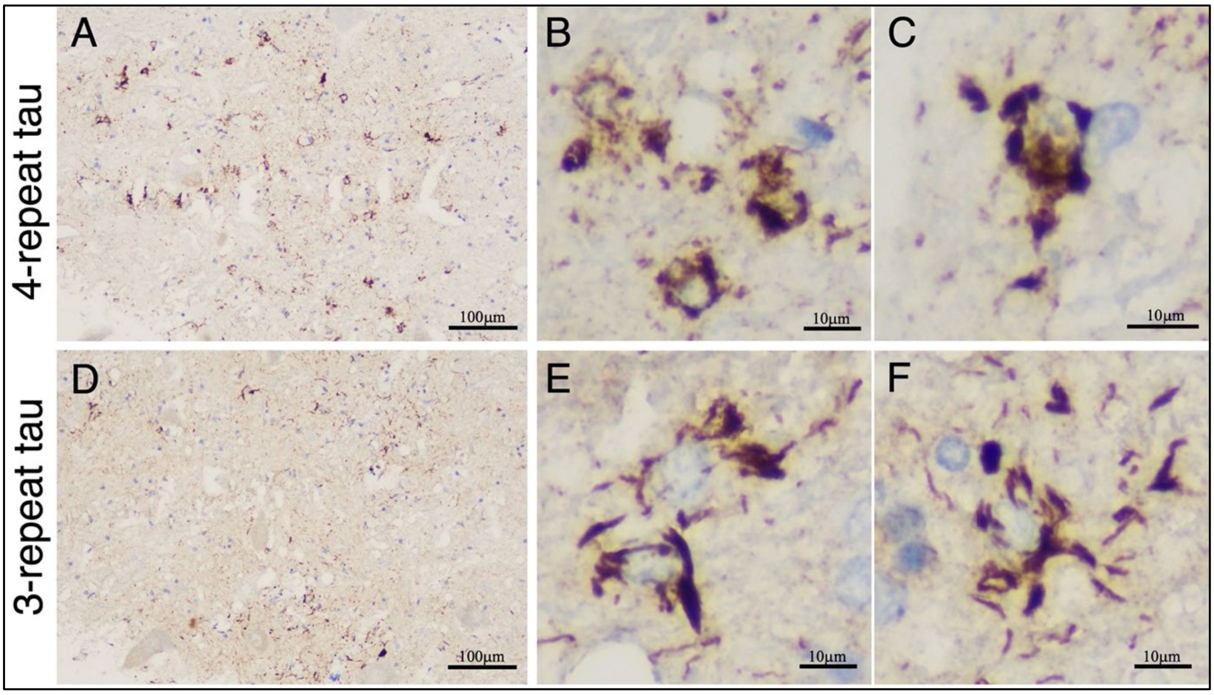

Tau isoform evaluation using serial sections (4R-tau: A–C; 3R-tau: D–F) revealed 4R-tau-positive and 3R-tau-positive astrocytes in the anterior horn (A, D: low magnification of same region). At high magnification, 3R- and 4R-tau deposition can be observed in structures corresponding to astrocytic processes (B, C: 4R-tau; E, F: 3R-tau).
(A-C) RD4, (D-F) RD3.

© 2026 Tanaka H et al. *JAMA Neurology*.

eFigure 9. Representative Photomicrographs of 3R-Tau and 4R-Tau-Positive Threads in the Spinal White Matter

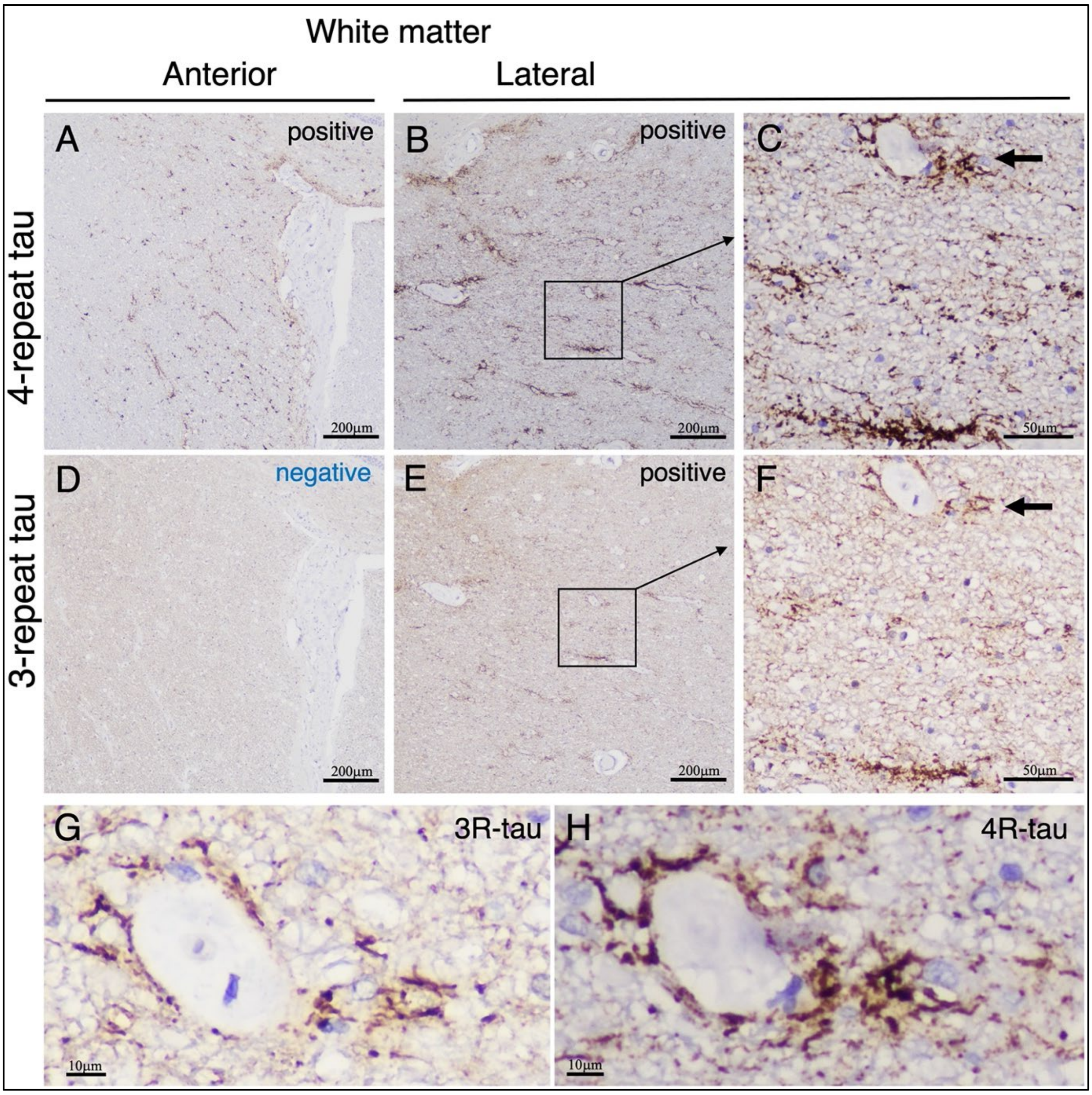

Tau isoform evaluation using serial sections (4R-tau: A–C and H; 3R-tau: D–F and G) reveals 4R-tau-positive but 3R-tau-negative structures in the anterior white matter (A, D). In addition to this lesion, abundant 4R-tau-positive (B, C) and 3R-tau-positive (E, F) threads are observed in the lateral white matter. These structures are accentuated around small blood vessels (G and H; corresponding to arrows in C and F).
(A-H) CTE#8, cervical cord. (A-C, H) RD4, (D-F, G) RD3.

© 2026 Tanaka H et al. *JAMA Neurology*.

eFigure 10. Representative p-Tau Immunoreactivity in the Spinal Cord of Aged Controls

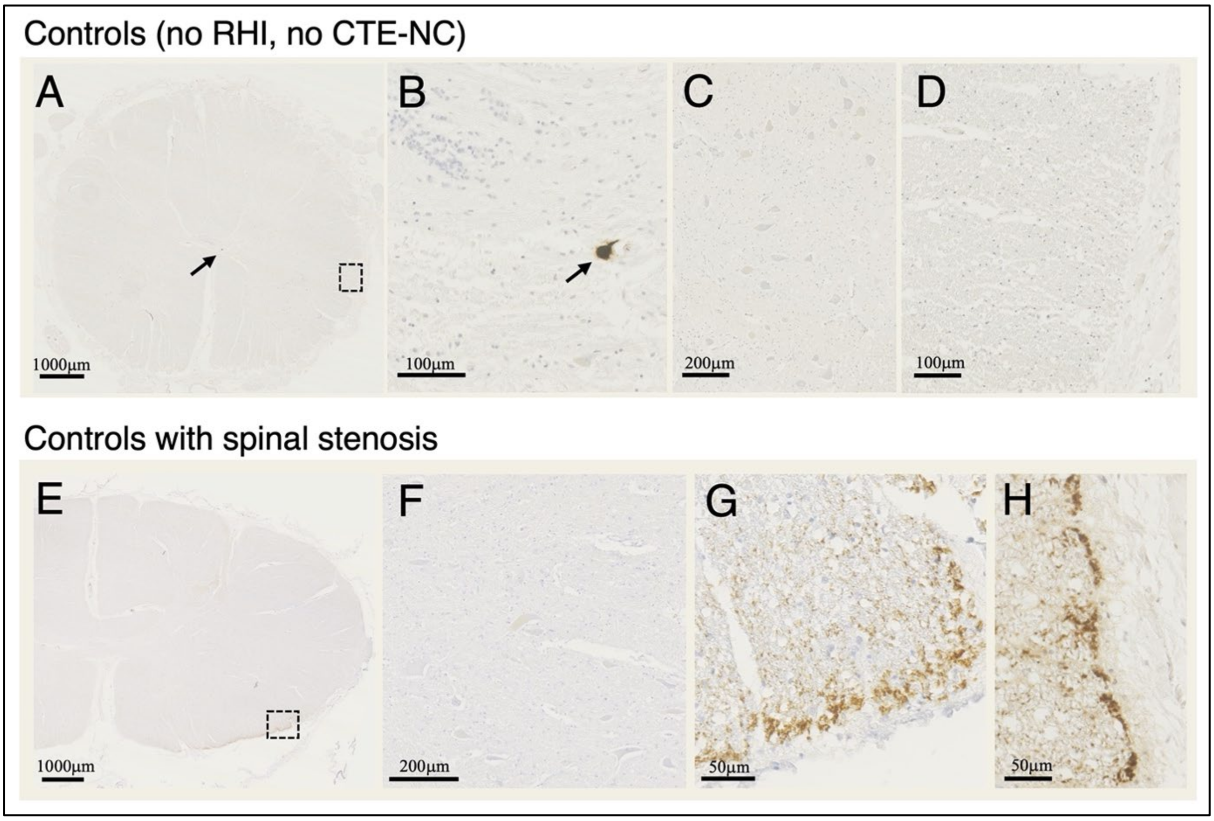

(A–D: Ctrl #3) In aged controls, only sparse p-tau deposits are observed (A: low-magnification view of a transverse section; B: high-magnification view of the gray matter. Black arrow: a single p-tau-positive neuron). (C) In another gray matter region, all anterior horn cells are p-tau negative. (D: boxed area in A) No p-tau deposits are seen in the white matter, including p-tau-positive astrocytes.
(E–H) In aged control cases with spinal stenosis, p-tau deposits are similarly sparse or absent in the gray matter (F). However, thread-like p-tau-positive structures in the subpial region (TSs-SP) are observed (E: low magnification; G: boxed area in E, Ctrl #1; H: Ctrl #4).
(A–H) PHF-1.

© 2026 Tanaka H et al. *JAMA Neurology*.

eFigure 11. Regional Comparison of Spinal Cord Tau Pathology in CTE-RHI Cases Aged ≥65 Years

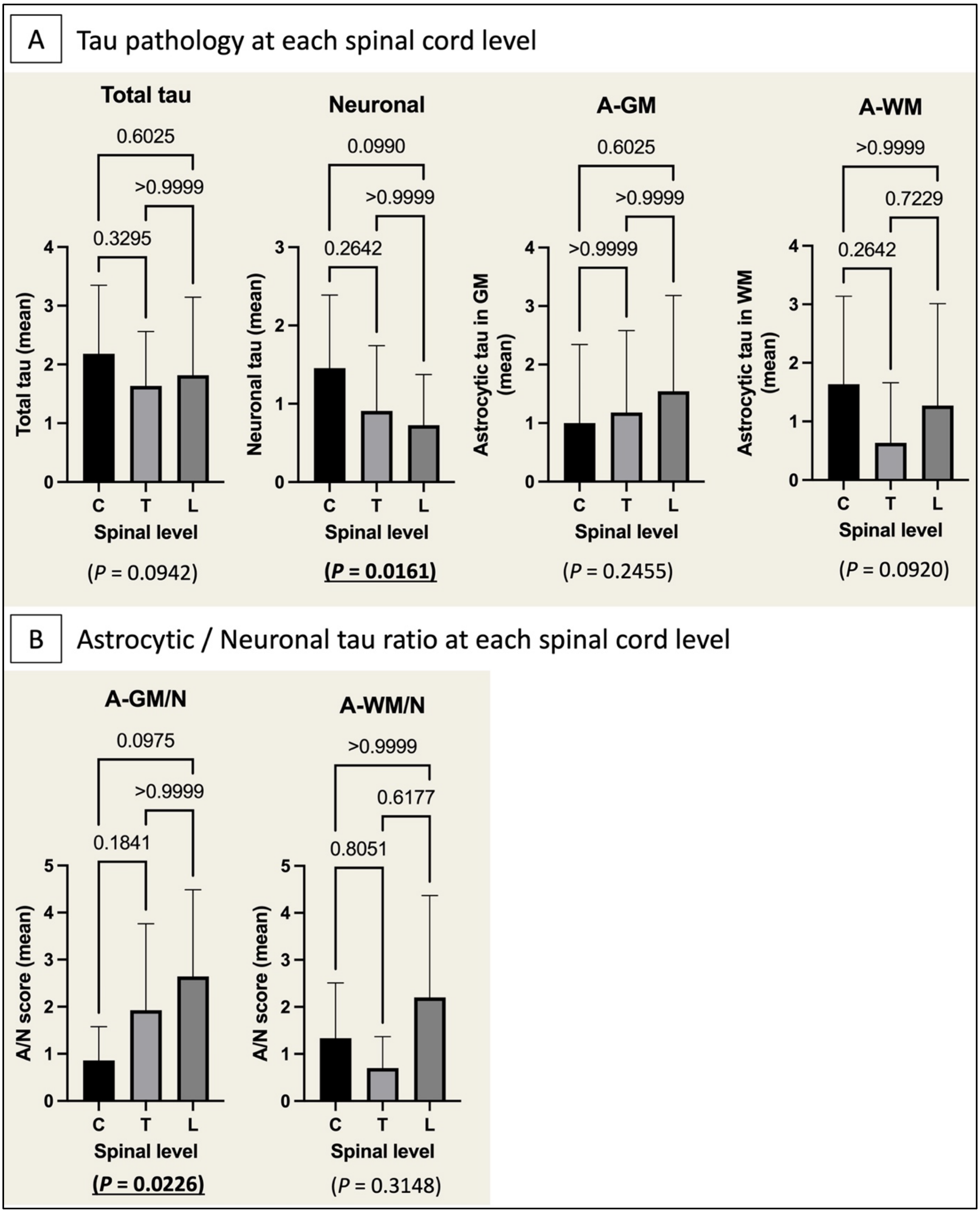

Abbreviations: Total tau, severity of total tau pathology; Neuronal (N), neuronal tau pathology; A-GM, astrocytic tau pathology in the gray matter; A-WM, astrocytic tau pathology in the white matter; A/N score, astrocytic tau score divided by neuronal tau score; C, cervical; T, thoracic; L, lumbar.
Statistical analysis: Friedman test. Post-hoc analysis using Dunn's test

© 2026 Tanaka H et al. *JAMA Neurology*.

eFigure 12. Representative p-TDP-43 Immunopositive Inclusions in CTE-NC Cases

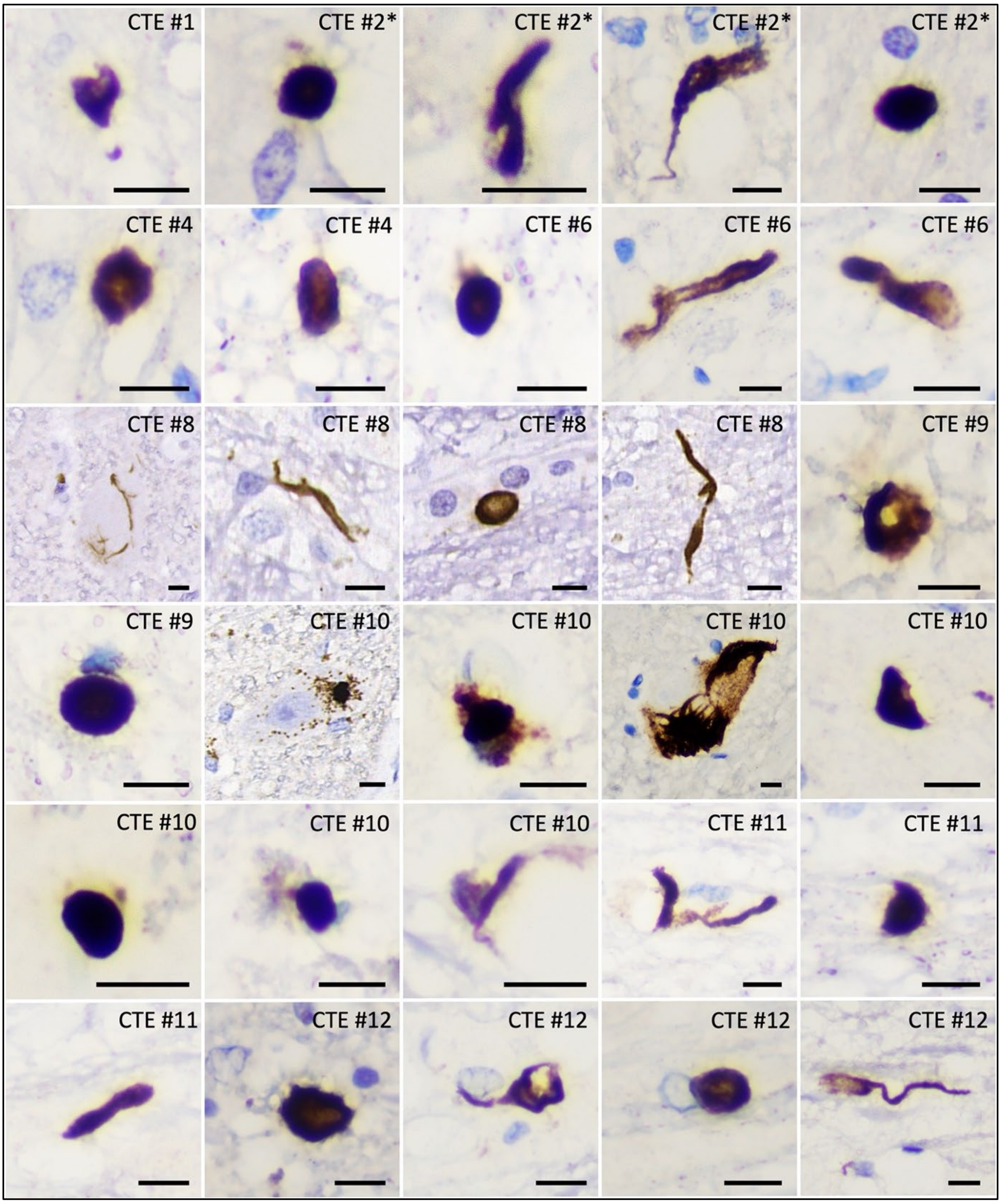

*Coexisting FTLD-TDP, type A was observed in CTE#2. All panels = p-TDP-43. All scale bars = 10 µm.

© 2026 Tanaka H et al. *JAMA Neurology*.

eFigure 13. Representative Photomicrographs of the Anterior Horn in CTE-NC Cases

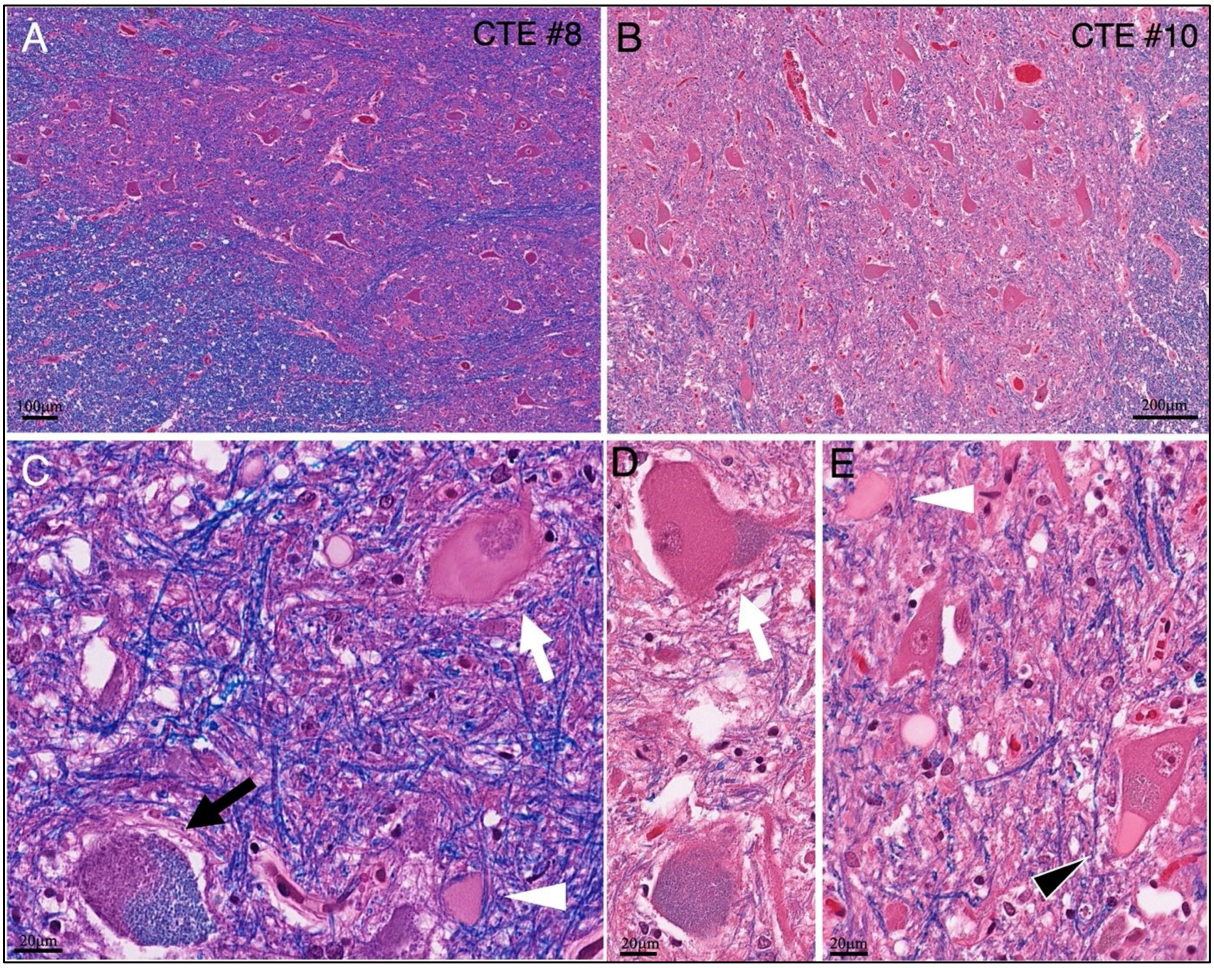

In CTE-NC cases aged ≥65 years from the confirmed RHI group, no remarkable neuronal loss is observed in the anterior horn (A, B; black arrow in C: healthy anterior horn cell/large motor neuron). However, large motor neurons showing central chromatolysis (white arrow) are frequently seen, and axonal spheroids (white arrowhead) are often observed in their vicinity, suggesting impaired axonal transport (C–E). In addition, hyalin-like inclusions, commonly seen in ALS, are also noted (E, black arrowhead).
(A, C) CTE #8; (B, D, E) CTE #10. (A–E) HE-LFB.

© 2026 Tanaka H et al. *JAMA Neurology*.

eFigure 14. Representative Spinal Cord Pathology in CTE-NC Cases From Indeterminate RHI Group

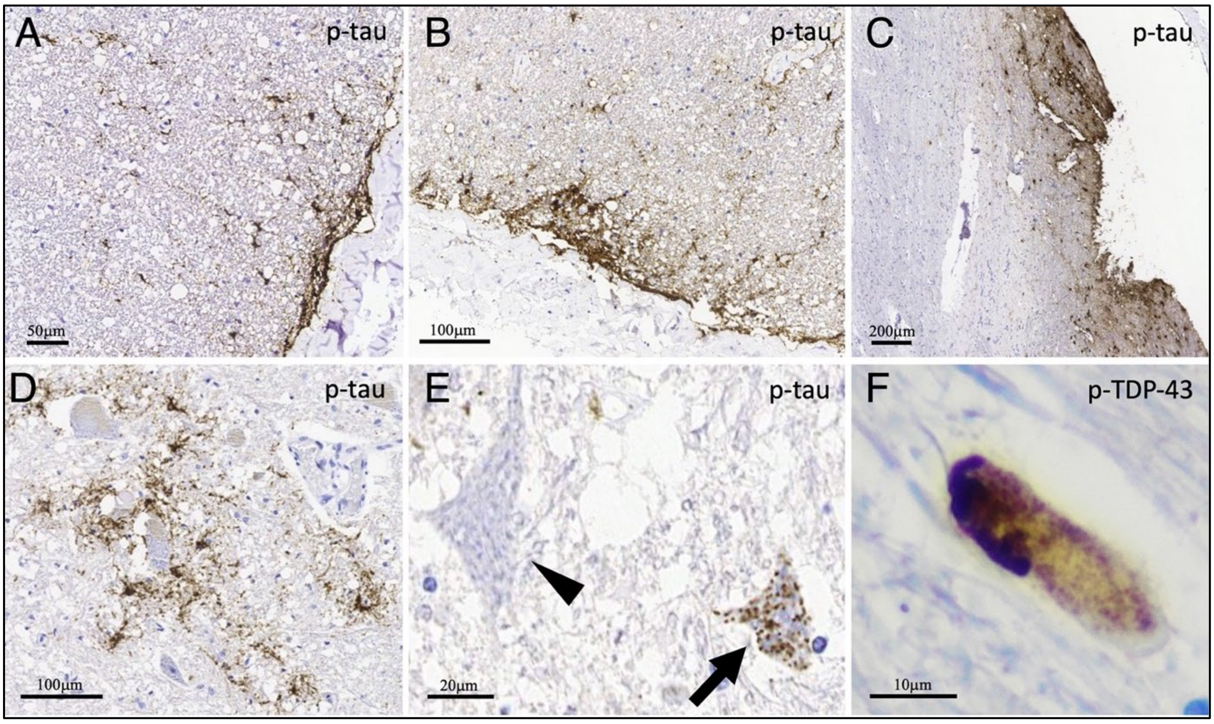

(A-C) Astrocytic tau pathology in the white matter (subpial regions). (D) astrocytic tau pathology in the gray matter. (E) p-Tau deposits in the large motor neuron (arrow: p-tau-positive anterior horn cell; arrowhead: p-tau-negative anterior horn cell). (F) p-TDP-43-positive inclusion in the neuron of the cervical cord (C-CTE#4).
(A-E): C-CTE#3 (A, C: cervical; B, E: Thoracic; D: Lumbar). (A-E) AT8, (F) p-TDP-43.

© 2026 Tanaka H et al. *JAMA Neurology*.

eFigure 15. Representative p-Tau Lesions in CTE-RHI Cases Aged <65 Years

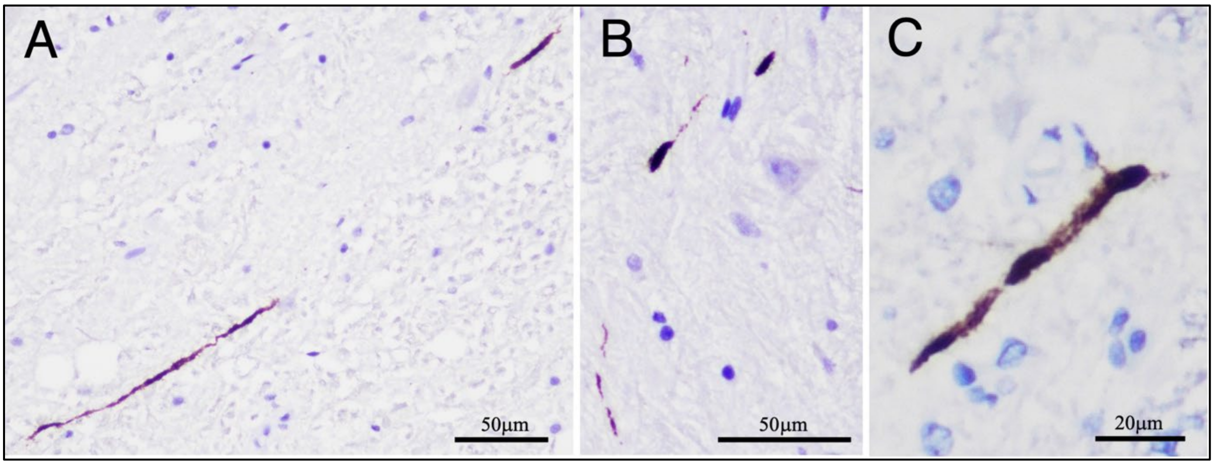

(A-C) Both CTE-NC cases showed p-tau deposits in the spinal cord (M-CTE#2: A, p-tau-positive long neurites in the lumbar cord; B, spherical neurites in the thoracic cord; M-CTE#1: C, spherical neurite in the cervical cord). (A-C) AT8.

© 2026 Tanaka H et al. *JAMA Neurology*.

eFigure 16. Representative APP Staining of the Spinal Cord in CTE-NC Cases

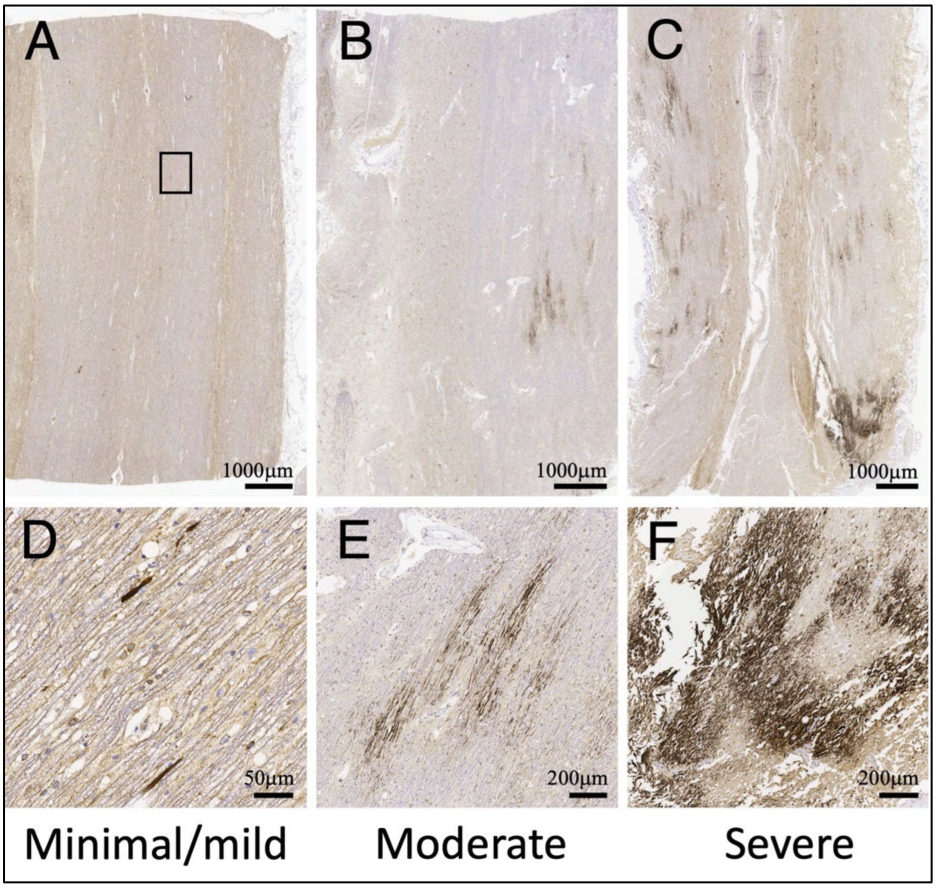

(A–F) Representative images of APP staining in the spinal cord of CTE-NC cases (coronal sections). A–C: low magnification; D–F: high magnification views of A–C. The severity of APP-positive axonal spheroids is classified into three degrees: minimal/mild (A, D: boxed area in A), moderate (B, E), and severe (C, F).

© 2026 Tanaka H et al. *JAMA Neurology*.

eFigure 17. Representative HLA-DR and APP Staining in Aged Control Cases

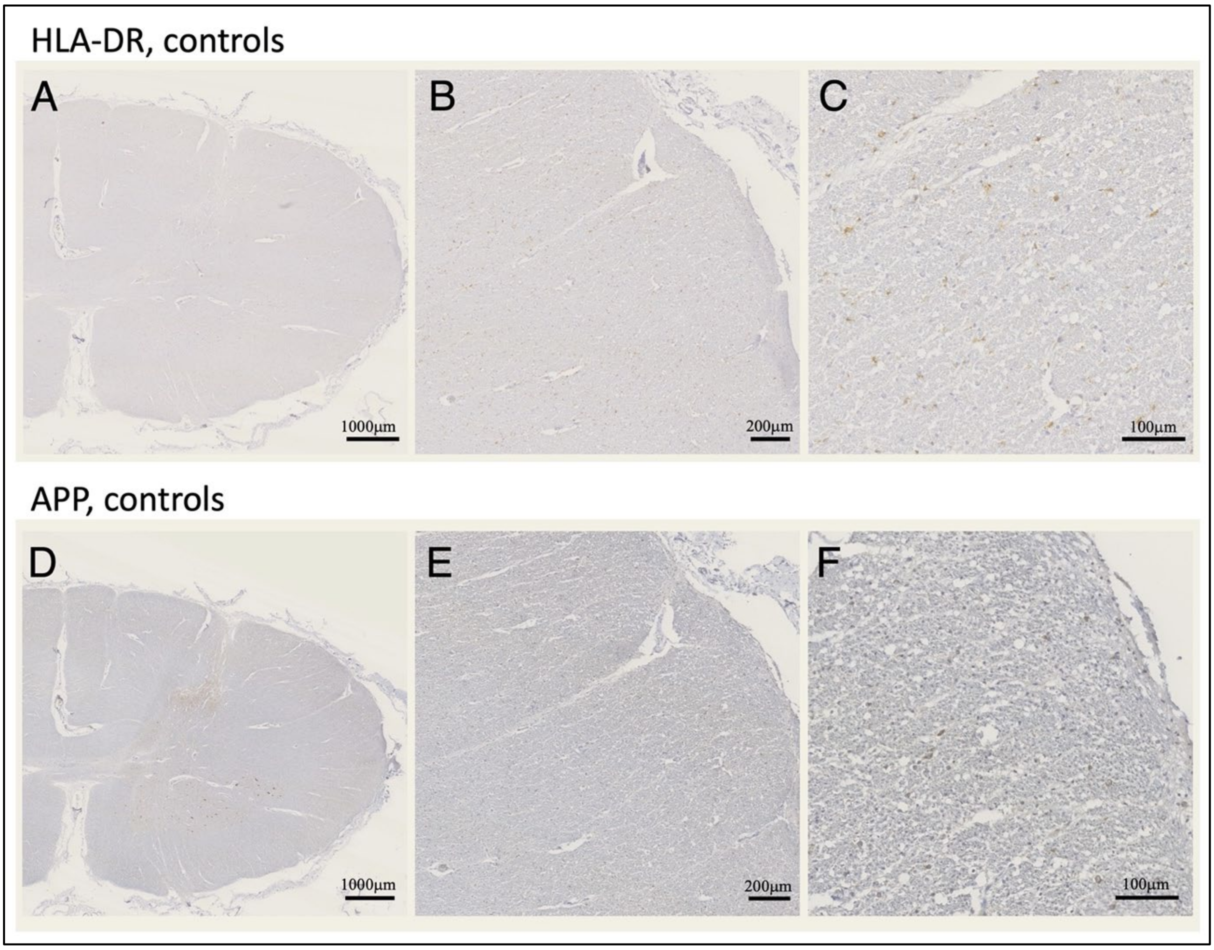

(A–C) HLA-DR staining of the spinal cord in aged control cases. (A) Low-magnification overview. (B) Corticospinal tract. (C) High-magnification view of B, showing only a few HLA-DR–positive microglia (Ctrl #1).
(D–F) APP staining of the spinal cord in aged control cases. (D) Low-magnification overview. (E) Corticospinal tract. (F) High-magnification view of E. APP-positive axonal spheroids are not observed in control case (Ctrl #1).

© 2026 Tanaka H et al. *JAMA Neurology*.

eFigure 18. Small Vessel Changes in the Spinal Cord in CTE-NC Cases

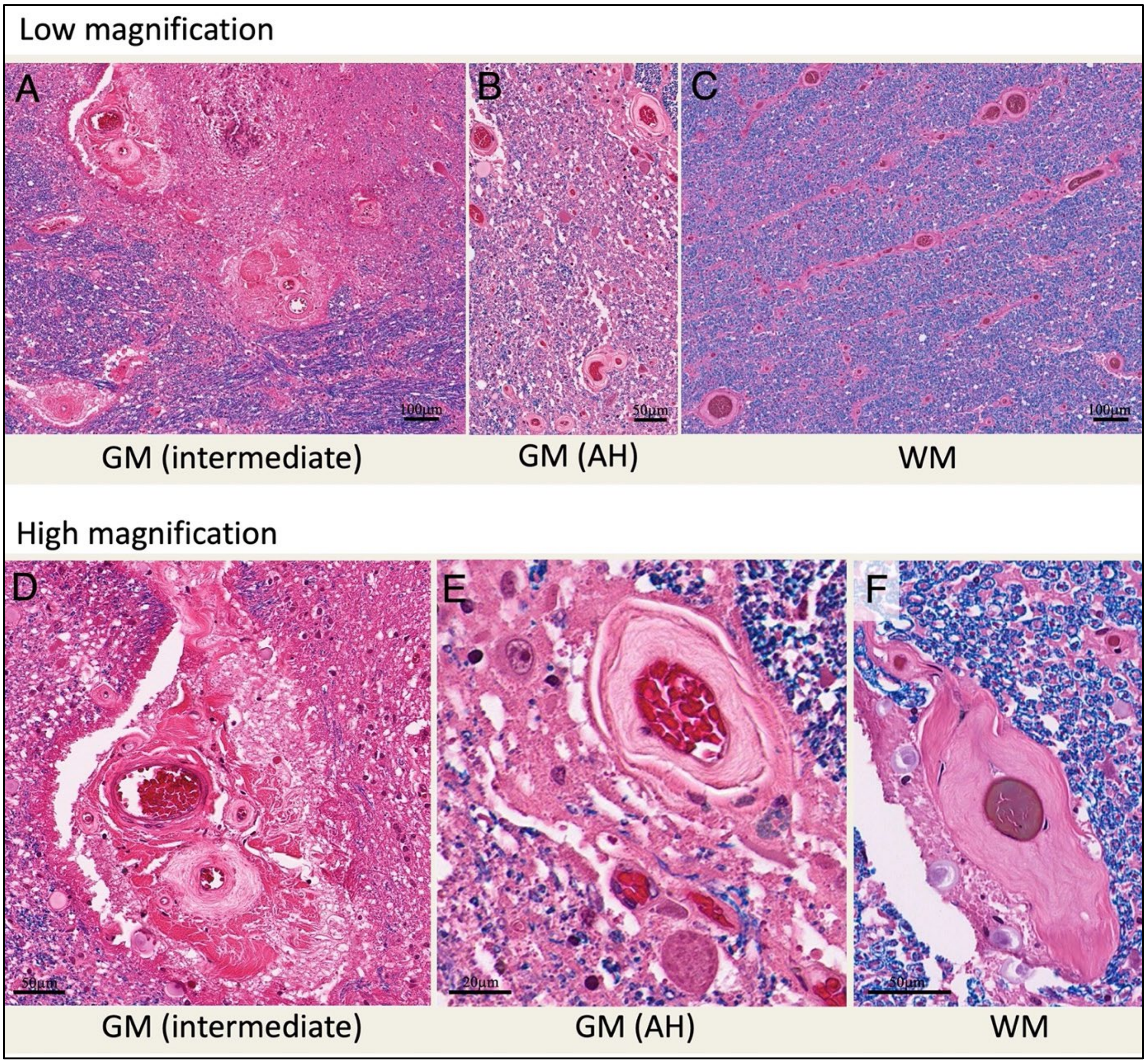

Small vessel changes characterized by hyalinization and thickened vessel walls in the spinal cord are observed in CTE-RHI cases aged ≥65 years (A, D: intermediate zone of the gray matter (GM); B, E: anterior horn (AH); C, F: white matter (WM)/ cortico-spinal tract).
(A-F) HE-LFB.

© 2026 Tanaka H et al. *JAMA Neurology*.

eFigure 19. Small Vessel Changes in the Spinal Cord in Controls

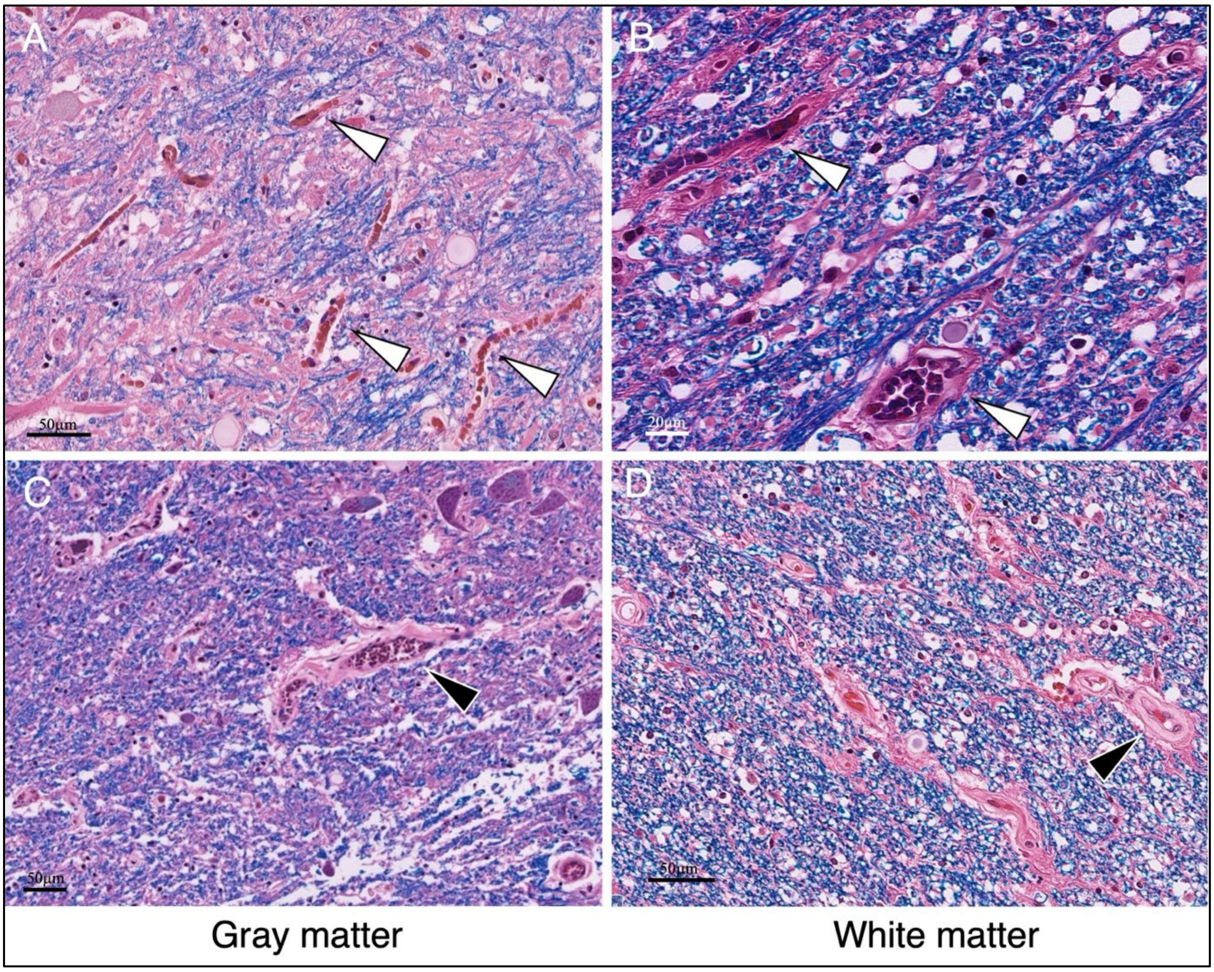

In controls, small vessel changes are usually none (A, B) or minimal to mild (C, D). White allow heads indicate small vessels without thickened wall. Black allow heads indicate small vessels with minimal to mild thickened wall.
(A, C) anterior horn, (B,D) white matter (WM)/ cortico-spinal tract.
(A) Ctrl #10, lumbar level (B) Ctrl #13, lumbar level (C) Ctrl #9, cervical level (D) Ctrl #10, cervical level.
(A-D) HE-LFB.

© 2026 Tanaka H et al. *JAMA Neurology*.

eFigure 20. Spinal Cord Pathology Correlation Heatmap in CTE-RHI Cases Aged ≥65 Years

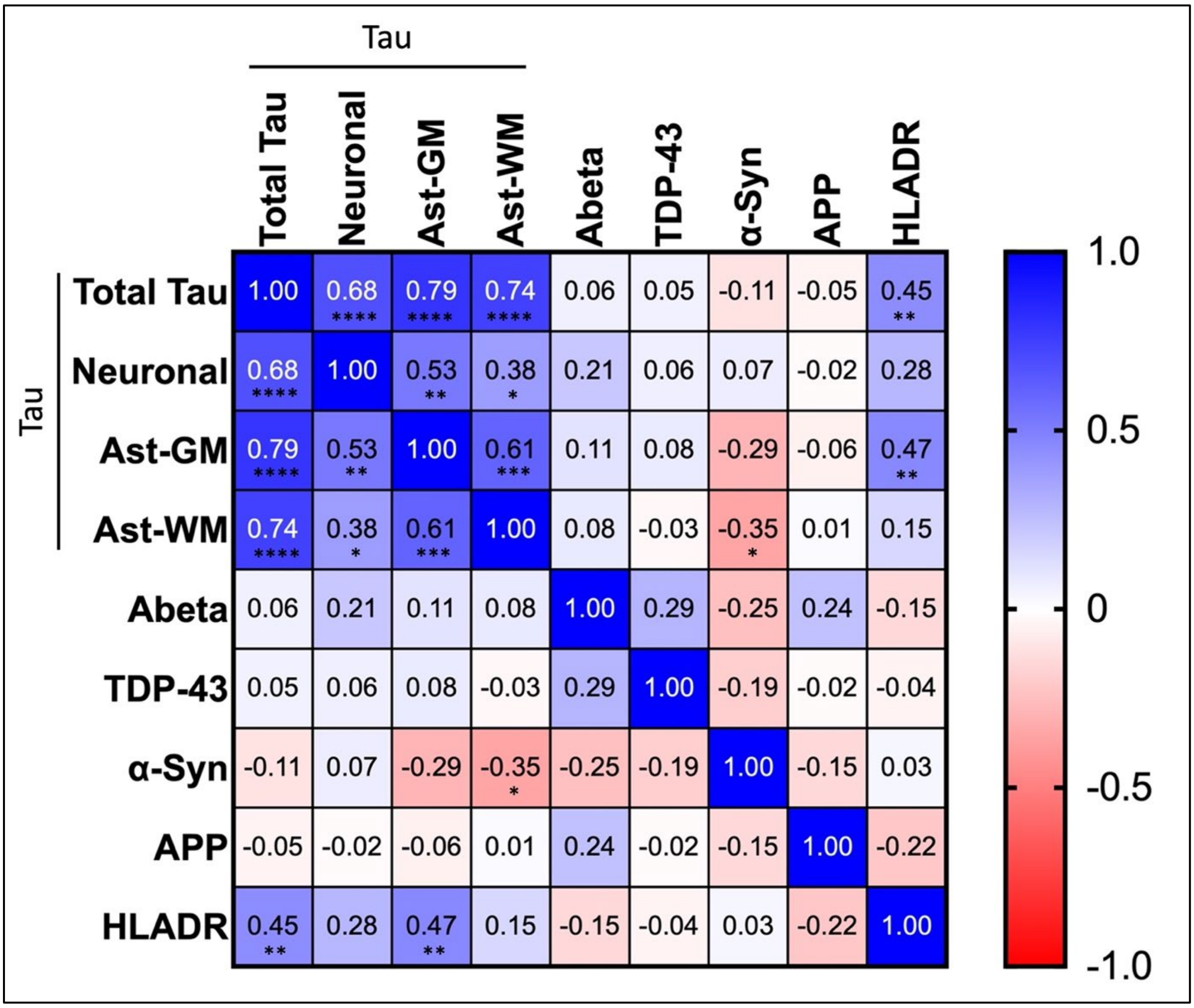

Abbreviations: Total tau, severity of total tau pathology; Neuronal, neuronal tau pathology; Ast-GM, astrocytic tau pathology in the gray matter; Ast-WM, astrocytic tau pathology in the white matter; TDP-43, TAR DNA-binding protein 43; α-Syn, α-synuclein; Abeta, amyloid-β; APP, APP-positive axonal spheroids; HLA-DR, HLA-DR-positive microglia.

Statistical analysis: two-tailed Spearman's rank correlation (*p < 0.05, **P < 0.01, ***P < 0.0001, ****P < 0.00001).

© 2026 Tanaka H et al. *JAMA Neurology*.

eTable 1. Summary of Clinicopathological Characteristics in Groups of RHI Cohort

RHI Subgroups	Confirmed RHI (n=23)	Indeterminate RHI (n=34)	Non-RHI (n=13)	*P* [e] (cRHI vs nonRHI)
**Clinical**				
**Age (mean, min-max, y)**	70.52 (20-85)	56.76 (41-67)	73.54 (59-84)	0.4579
**Sex (M:F)**	22:1	29:5	11:2	0.5394
**History of RHI**	All	Indeterminate	None	
**Spinal cord**				
**Tau - Total (frequency, %)** [a]	20/23 (87)	15/34 (44)	12/13 (92)	>0.9999
**(severity, mean)** [b]	1.33	0.3	0.74	**0.0327**
**- Neuron (frequency, %)**	18/23 (78)	9/34 (26)	6/13 (46)	0.0714
**(severity, mean)**	0.76	0.09	0.35	**0.0173**
**- Astrocyte**	14/23 (61)	4/34 (12)	0/13 (0)	**0.0003**
**- in GM**	12/23 (52)	4/34 (12)	0/13 (0)	**0.0022**
**- in WM**	10/23 (43)	2/34 (6)	0/13 (0)	**0.0058**
**TDP-43**	10/23 (43)	1/34 (3)	0/13 (0)	**0.0058**
**α-Syn (frequency, %)**	7/23 (30)	na	2/13 (15)	0.4378
**(severity, mean)**	0.46	na	0.23	0.3062
**Aβ (frequency, %)**	17/23 (74)	na	5/13 (38)	0.0732
**(severity, mean)**	1.25	na	0.35	**0.0001**
**SAA**	3/23 (13)	na	1/13 (8)	>0.9999
**Brain (Mixed pathology)**				
**AD-NC (High-Int., %)**	13/23 (57)	0/34 (0)	8/12 (67)	0.721
**(severity, mean)** [c]	1.65	0.26	2.17	0.2341
**Aβ (A) (frequency, %)**	17/23 (74)	9/34 (26)	11/12 (92)	0.3800
**(severity, mean)** [d]	1.83	0.26	2.25	0.3849
**CAA**	13/23 (57)	4/34 (12)	9/12 (75)	0.4630
**AGD**	8/23 (35)	4/34 (12)	0/12 (0)	**0.0316**
**LBD**	10/22 (45)	3/34 (9)	5/11 (45)	>0.9999
**LATE**	10/23 (43)	0/34 (0)	3/12 (25)	0.4630
**CTE-NC**	16/23 (70)	4/34 (12)	0/12 (0)	**<0.0001**
**- High:Low**	8:7 [f]	0:4	0:0	
**Other primary tauopathy** [g]	3/23 (13)	0/34 (0)	0/12 (0)	0.5361

Abbreviations: CTE-NC, cases with chronic traumatic encephalopathy neuropathologic change in the brain; AD-NC, Alzheimer's disease neuropathologic change[4]; y, years; M, male; F, female; RHI, repetitive head impacts; GM, gray matter; WM, white matter; TDP-43, TAR DNA-binding protein 43; α-Syn, α-synuclein; Aβ, amyloid-β; SAA, spinal amyloid angiopathy: amyloid angiopathy in the spinal cord vessels; CAA, cerebral amyloid angiopathy [5]; Aβ (A), A score of AD-NC[4]; AGD, argyrophilic grain disease[6]; LBD, Lewy body disease [7,9]; LATE, limbic predominant age-related TDP-43 encephalopathy[8]; Int., intermediate; na, not available.

[a] Frequencies were determined as the ratio of positive cases to the number of available cases.

© 2026 Tanaka H et al. *JAMA Neurology*.

[b] Severity was determined by averaging scores from all available cases across all spinal cord levels.
[c] AD-NC severity was determined by averaging the AD-NC scores, which were defined as follows: Not = 0; Low = 1; Intermediate = 2; High = 3.
[d] Aβ severity was calculated as the average of the A scores of AD-NC[4].
[e] P values were calculated by comparing the Confirmed RHI group (cRHI) with the Non-RHI group.
[f] CTE-NC level of single with CTE-NC could not be reliably assessed due to coexisting very severe Alzheimer's disease neuropathologic change.
[g] Other primary tauopathies include progressive supranuclear palsy (PSP), corticobasal degeneration (CBD), and globular glial tauopathy (GGT).

© 2026 Tanaka H et al. *JAMA Neurology*.

eTable 2. Summary of Antibodies Used in This Study

Antibody	Remarks (clone, aa target)	Source	Mono/Polyclonal	Dilution	1st Antigen retrieval	2nd Antigen retrieval
**p-Tau**	**AT8; pSer202/Thr205**	Invitrogen/ThermoFisher	Monoclonal	1:1000	Heat (low pH)*	None
p-Tau [a]	PHF-1; pSer396/Ser404	Dr. Peter Davies	Monoclonal	1:1000	Heat (Tris-EDTA, pH 8)	None
**4 repeat-tau**	**RD4**	Millipore/Sigma	Monoclonal	1:200	Heat (low pH)*	80% FA (1 min)
**3 repeat-tau**	**RD3**	Millipore/Sigma	Monoclonal	1:2000	Heat (low pH)*	80% FA (1 min)
p62	Clone 3/P62 LCK LIGAND	BD Transduction Laboratories	Monoclonal	1:800	Heat (low pH)*	None
**p-TDP-43**	**pSer409/410**	Cosmo Bio Co., LTD.	Monoclonal	1:2000	Heat (low pH)*	80% FA (1 min)
p-TDP-43 [a]	pSer409/410	BioLegend	Monoclonal	1:500	Heat (Tris-EDTA, pH 8)	None
**α-Synuclein**	**Clone 5G4**	Analytikjena	Monoclonal	1:4000	Heat (low pH)*	80% FA (5 mins)
α-Synuclein [a]	Clone KM51	Leica Biosystems	Monoclonal	1:200	98% FA (5 mins)	Heat (Tris-EDTA, pH 8)
**Beta-Amyloid**	**Clone 12B2**	IBL	Monoclonal	1:400	80% FA (1 hour)	None
Beta-Amyloid [a]	Clone BA4	Dako/Agilent	Monoclonal	1:75	98% FA (5 mins)	Heat (Tris-EDTA, pH 8)
Beta-Amyloid [a]	Clone 6E10	BioLegend	Monoclonal	1:5000	98% FA (5 mins)	Heat (Citrate, pH 6)
**APP**	**Clone 22C11**	Millipore/Sigma	Monoclonal	1:3000	Heat (low pH)*	None
APP [a]	Clone 22C11	Millipore/Sigma	Monoclonal	1:50,000	Heat (Tris-EDTA, pH 8)	None
HLA-DR [a]	Clone CR3/43	Dako	Monoclonal	1:800	Heat (Tris-EDTA, pH 8)	None
**HLA-DR**	**Clone CR3/43**	Abcam	Monoclonal	1:1000	Heat (low pH)*	None

Immunostaining was performed using the Dako Autostainer Link 48 and EnVision FLEX+ Visualization System, according to manufacturer's instructions. All sections were counterstained with hematoxylin. Abbreviation: p-, phosphorylated; FA, formic acid. Bold indicates that the major antibodies used for the detection of each protein in this study. [a]These antibodies were additionally used for manual staining of a subset of sections.
*Heat-mediated antigen retrieval was performed incubating the slides with low pH solution (citrate buffer pH6) during 20 minutes at 97ºC (Dako PT Link with low pH solution).

© 2026 Tanaka H et al. *JAMA Neurology*.

eTable 3. Comparison of CTE-RHI vs nonCTE-RHI Cases in Confirmed RHI Group

Confirmed RHI (n=23)	CTE-RHI (n=16)	nonCTE-RHI (n=7)	*P* [a]
**Clinical**			
**Age (mean, min-max, y)**	76.31 (44-85)	57.29 (20-81)	**0.0120**
**Sex (M:F)**	16:0	6:1	0.3043
**Duration**	17.31	20.25	0.8153
**Concussions score** [b]	2.56	2.29	0.5030
**Spinal cord**			
**Tau - Total (frequency, %)** [c]	16/16 (100)	4/7 (57)	**0.0198**
**(severity, mean)** [d]	1.71	0.57	**<0.0001**
**- Neuron (frequency, %)**	14/16 (88)	4/7 (57)	0.1421
**(severity, mean)**	0.93	0.43	**0.0263**
**- Astrocyte (frequency, %)**	12/16 (75)	2/7 (29)	0.0657
**- in GM (frequency, %)**	10/16 (63)	2/7 (29)	0.1930
**(severity, mean)**	1.10	0.14	**0.0015**
**- in WM (frequency, %)**	10/16 (63)	0/7 (0)	**0.0075**
**(severity, mean)**	1.07	0.00	**0.0005**
**TDP-43**	9/16 (56)	1/7 (14)	0.0886
**α-Syn**	7/16 (44)	0/7 (0)	0.0574
**Aβ**	15/16 (94)	2/7 (29)	**0.0034**
**SAA**	2/16 (13)	1/7 (14)	>0.9999
**Brain (Mixed pathology)**			
**ADNC (High-Int., %)**	11/16 (69)	2/7 (29)	0.1688
**Aβ (frequency)**	14/16 (88)	3/7 (43)	**0.0450**
**CAA**	11/16 (69)	2/7 (29)	0.1688
**AGD**	6/16 (38)	2/7 (29)	>0.9999
**LBD**	8/15 (53)	2/7 (29)	0.3808
**LATE**	8/16 (50)	2/7 (29)	0.4050
**Other tauopathy** [e]	2/16 (13)[f]	1/7 (14)[g]	>0.9999

Abbreviations: CTE, cases with chronic traumatic encephalopathy neuropathologic change in the brain; Ctrl nonRHI, control cases from the Non-RHI group; *P*, *P* value; Age, age at death; y, years; M, male; F, female; RHI, repetitive head impacts; GM, gray matter; WM, white matter; TDP-43, TAR DNA-binding protein 43; α-Syn, α-synuclein; Aβ, amyloid-β; SAA, spinal amyloid angiopathy: amyloid angiopathy in the spinal cord vessels; AD-NC, Alzheimer's disease neuropathologic change[4]; CAA, cerebral amyloid angiopathy[5]; Aβ (A), A score of AD-NC[4]; AGD, argyrophilic grain disease[6]; LBD, Lewy body disease[7,9]; LATE, limbic predominant age-related TDP-43 encephalopathy[8]; Int, intermediate.

[a] Items with statistically significant differences ($p < 0.05$) are indicated in bold.

[b] Concussion score: a semi-quantitative weighting system was applied as follows: (1+) = 1–3 episodes; (2+) = 4–9; (3+) = 10–20; (4+) >20; (5+) = multiple (exact number unknown).

[c] Frequencies were determined as the ratio of positive cases to the number of available cases.

[d] Severity was determined by averaging scores from all available cases across all spinal cord levels.

[e] Other primary tauopathies include progressive supranuclear palsy (PSP), corticobasal degeneration (CBD), and globular glial tauopathy (GGT).

[f] CBD or GGT coexisting with CTE-NC. [g] Coexisting PSP in a nonCTE-RHI case.

© 2026 Tanaka H et al. *JAMA Neurology*.

eTable 4A. Severity of Spinal Cord Pathology at Each Spinal Level in CTE-RHI Cases Aged ≥65

	level	Tau				Predominant type	Other misfolded proteins			Degeneration	
		Total	N	A-GM	A-WM		TDP-43	α-Syn	Aβ	APP	HLA-DR
**CTE #1**	**C**	2	1	2	3	A-WM	0	0	2	1	3
	**T**	3	1	4	1	A-GM	1	0	2	1	3
	**L**	2	1	2	3	A-WM	0	0	2	1	4
**CTE #2**	**C**	1	1	0	1		1	0	2	3	1
	**T**	1	0	0	1		1	0	3	3	1
	**L**	0	0	0	0		1	0	2	1	1
**CTE #3**	**C**	1	1	2	0		0	0	3	3	1
**CTE #4**	**C**	1	1	0	0		0	1	3	1	2
	**T**	1	1	0	0		1	2	2	1	2
	**L**	1	1	0	0		1	1	3	1	2
**CTE #5**	**C**	3	2	2	3	A-WM	0	2	1	2	1
**CTE #6**	**C**	1	2	0	0		1	1	3	2	2
	**T**	1	1	1	0		0	2	2	3	2
	**L**	1	0	1	0		1	1	2	1	2
**CTE #7**	**C**	2	1	0	0		0	3	2	3	3
	**T**	1	0	0	0		0	2	1	3	3
	**L**	1	0	1	1		0	1	1	3	3
**CTE #8**	**C**	4	2	2	4	A-WM	2	0	3	3	2
	**T**	3	2	2	3	A-WM	0	0	2	3	2
	**L**	4	1	3	5	A-WM	0	0	2	2	2
**CTE #9**	**C**	3	3	2	2	N	1	0	2	2	5
	**T**	2	2	2	0		1	0	3	2	5
	**L**	4	1	5	3	A-GM	1	0	2	1	4
**CTE #10**	**C**	3	1	1	3	A-WM	2	0	2	2	2
	**T**	1	0	1	0		1	0	3	3	2
	**L**	2	1	2	2		2	0	3	3	2
**CTE #11**	**C**	4	3	4	3	A-GM	0	1	2	1	3
	**T**	3	2	3	2	A-GM	1	2	0	1	3
	**L**	3	2	3	0	A-GM	1	1	1	1	3
**CTE #12**	**C**	2	1	0	0		0	2	1	2	2
	**T**	1	0	0	0		1	3	1	1	2
	**L**	1	1	0	0		1	2	1	1	2
**CTE #13**	**C**	3	2	1	3	A-WM	0	2	4	1	2
**CTE #14**	**C**	1	0	0	2		0	0	0	1	2
	**T**	1	1	0	0		0	0	0	2	2
	**L**	1	0	0	0		0	0	0	1	2
	**Mean**	1.92	1.08	1.28	1.25	Ast-type: 7/14 (50)	0.61	0.81	1.89	1.83	2.36
**Positive/available # of cases (%)**		14/14 (100)	14/14 (100)	10/14 (71)	10/14 (71)		9/14 (64)	7/14 (50)	13/14 (93)	14/14 (100)	14/14 (100)
				12/14 (86%)[a]							

Abbreviations: Total, severity of total tau pathology; N, neuronal tau pathology; A-GM, astrocytic tau pathology in the gray matter; A-WM, astrocytic tau pathology in the white matter; TDP-43, TAR DNA-binding protein 43; α-Syn, α-synuclein; Aβ, amyloid-β; APP, APP-positive axonal spheroids; HLA-DR, HLA-DR-positive microglia; Ast-type, tau astrogliopathy-predominant type; C, cervical; T, thoracic; L, lumbar.
Semiquantitative assessment of the spinal cord pathology: (Tau-Total, Aβ) 0 = absent, 1 = minimal, 2 = mild, 3 = moderate, 4 = severe; (TDP-43) 0 = absent, 1 = present, 2 = present with neuronal cytoplasmic inclusions; (α-Syn, APP) 0 = absent, 1 = minimal/mild, 2 = moderate, 3 = severe; (Tau-N, A-GM, A-WM, HLA-DR) 0 = absent, 1 = minimal, 2 = mild, 3 = moderate, 4 = severe, 5 = extremely severe.
[a] Frequency of cases exhibiting all types of tau-positive astrocytes.

© 2026 Tanaka H et al. *JAMA Neurology*.

eTable 4B. Summary of Spinal Cord Pathology by Case in CTE-RHI Cases Aged ≥65

	Tau	N	A-GM	A-WM	TDP-43	α-Syn	Aβ	APP	HLA-DR
**CTE #1**	3	1	4	3	1	0	2	1	4
**CTE #2**	1	1	0	1	1	0	3	3	1
**CTE #3**	1	1	2	0	0	0	3	3	1
**CTE #4**	1	1	0	0	1	2	3	1	2
**CTE #5**	3	2	2	3	0	2	1	2	1
**CTE #6**	1	2	1	0	1	2	3	3	2
**CTE #7**	2	1	1	1	0	3	2	3	3
**CTE #8**	4	2	3	5	2	0	3	3	2
**CTE #9**	4	3	5	3	1	0	3	2	5
**CTE #10**	3	1	2	3	2	0	3	3	2
**CTE #11**	4	3	4	3	1	2	2	1	3
**CTE #12**	2	1	0	0	1	3	1	2	2
**CTE #13**	3	2	1	3	0	2	4	1	2
**CTE #14**	1	1	0	2	0	0	0	2	2

Abbreviations: Tau, severity of total tau pathology; N, neuronal tau pathology; A-GM, astrocytic tau pathology in the gray matter; A-WM, astrocytic tau pathology in the white matter; TDP-43, TAR DNA-binding protein 43; α-Syn, α-synuclein; Aβ, amyloid-β; APP, APP-positive axonal spheroids; HLA-DR, HLA-DR-positive microglia.
The severity score for each case was determined based on the highest score among the spinal cord levels.
Semiquantitative assessment of the spinal cord pathology: (Tau: total tau, Aβ) 0 = absent, 1 = minimal, 2 = mild, 3 = moderate, 4 = severe; (TDP-43) 0 = absent, 1 = present, 2 = present with neuronal cytoplasmic inclusions; (α-Syn, APP) 0 = absent, 1 = minimal/mild, 2 = moderate, 3 = severe; (Tau-N, A-GM, A-WM, HLA-DR) 0 = absent, 1 = minimal, 2 = mild, 3 = moderate, 4 = severe, 5 = extremely severe.

© 2026 Tanaka H et al. *JAMA Neurology*.

eTable 5. Summary of Major Clinicopathological Findings in Control Group (nonCTE-nonRHI)

General features					Pathology in the Spinal cord							Mixed pathology in the Brain					
Case #	Age	Sex	Spinal stenosis		Tau	Tau-N	Tau-Ast	TDP-43	α-Syn	Aβ	SAA	ADNC	Aβ(A)	CAA	AGD	LBD	LATE
**Ctrl #1**	M70s	M	+		+	-	-	-	-	-	-	Low	1	Type 2	-	-	-
**Ctrl #2**	M70s	M	+		+	+	-	-	-	-	-	na	na	na	na	na	na
**Ctrl #3**	M80s	F	-		+	+	-	-	-	-	-	Int	2	Type 1	-	-	-
**Ctrl #4**	M70s	M	+		++	-	-	-	-	-	-	Low	2	-	-	na	-
**Ctrl #5**	E80s	M	-		+	+	-	-	-	-	-	Low	1	-	-	-	-
**Ctrl #6**	L60s	M	-		-	-	-	-	-	-	-	Not	0	-	-	-	-
**Ctrl #7**	M70s	F	+		+	+	-	-	-	-	-	High	3	Type 1	-	-	-
**Ctrl #8**	L70s	M	-		+	-	-	-	-	+	-	High	3	Type 2	-	-	-
**Ctrl #9**	M70s	M	-		+	-	-	-	-	+	-	High	3	Type 2	-	Amyg	2
**Ctrl #10**	E70s	M	-		+	++	-	-	++	++	Type 2	High	3	Type 1	-	5	2
**Ctrl #11**	M70s	M	-		+	-	-	-	+	+	-	High	3	Type 2	-	4	-
**11 Ctrl aged ≧65**	Mean	M:F	4 cases	Frequency	10/11	5/11	0/11	0/11	2/11	4/11	1/11	High-Int:	9/10	7/10	0/10	3/9	2/10
	75.82	9:2		(%)[a]	(91)	(45)	(0)	(0)	(18)	(36)	(9)	6/10 (60)	(90)	(70)	(0)	(33)	(20)
**Ctrl #12**	E60s	M	-		+	+	-	-	-	-	-	High	3	Type 2	-	Amyg	2
**Ctrl #13**	L50s	M	-		+	-	-	-	-	+	-	High	3	Type 2	-	Amyg	-
**All 13 Ctrl**	Mean	M:F	4 cases	Frequency	12/13	6/13	0/13	0/13	2/13	5/13	1/13	High-Int:	11/12	9/12	0/12	5/11	3/12
	73.54	11:2		(%)[a]	(92)	(46)	(0)	(0)	(15)	(38)	(8)	8/12 (67)	(92)	(75)	(0)	(45)	(25)

Abbreviations: Age, age at death; E, early; M, mid; L, late (e.g., E70s = early 70s [70-73], M70s = mid-70s [74-76], L70s = late 70s [77-79]); F, female; M, male; Tau, severity of total tau pathology; Tau-N, neuronal tau pathology; Tau-Ast, astrocytic tau pathology; TDP-43, TAR DNA-binding protein 43; α-Syn, α-synuclein; Aβ, amyloid-β; SAA, spinal amyloid angiopathy: amyloid angiopathy in the spinal cord vessels; AD-NC, Alzheimer's disease neuropathologic change[4]; CAA, cerebral amyloid angiopathy [5]; Aβ (A), A score of AD-NC[4]; AGD, argyrophilic grain disease[6]; LBD, Lewy body disease [7,9]; LATE, limbic predominant age-related TDP-43 encephalopathy[8]; Int, intermediate; na, not available; Amyg, amygdala predominant. Semiquantitative assessment of the spinal cord pathology: (Tau: total, Tau-N, Tau-Ast, Aβ) - = absent, + = minimal, ++ = mild, +++ = moderate, ++++ = severe; (TDP-43) - = absent, + = present, ++ = present with neuronal cytoplasmic inclusions; (α-Syn) - = absent, + = minimal/mild, ++ = moderate, +++ = severe.

[a] Frequencies were determined as the ratio of positive cases to the number of available cases.

© 2026 Tanaka H et al. *JAMA Neurology*.

eTable 6. Tau Pathology of Spinal Cord at Each Level in Control Group (nonCTE-nonRHI)

Case #	Tau[a]	level	Total	N	A-GM	A-WM	TSs-SP
**Ctrl #1**	+	C	1	0	0	0	1
		T	0	0	0	0	0
		L/S	0	0	0	0	0
**Ctrl #2**	+	C	1	1	0	0	0
**Ctrl #3**	+	C	1	1	0	0	0
		L/S	1	1	0	0	0
**Ctrl #4**	++	C	2	0	0	0	2
**Ctrl #5**	+	C	1	1	0	0	1
		T	0	0	0	0	0
		L/S	1	1	0	0	0
**Ctrl #6**	-	C	0	0	0	0	0
		T	0	0	0	0	0
		L	0	0	0	0	0
**Ctrl #7**	+	C	1	1	0	0	0
		T	1*	0	0	0	0
		L	0	0	0	0	0
**Ctrl #8**	+	C	1*	0	0	0	0
		T	0	0	0	0	0
**Ctrl #9**	+	C	1*	0	0	0	0
		T	0	0	0	0	0
		L	1*	0	0	0	0
**Ctrl #10**	+	C	1	2	0	0	0
		T	1	1	0	0	0
		L	1	1	0	0	0
**Ctrl #11**	+	C	1*	0	0	0	0
		T	1*	0	0	0	0
		L	1*	0	0	0	0
**Summary of 11 Ctrl aged ≧65**		Mean	0.70	0.37	0.00	0.00	0.15
	Frequency (%) [b]		10/11 (91)	5/11 (45)	0/11 (0)	0/11 (0)	3/11 (27)
**Ctrl #12**	+	C	1	1	0	0	0
**Ctrl #13**	+	C	1*	0	0	0	0
		T	1*	0	0	0	0
		L	1*	0	0	0	0
**Summary of all 13 Ctrl**		Mean	0.74	0.35	0.00	0.00	0.13
	Frequency (%) [b]		12/13 (92)	6/13 (46)	0/13 (0)	0/13 (0)	3/13 (23)

Abbreviations: Tau, tau severity score for individual cases; Total, severity of total tau pathology; N, neuronal tau pathology; A-GM, astrocytic tau pathology in the gray matter; A-WM, astrocytic tau pathology in the white matter; TSs-SP, Thread-like tau-positive structures in subpial regions; C, cervical; T, thoracic; L, lumbar; S, sacral. Semiquantitative assessment of the spinal cord pathology: (Tau-Total, TSs-SP) 0 = absent, 1 = minimal, 2 = mild, 3 = moderate, 4 = severe; (Tau-N, A-GM, A-WM) 0 = absent, 1 = minimal, 2 = mild, 3 = moderate, 4 = severe, 5 = extremely severe.

[a] Tau severity score for each case was determined based on the highest score of "Total tau" among the spinal cord levels. - = absent, + = minimal, ++ = mild, +++ = moderate, ++++ = severe. [b] Frequencies were determined as the ratio of positive cases to the number of available cases.

*, only tau-positive neurites/threads are present.

© 2026 Tanaka H et al. *JAMA Neurology*.

eTable 7. Subgroup Comparison in CTE-NC Cases

CTE-NC (n=20)	Confirmed RHI				Indeterminate RHI	
	CTE-RHI (n=16)	≥65 years (n=14)	<65 years (n=2)	*P* [a]	CTE-NC (n=4)	*P* [b]
**Clinical**						
**Age (mean, min-max, y)**	76.31 (44-85)	80.50 (68-85)	47.00 (44-50)	**0.0083**	60.75 (56-67)	**0.0215**
**Sex (M:F)**	16:0	14:0	2:0	>0.9999	4:0	>0.9999
**Spinal cord**						
**Tau - Total (frequency, %)** [c]	16/16 (100)	14/14 (100)	2/2 (100)	>0.9999	4/4 (100)	>0.9999
**(severity, mean)** [d]	1.71	1.92	0.50	**0.0017**	1.08	0.1263
**- Neuron (frequency, %)**	14/16 (88)	14/14 (100)	0/2 (0)	**0.0083**	4/4 (100)	>0.9999
**(severity, mean)** [d]	0.93	1.08	0.00	**0.0016**	0.33	**0.0309**
**- Astrocyte**	12/16 (75)	12/14 (86)	0/2 (0)	0.0500	3/4 (75)	>0.9999
**- in GM**	10/16 (63)	10/14 (71)	0/2 (0)	0.1250	3/4 (75)	>0.9999
**- in WM**	10/16 (63)	10/14 (71)	0/2 (0)	0.1250	2/4 (50)	>0.9999
**TDP-43**	9/16 (56)	9/14 (64)	0/2 (0)	0.1750	1/4 (25)	0.5820
**α-Syn**	7/16 (44)	7/14 (50)	0/2 (0)	0.4750	0/4 (0)	0.2487
**Aβ**	15/16 (94)	13/14 (93)	2/2 (100)	>0.9999	4/4 (100)	>0.9999
**SAA**	2/16 (13)	2/14 (14)	0/2 (0)	>0.9999	0/4 (0)	>0.9999
**Brain (Mixed pathology)**						
**ADNC (High-Int., %)**	11/16 (69)	11/14 (79)	0/2 (0)	0.0833	0/4 (0)	**0.0260**
**(severity, mean)** [e]	2.06	2.21	1.00	0.0833	0.25	**0.0093**
**Aβ(A) (frequency)**	14/16 (88)	12/14 (86)	2/2 (100)	>0.9999	1/4 (25)	**0.0320**

© 2026 Tanaka H et al. *JAMA Neurology*.

(severity, mean)[f]	2.25	2.43	1.00	0.0833	0.25	**0.0072**
**CAA**	11/16 (69)	11/14 (79)	0/2 (0)	0.0833	0/4 (0)	**0.0260**
**AGD**	6/16 (38)	5/14 (36)	1/2 (50)	>0.9999	0/4 (0)	0.2675
**LBD**	8/15 (53)	8/13 (62)	0/2 (0)	0.2000	0/4 (0)	0.1032
**LATE**	8/16 (50)	8/14 (57)	0/2 (0)	0.4667	0/4 (0)	0.1166

Abbreviations: CTE-NC, cases with chronic traumatic encephalopathy neuropathologic change in the brain; RHI, repetitive head impacts; *P*, *P* value; Age, age at death; y, years; M, male; F, female; GM, gray matter; WM, white matter; TDP-43, TAR DNA-binding protein 43; α-Syn, α-synuclein; Aβ, amyloid-β; SAA, spinal amyloid angiopathy: amyloid angiopathy in the spinal cord vessels; AD-NC, Alzheimer's disease neuropathologic change[4]; CAA, cerebral amyloid angiopathy[5]; Aβ (A), A score of AD-NC[4]; AGD, argyrophilic grain disease[6]; LBD, Lewy body disease[7,9]; LATE, limbic predominant age-related TDP-43 encephalopathy[8]; Int, intermediate.

[a] Statistical analysis was performed to compare CTE-NC cases aged ≥65 and <65 years in the Confirmed RHI group. Items with statistically significant differences ($p < 0.05$) are indicated in bold.

[b] Statistical analysis was performed to compare CTE-NC cases from the Confirmed and Indeterminate RHI groups. Items with statistically significant differences ($p < 0.05$) are indicated in bold.

[c] Frequencies were determined as the ratio of positive cases to the number of available cases.

[d] Severity was determined by averaging scores from all available cases across all spinal cord levels.

[e] AD-NC severity was determined by averaging the AD-NC scores, which were defined as follows: Not = 0; Low = 1; Intermediate = 2; High = 3.

[f] Aβ severity was calculated as the average of the A scores of AD-NC[4].

© 2026 Tanaka H et al. *JAMA Neurology*.

eTable 8. Summary of Major Clinicopathological Findings in CTE-NC Cases From Indeterminate RHI Group

Clinical features				Spinal cord								Brain (mixed pathology)						
**Case #**	**Age**	**Sex**		**Tau**	**Tau-N**	**Tau-Ast (GM, WM)**	**Predominant-type**	**TDP-43**	**α-Syn**	**Aβ**	**SAA**	**ADNC**	**Aβ (A)**	**CAA**	**AGD**	**LBD**	**LATE**	**CTE level**
**C-CTE #1**	L60s	M		+	(+)	(-)		-	-	+	-	Not	0	-	-	-	-	Low
**C-CTE #2**	M50s	M		+	(+)	(+, -)		-	-	++	-	Not	0	-	-	-	-	Low
**C-CTE #3**	E60s	M		++	(+)	(+, +)	A-WM	-	-	++	-	Low	1	-	-	-	-	Low
**C-CTE #4**	L50s	M		+	(+)	(+, +)		++	-	+	-	Not	0	-	-	-	-	Low
Mean	60.75	M:F	Positive / available # of cases (%)	4/4	4/4	3/4 (3, 2)	Ast-type: 1/4	1/4	0/4	4/4	0/4	High-Int: 0	1/4	0/4	0/4	0/4	0/4	Low:4
		4 : 0		(100)	(100)	(75)	(25)	(25)	(0)	(100)	(0)	(0)	(25)	(0)	(0)	(0)	(0)	High:0

Abbreviations: Age, age at death; E, early; M, mid; L, late (e.g., E70s = early 70s [70-73], M70s = mid-70s [74-76], L70s = late 70s [77-79]); M, male; Tau, severity of total tau pathology; Tau-N, neuronal tau pathology; Tau-Ast, astrocytic tau pathology; GM, gray matter; WM, white matter; TDP-43, TAR DNA-binding protein 43; α-Syn, α-synuclein; Aβ, amyloid-β; SAA, spinal amyloid angiopathy: amyloid angiopathy in the spinal cord vessels; AD-NC, Alzheimer's disease neuropathologic change[4]; CAA, cerebral amyloid angiopathy [5]; Aβ (A), A score of AD-NC[4]; AGD, argyrophilic grain disease[6]; LBD, Lewy body disease [7,9]; LATE, limbic predominant age-related TDP-43 encephalopathy[8]; CTE-level, the level of severity of chronic traumatic encephalopathy neuropathologic change [2]; Ast-type, tau astrogliopathy-predominant type; Int, intermediate.
Semiquantitative assessment of the spinal cord pathology: (Tau: total, Aβ) - = absent, + = minimal, ++ = mild, +++ = moderate, ++++ = severe; (TDP-43) - = absent, + = present, ++ = present with neuronal cytoplasmic inclusions; (α-Syn) - = absent, + = minimal/mild, ++ = moderate, +++ = severe; (Tau-N, Tau-Ast) - = absent, + = present.

© 2026 Tanaka H et al. *JAMA Neurology*.

eTable 9. Tau Pathology of Spinal Cord at Each Level in CTE-NC Cases From Indeterminate RHI Group

	Tau[a]	level	Total	N	A-GM	A-WM	Predominant-type
**C-CTE #1**	+	C	1*	0	0	0	
		T	0	0	0	0	
		L	1	1	0	0	
**C-CTE #2**	+	C	1	0	1	0	
		T	1	1	0	0	
		L	1*	0	0	0	
**C-CTE #3**	++	C	2	0	2	3	A-WM
		T	2	1	0	2	
		L	2	0	2	0	
**C-CTE #4**	+	C	1	1	1	2	
		T	0	0	0	0	
		L	1	0	1	0	
		Mean	1.08	0.33	0.58	0.58	
	Positive / available # of cases		4/4 (100)	4/4 (100)	3/4 (75)	2/4 (50)	
					3/4 (75)[b]		

Abbreviations: Tau, tau severity score for individual cases; Total, severity of total tau pathology; N, neuronal tau pathology; A-GM, astrocytic tau pathology in the gray matter; A-WM, astrocytic tau pathology in the white matter; C, cervical; T, thoracic; L, lumbar.
Semiquantitative assessment of the spinal cord pathology: (Total-tau) 0 = absent, 1 = minimal, 2 = mild, 3 = moderate, 4 = severe; (Tau-N, A-GM, A-WM) 0 = absent, 1 = minimal, 2 = mild, 3 = moderate, 4 = severe, 5 = extremely severe.
[a] Tau severity score for each case was determined based on the highest score of “Total tau” among the spinal cord levels. - = absent, + = minimal, ++ = mild, +++ = moderate, ++++ = severe.
[b] Frequency of cases exhibiting all types of tau-positive astrocytes.
*, only tau-positive neurites/threads are present.

© 2026 Tanaka H et al. *JAMA Neurology*.

eTable 10. Summary of Major Clinicopathological Findings in Non-CTE-NC Cases From Indeterminate RHI Group

Clinical features			Spinal cord				Brain (mixed pathology)					
Case #	Age	Sex	Tau	Tau-N	Tau-Ast (GM, WM)	TDP-43	AD-NC	Aβ(A)	CAA	AGD	LBD	LATE
**C-nonCTE #1**	E40s	M	-	-	-	-	Not	0	-	-	-	-
**C-nonCTE #2**	E60s	M	-	-	-	-	Low	1	-	-	-	-
**C-nonCTE #3***	M60s	M	+	+	-	-	Not	0	-	-	-	-
**C-nonCTE #4**	E60s	F	-	-	-	-	Low	1	+	+	-	-
**C-nonCTE #5**	E60s	M	-	-	-	-	Not	0	-	-	2	-
**C-nonCTE #6**	M50s	M	-	-	-	-	Not	0	-	-	4	-
**C-nonCTE #7***	M50s	M	+	+	-	-	Not	0	-	-	-	-
**C-nonCTE #8**	L40s	M	-	-	-	-	Low	1	-	-	-	-
**C-nonCTE #9**	M50s	F	-	-	-	-	Not	0	-	-	-	-
**C-nonCTE #10**	L50s	M	-	-	-	-	Not	0	-	-	4	-
**C-nonCTE #11**	L50s	M	-	-	-	-	Not	0	-	-	-	-
**C-nonCTE #12**	E50s	M	-	-	-	-	Not	0	-	-	-	-
**C-nonCTE #13***	E60s	F	+	-	-	-	Low	1	-	-	-	-
**C-nonCTE #14**	M50s	M	-	-	-	-	Not	0	-	-	-	-
**C-nonCTE #15**	E60s	M	-	-	-	-	Not	0	-	-	-	-
**C-nonCTE #16**	E50s	F	-	-	-	-	Low	1	+	-	-	-
**C-nonCTE #17**	L40s	M	-	-	-	-	Not	0	-	+	-	-
**C-nonCTE #18***	E50s	M	+	+	-	-	Not	0	+	-	-	-
**C-nonCTE #19***	L40s	M	+	-	-	-	Not	0	-	-	-	-
**C-nonCTE #20**	L40s	M	-	-	-	-	Not	0	-	-	-	-
**C-nonCTE #21***	M60s	M	+	-	-	-	Not	0	-	+	-	-
**C-nonCTE #22***	M40s	M	+	-	-	-	Not	0	-	-	-	-
**C-nonCTE #23**	E50s	M	-	-	-	-	Not	0	-	-	-	-
**C-nonCTE #24***	M60s	M	+	+	+, -	-	Low	1	+	+	-	-
**C-nonCTE #25**	E60s	M	-	-	-	-	Not	0	-	-	-	-
**C-nonCTE #26***	L60s	F	+	+	-	-	Low	1	-	-	-	-
**C-nonCTE #27**	M60s	M	-	-	-	-	Not	0	-	-	-	-
**C-nonCTE #28**	E50s	M	-	-	-	-	Not	0	-	-	-	-
**C-nonCTE #29***	M60s	M	+	-	-	-	Not	0	-	-	-	-
**C-nonCTE #30***	L50s	M	+	-	-	-	Low	1	-	-	-	-

© 2026 Tanaka H et al. *JAMA Neurology*.

Mean	56.23	M:F<br>25 : 5	Positive / available # of cases (%)	11/30<br>(37)	5/30<br>(17)	1/30 (1, 0)<br>(3)	0/30<br>(0)	High-Int: 0/30<br>(0)	8/30<br>(27)	4/30<br>(13)	4/30<br>(13)	3/30<br>(10)	0/30<br>(0)

Abbreviations: Age, age at death; E, early; M, mid; L, late (e.g., E70s = early 70s [70-73], M70s = mid-70s [74-76], L70s = late 70s [77-79]); F, female; M, male; Tau, severity of total tau pathology; Tau-N, neuronal tau pathology; Tau-Ast, astrocytic tau pathology; GM, gray matter; WM, white matter; TDP-43, TAR DNA-binding protein 43; AD-NC, Alzheimer's disease neuropathologic change[4]; CAA, cerebral amyloid angiopathy [5]; Aβ(A), A score of AD-NC[4]; AGD, argyrophilic grain disease[6]; LBD, Lewy body disease [7,9]; LATE, limbic predominant age-related TDP-43 encephalopathy[8]; Int, intermediate.

*, Spinal cord p-tau positive cases.

Semiquantitative assessment of the spinal cord pathology: (Tau: total, Tau-N, Tau-Ast) - = absent, + = minimal, ++ = mild, +++ = moderate, ++++ = severe; (TDP-43) - = absent, + = present, ++ = present with neuronal cytoplasmic inclusions.

© 2026 Tanaka H et al. *JAMA Neurology*.

eTable 11. Tau Pathology of Spinal Cord at Each Level in p-Tau-Positive 11 Non-CTE-NC Cases From Indeterminate RHI Group

Tau-positive cases	Tau[a]	level	Total	N	A-GM	A-WM
**C-nonCTE #3**	+	C	1	1	0	0
		T	0	0	0	0
		L	0	0	0	0
**C-nonCTE #7**	+	C	1*	0	0	0
		T	1	1	0	0
		L	1*	0	0	0
**C-nonCTE #13**	+	C	1*	0	0	0
		T	0	0	0	0
		L	0	0	0	0
**C-nonCTE #18**	+	C	1	1	0	0
		T	1*	0	0	0
		L	0	0	0	0
**C-nonCTE #19**	+	C	1*	0	0	0
		T	0	0	0	0
		L	0	0	0	0
**C-nonCTE #21**	+	C	1*	0	0	0
		T	0	0	0	0
		L	0	0	0	0
**C-nonCTE #22**	+	C	1*	0	0	0
		T	0	0	0	0
		L	0	0	0	0
**C-nonCTE #24**	+	C	1*	0	0	0
		T	1	1	0	0
		L	1	0	1	0
**C-nonCTE #26**	+	C	1	1	0	0
		T	1*	0	0	0
		L	1*	0	0	0
**C-nonCTE #29**	+	C	1*	0	0	0
		T	0	0	0	0
		L	0	0	0	0
**C-nonCTE #30**	+	C	1*	0	0	0
		T	0	0	0	0
		L	0	0	0	0
		Mean	0.20	0.06	0.01	0.00
	Frequency (%)[b]		11/30 (37)	5/30 (17)	1/30 (3)	0/30 (0)
					1/30 (3)[c]	

Abbreviations: Tau, tau severity score for individual cases; Total, severity of total tau pathology; N, neuronal tau pathology; A-GM, astrocytic tau pathology in the gray matter; A-WM, astrocytic tau pathology in the white matter; C, cervical; T, thoracic; L, lumbar.

Semiquantitative assessment of the spinal cord pathology: (Total-tau) 0 = absent, 1 = minimal, 2 = mild, 3 = moderate, 4 = severe; (Tau-N, A-GM, A-WM) 0 = absent, 1 = minimal, 2 = mild, 3 = moderate, 4 = severe, 5 = extremely severe.

[a] Tau severity score for each case was determined based on the highest score of "Total tau" among the spinal cord levels. - = absent, + = minimal, ++ = mild, +++ = moderate, ++++ = severe.

[b] Frequencies were determined as the ratio of positive cases to the number of available cases (n=30).

[c] Frequency of cases exhibiting all types of tau-positive astrocytes.

*, only tau-positive neurites/threads are present.

© 2026 Tanaka H et al. *JAMA Neurology*.

eTable 12. Summary of Clinicopathological Characteristics in Indeterminate RHI Group

	Indeterminate RHI (n=34)		
	**CTE (n=4)**	**nonCTE (n=30)**	***P*** [a]
**Clinical**			
**Age (mean, min-max, y)**	60.75 (56-67)	56.23 (41-67)	0.2584
**Sex (M:F)**	4:0	25:5	>0.9999
**Spinal cord**			
**Tau - Total (frequency, %)** [b]	4/4 (100)	11/30 (37)	**0.0294**
**(severity, mean)** [c]	1.08	0.20	**<0.0001**
**- Neuron (frequency, %)**	4/4 (100)	5/30 (17)	**0.0027**
**(severity, mean)**	0.33	0.06	**0.0104**
**- Astrocyte**	3/4 (75)	1/30 (3)	**0.0026**
**- in GM**	3/4 (75)	1/30 (3)	**0.0026**
**- in WM**	2/4 (50)	0/30 (0)	**0.0107**
**TDP-43**	1/4 (25)	0/30 (0)	0.1176
**α-Syn**	0/4 (0)	na	-
**Aβ**	4/4 (100)	na	-
**SAA**	0/4 (0)	na	-
**Brain (Mixed pathology)**			
**AD-NC (High-Int., %)**	0/4 (0)	0/30 (0)	>0.9999
**(severity, mean)** [d]	0.25	0.27	>0.9999
**Aβ(A) (frequency, %)**	1/4 (25)	8/30 (27)	>0.9999
**(severity, mean)** [e]	0.25	0.27	>0.9999
**CAA**	0/4 (0)	4/30 (13)	>0.9999
**AGD**	0/4 (0)	4/30 (13)	>0.9999
**LBD**	0/4 (0)	3/30 (10)	>0.9999
**LATE**	0/4 (0)	0/30 (0)	>0.9999

Abbreviations: CTE, cases with chronic traumatic encephalopathy neuropathologic change in the brain; *P*, *P* value; Age, age at death; y, years; M, male; F, female; RHI, repetitive head impacts; GM, gray matter; WM, white matter; TDP-43, TAR DNA-binding protein 43; α-Syn, α-synuclein; Aβ, amyloid-β; SAA, spinal amyloid angiopathy: amyloid angiopathy in the spinal cord vessels; AD-NC, Alzheimer's disease neuropathologic change[4]; CAA, cerebral amyloid angiopathy [5]; Aβ (A), A score of AD-NC[4]; AGD, argyrophilic grain disease[6]; LBD, Lewy body disease [7,9]; LATE, limbic predominant age-related TDP-43 encephalopathy[8]; Int, intermediate; na, not available.

[a] Items with statistically significant differences ($p < 0.05$) are indicated in bold.

[b] Frequencies were determined as the ratio of positive cases to the number of available cases.

[c] Severity was determined by averaging scores from all available cases across all spinal cord levels.

[d] AD-NC severity was determined by averaging the AD-NC scores, which were defined as follows: Not = 0; Low = 1; Intermediate = 2; High = 3.

[e] Aβ severity was calculated as the average of A scores of AD-NC[4].

© 2026 Tanaka H et al. *JAMA Neurology*.

eTable 13. Summary of Major Clinicopathological Findings in CTE-RHI Cases Aged <65

Clinical features						Spinal cord							Brain (mixed pathology)						
**Case #**	**Age**	**Sex**	**Duration, y**	**# of Concussions**		**Tau**	**N**	**A**	**TDP-43**	**α-Syn**	**Aβ**	**SAA**	**AD -NC**	**Aβ (A)**	**CAA**	**AGD**	**LBD**	**LATE**	**CTE level**
M-CTE #1	M40s	M	11	10		+	-	-	-	-	++	-	Low	1	-	-	-	-	Low
M-CTE #2	E50s	M	23	>10		+	-	-	-	-	++	-	Low	1	-	1	-	-	Low
Mean	47	M:F	17		Positive / available # of cases	2/2	0/2	0/2	0/2	0/2	2/2	0/2	High-Int: 0/2	2/2	0/2	1/2	0/2	0/2	Low: 2
		2:0			%	100	0	0	0	0	100	0	0	100	0	50	0	0	High: 0

Abbreviations: Age, age at death; E, early; M, mid; L, late (e.g., E70s = early 70s [70-73], M70s = mid-70s [74-76], L70s = late 70s [77-79]); M, male; F, female; Duration, duration of the exposure (contact sports, years); # of Concussions, documented number of concussions; Tau, severity of total tau pathology; N, neuronal tau pathology; A, astrocytic tau pathology; TDP-43, TAR DNA-binding protein 43; α-Syn, α-synuclein; Aβ, amyloid-β; SAA, spinal amyloid angiopathy: amyloid angiopathy in the spinal cord vessels; AD-NC, Alzheimer's disease neuropathologic change[4]; CAA, cerebral amyloid angiopathy [5]; Aβ (A), A score of AD-NC[4]; AGD, argyrophilic grain disease[6]; LBD, Lewy body disease [7,9]; LATE, limbic predominant age-related TDP-43 encephalopathy[8]; CTE-level, the level of severity of chronic traumatic encephalopathy neuropathologic change [2]; Int, intermediate.

Semiquantitative assessment of the spinal cord pathology: (Tau: total, Aβ) - = absent, + = minimal, ++ = mild, +++ = moderate, ++++ = severe; (TDP-43) - = absent, + = present, ++ = present with neuronal cytoplasmic inclusions; (α-Syn) - = absent, + = minimal/mild, ++ = moderate, +++ = severe; (Tau-N, Tau-A) - = absent, + = present.

© 2026 Tanaka H et al. *JAMA Neurology*.

eTable 14. Severity of Spinal Cord Pathology at Each Level in CTE-RHI Cases Aged <65

		Tau				Other misfolded proteins			Degeneration	
**Case #**	**level**	**Total**	**N**	**A-GM**	**A-WM**	**TDP-43**	**α-Syn**	**Aβ**	**APP**	**HLA-DR**
**M-CTE #1**	C	1*	0	0	0	0	0	2	1	5
	T	0	0	0	0	0	0	1	1	4
	L	0	0	0	0	0	0	1	1	4
**M-CTE #2**	C	0	0	0	0	0	0	2	1	4
	T	1*	0	0	0	0	0	1	1	4
	L	1*	0	0	0	0	0	1	1	3
	Mean	0.50	0.00	0.00	0.00	0.00	0.00	1.33	1.00	4.00
Positive/available # of cases (%)		2/2	0/2	0/2	0/2	0/2	0/2	2/2	2/2	2/2
		(100)	(0)	(0)	(0)	(0)	(0)	(100)	(100)	(100)

Abbreviations: Total, severity of total tau pathology; N, neuronal tau pathology; A-GM, astrocytic tau pathology in the gray matter; A-WM, astrocytic tau pathology in the white matter; TDP-43, TAR DNA-binding protein 43; α-Syn, α-synuclein; Aβ, amyloid-β; APP, APP-positive axonal spheroids; HLA-DR, HLA-DR-positive microglia; C, cervical; T, thoracic; L, lumbar.
Semiquantitative assessment of the spinal cord pathology: (Tau-Total, Aβ) 0 = absent, 1 = minimal, 2 = mild, 3 = moderate, 4 = severe; (TDP-43) 0 = absent, 1 = present, 2 = present with neuronal cytoplasmic inclusions; (α-Syn, APP) 0 = absent, 1 = minimal/mild, 2 = moderate, 3 = severe; (Tau-N, A-GM, A-WM, HLA-DR) 0 = absent, 1 = minimal, 2 = mild, 3 = moderate, 4 = severe, 5 = extremely severe. *, only tau-positive neurites/threads are present.

© 2026 Tanaka H et al. *JAMA Neurology*.

eTable 15. Summary of Major Clinicopathological Findings in nonCTE-RHI Cases

Clinical features					Spinal cord pathology							Brain (mixed pathology)						
**Case #**	**Age**	**Sex**	**Duration (y)**	**Conc**	**Tau**	**N**	**Tau-Ast (GM, WM)**	**TDP-43**	**α-Syn**	**Aβ**	**SAA**	**ADNC**	**Aβ (A)**	**CAA**	**AGD**	**LBD**	**LATE**	**Other**
nonCTE-RHI #1	E70s	M	32	> 4	+	+	(-)	-	-	-	-	High	3	Type 2	-	Amyg	-	
nonCTE-RHI #2	E70s	M	14	>10	+++	++	(+,-)	-	-	-	-	Not	-	-	3	-	-	PSP
nonCTE-RHI #3	E80s	M	40	8	+	+	(-)	++	-	+	Type 2	Int	2	Type 1	-	Amyg	2	
nonCTE-RHI #4	M70s	M	8	4	+	+	(+,-)	-	-	-	-	Not	-	-	1	-	2	
nonCTE-RHI #5	E20s	M	na	4	-	-	(-)	-	-	-	-	Not	-	-	-	-	-	
nonCTE-RHI #6	L40s	M	17.5	9	-	-	(-)	-	-	-	-	Low	1	-	-	-	-	Subarachnoid hemorrhage
nonCTE-RHI #7	E30s	F	10	12	-	-	(-)	-	-	+	-	Not	-	-	-	-	-	Cortical microhemorrhages, NIID
Mean or frequency (%)	57.29	M:F 6:1	20.25		4/7 (57)	4/7 (57)	2/7 (2,0) (29)	1/7 (14)	0/7 (0)	2/7 (29)	1/7 (14)	High-Int: 2/7 (29)	3/7 (43)	2/7 (29)	2/7 (29)	2/7 (29)	2/7 (29)	

Abbreviations: Age, age at death; E, early; M, mid; L, late (e.g., E70s = early 70s [70-73], M70s = mid-70s [74-76], L70s = late 70s [77-79]); M, male; F, female; Duration, duration of the exposure (contact sports, years); Conc, documented number of concussions; Tau, severity of total tau pathology; N, neuronal tau pathology; A, astrocytic tau pathology; TDP-43, TAR DNA-binding protein 43; α-Syn, α-synuclein; Aβ, amyloid-β; SAA, spinal amyloid angiopathy: amyloid angiopathy in the spinal cord vessels; AD-NC, Alzheimer's disease neuropathologic change[4]; CAA, cerebral amyloid angiopathy [5]; Aβ (A), A score of AD-NC[4]; AGD, argyrophilic grain disease[6]; LBD, Lewy body disease [7,9]; LATE, limbic predominant age-related TDP-43 encephalopathy[8]; CTE-level, the level of severity of chronic traumatic encephalopathy neuropathologic change [2]; Int, intermediate. Semiquantitative assessment of the spinal cord pathology: (Tau: total, Aβ) - = absent, + = minimal, ++ = mild, +++ = moderate, ++++ = severe; (TDP-43) - = absent, + = present, ++ = present with neuronal cytoplasmic inclusions; (α-Syn) - = absent, + = minimal/mild, ++ = moderate, +++ = severe; (Tau-N) 0 = absent, 1 = minimal, 2 = mild, 3 = moderate, 4 = severe, 5 = extremely severe. (Tau-Ast) - = absent, + = present.

© 2026 Tanaka H et al. *JAMA Neurology*.

eTable 16. Severity of Spinal Cord Pathology at Each Level in nonCTE-RHI Cases

	Tau [a]	level	Total	N	A-GM	A-WM
**nonCTE-RHI #1**	+	C	1	1	0	0
		T	0	0	0	0
		L	1	0	0	0
**nonCTE-RHI #2**	+++	C	3	2	2	0
		T	1	1	0	0
		L	1	1	0	0
**nonCTE-RHI #3**	+	C	1	0	0	0
		T	0	0	0	0
		L	1	1	0	0
**nonCTE-RHI #4**	+	C	1	1	1	0
		T	1	1	0	0
		L	1	1	0	0
**nonCTE-RHI #5**	-	C	0	0	0	0
		T	0	0	0	0
		L	0	0	0	0
**nonCTE-RHI #6**	-	C	0	0	0	0
		T	0	0	0	0
		L	0	0	0	0
**nonCTE-RHI #7**	-	C	0	0	0	0
		T	0	0	0	0
		L	0	0	0	0
**Summary of all 7 cases**		Mean	0.57	0.43	0.14	0.00
	Positive / available # of cases (%)		4/7 (57)	4/7 (57)	2/7 (29)	0/7 (0)

Abbreviations: Tau, tau severity score for individual cases; Total, severity of total tau pathology; N, neuronal tau pathology; A-GM, astrocytic tau pathology in the gray matter; A-WM, astrocytic tau pathology in the white matter; C, cervical; T, thoracic; L, lumbar; S, sacral. Semiquantitative assessment of the spinal cord pathology: (Tau-Total) 0 = absent, 1 = minimal, 2 = mild, 3 = moderate, 4 = severe; (Tau-N, A-GM, A-WM) 0 = absent, 1 = minimal, 2 = mild, 3 = moderate, 4 = severe, 5 = extremely severe.

[a] Tau severity score for each case was determined based on the highest score of "Total tau" among the spinal cord levels. - = absent, + = minimal, ++ = mild, +++ = moderate, ++++ = severe.

© 2026 Tanaka H et al. *JAMA Neurology*.

eTable 17. Correlation Between Spinal Cord and Brain Pathologies in CTE-RHI Cases Aged ≥65

	Spinal cord																	
	Tau		N		A-GM		A-WM		TDP-43		α-Syn		Aβ		HLA-DR		APP	
**Brain**	r	p	r	p	r	p	r	p	r	p	r	p	r	p	r	p	r	p
**AD-NC**	0.269	0.350	0.240	0.409	0.166	0.567	**0.545**	**0.049**	0.306	0.300	-0.341	0.219	0.105	0.712	0.159	0.593	0.029	0.932
**Aβ (A)**	0.380	0.211	0.298	0.345	0.277	0.351	0.536	0.053	0.255	0.502	-0.261	0.423	0.119	0.717	0.499	0.095	0.118	0.653
**CAA**	0.282	0.377	0.274	0.433	0.194	0.509	0.442	0.125	-0.178	0.609	0.000	1.000	-0.334	0.277	0.407	0.164	0.000	1.000
**AGD**	-0.224	0.441	0.279	0.393	0.086	0.766	-0.365	0.204	-0.161	0.649	-0.048	0.874	**0.607**	**0.032**	-0.008	0.988	0.006	0.928
**LBD**	-0.109	0.734	0.103	0.745	-0.377	0.202	-0.199	0.510	-0.551	0.066	**0.886**	**0.00031**	-0.230	0.461	0.145	0.632	-0.451	0.128
**LATE**	0.413	0.143	0.157	0.604	0.282	0.329	**0.600**	**0.029**	-0.176	0.555	-0.018	0.974	-0.531	0.053	0.277	0.336	-0.248	0.389
**CTE-level**	0.176	0.658	0.219	0.543	0.151	0.630	0.530	0.084	0.281	0.426	-0.351	0.271	-0.116	0.821	-0.157	0.594	0.045	0.953

Abbreviations: Tau, total tau pathology; N, neuronal tau pathology; A-GM, astrocytic tau pathology in the gray matter; A-WM, astrocytic tau pathology in the white matter; TDP-43, TAR DNA-binding protein 43; α-Syn, α-synuclein; Aβ, amyloid-β; APP, APP-positive axonal spheroids; HLA-DR, HLA-DR-positive microglia; AD-NC, Alzheimer's disease neuropathologic change[4]; Aβ (A), A score of AD-NC[4]; CAA, cerebral amyloid angiopathy [5]; AGD, argyrophilic grain disease[6]; LBD, Lewy body disease [7,9]; LATE, limbic predominant age-related TDP-43 encephalopathy[8]; CTE-level, the level of severity of chronic traumatic encephalopathy neuropathologic change [2]; r, Spearman's correlation coefficient; p, p value.
Bold values indicate statistically significant correlations ($p < 0.05$, two-tailed Spearman's rank correlation).

© 2026 Tanaka H et al. *JAMA Neurology*.

eTable 18. Comparison of CTE-NC Cases With and Without Comorbid Primary Tauopathies

Spinal Tau pathology scores, mean	Total	N	A-GM	A-WM
**(i) CTE-NC without** AGD and other tauopathy [a] (n=14)	1.60	0.70	0.98	1.10
**(ii) CTE-NC with** AGD and other tauopathy [a] (n=6)	1.50	1.07	1.00	0.57
**(i) vs (ii), P value**	0.7125	0.1722	0.9954	0.1723
**(iii) Controls** (n=13)	0.74	0.35	0.00	0.00
**(i) vs (iii), P value**	**0.0003**	0.0571	**<0.0001**	**<0.0001**

Abbreviations: Tau, total tau pathology; N, neuronal tau pathology; A-GM, astrocytic tau pathology in the gray matter; A-WM, astrocytic tau pathology in the white matter; AGD, argyrophilic grain disease.
Bold values indicate statistically significant correlations ($p < 0.05$, Mann-Whitney U test).
[a] Other primary tauopathies include corticobasal degeneration (CBD) and globular glial tauopathy (GGT).

© 2026 Tanaka H et al. *JAMA Neurology*.

eTable 19. Clinical Features in All 23 Cases in Confirmed RHI Group

Group i) CTE-NC	ii) Age	Study #	Age at death	Sex	Type of exposure (Sports / Occupation)	Details (e.g., position in American football)	Duration, y	# of Concussions (& semiquantitative score)		Cause of Concussions	Spinal Cord Injury (semiquantitative score, details)		Surgical History	Motor Symptoms (semiquantitative score, details)		(Motor Neuron Signs)		Constitutional Symptoms	Neurological exam to indicate clinical spinal cord involvement (semiquantitative score, details)	
**CTE-NC**	**≥65 years**	**CTE #1**	E80s	M	Professional hockey player	Right Wing	18	Multiple	+++++	Hockey related	-		-	+	2 years- slow gait, impaired balance, Multiple falls, Left hand tremor	-		None	-	No findings on exam in a year
		**CTE #2**	E70s	M	North American professional football player	Running Back, Full back of half back man, returner, kick offs - tackling team	10	16	+++	Sport related	-		Knee surgery	+-	7 years- trouble using tools/utensils hands tremor, balance impairment	-		Muscle and joints aches Urinary and bowel incontinence	-	No findings on a single exam
		**CTE #3**	L70s	M	North American professional football player	Line-backer	12	> 20	++++	Sport related- hitting heads against the opponents	+-	Low back pain and numbness in the lower extremities	-	+	>10 years- trouble with balance and gait	-		Fatigue Muscle aches	na	na
		**CTE #4**	L70s	M	North American professional football player	Tight end and wide receiver	13	6	++	Sport related- blows to the head	-		Cardiac surgery, surgery for broken nose	-	None	-		Muscle aches Mild neck pain	-	No findings on a single exam
		**CTE #5**	M80s	M	North American professional football player and Wrestler	Football-offensive and defensive tackle, middle guard and end; Wrestling-high impact wrestler	26 (Football: 14, Wrestling: 17)	Multiple	+++++	sport related- he was playing football and wrestling and hit his head every day	+-	Low back pain	Knee replacement	+	5 years- trouble with balance and gait, Multiple falls, trouble using tools/utensils, hand tremor Fasciculation, atrophy	+	Fasciculation, atrophy	Fatigue Muscle aches Urinary incontinence RBD	+	Bilateral thenar and first dorsal interosseous (FDI) muscle atrophy with fasciculation; Romberg positive, could not tandem gait
		**CTE #6**	M80s	M	North American professional football player	Linebacker	3 (professional)	3	+	Bike accident, sport related, other	+	Severe neck injury during a	Prostate surgery	+	1-2 years -Trouble with balance and gait	-		Fatigue	na	na

© 2026 Tanaka H et al. *JAMA Neurology*.

										football game			In last 2 years of life					
**CTE #7**	M80 s	M	North American profession al football player	Offensive Guard	13	10	+++	Related to falls	+	Spinal stenosis in the cervical region.	Both knee surgery	+	10 years, mild weakness in lower extremities , falls, -5 years left hand decreased dexterity	+	weakness in lower extremities	Fatigue, Light-headed, vertigo	-	No findings on a single exam
**CTE #8**	E80 s	M	North American profession al football player	Running Back, Defensive back position	8	5	++	Sport related- he had tackled and fell on the ground. He got a blow to the head by football.	+	Spinal stenosis	Knee surgery	+	developed some difficulty walking and used a cane. - 6 months wheelchair bound.	-		Headache after one concussion that resolved	+	Bilateral sensory loss suggests posterior column and spinothalam ic tract dysfunction. Affecting posterior columns (responsible for vibration, propriocepti on, and fine touch). Spinothalam ic tract involvement (pain and temperature) is also likely given absent pinprick and temperature sensation.
**CTE #9**	M80 s	M	Non-profession al hockey player	Center-Ice	20	7	++	Fall, hit head with the objects	+	Spinal cord surgery	Discectomy in lumbosacral area. They removed a piece pressing on the sciatic nerve. Has had back issues/pain ever since. Hip surgery	+	3 years of difficulty walking, fall	-		None	+	Motor: Upper motor neuron pattern of weakness in lower limbs. Brisk lower limb reflexes. Sensory: Loss of dorsal column modalities (propriocepti on, light touch) may involve posterior

© 2026 Tanaka H et al. *JAMA Neurology*.

																		spinal cord. Coordination deficits and balance impairment.
**CTE #10**	M80s	M	Non-professional rugby player	Centre back and wing back	19	6	++	Sport related- hit head while playing	+	Spinal cord surgery	Decompression spinal cord surgery (L3-5, C6-7), both knee replacement	+	Impaired balance and gait, repeated falls last 5 years, muscle weakness and rigidity, trouble using utensils, handwriting changed, myoclonic jerks, trouble swallowing	+	muscle weakness	Nausea and light-headedness, Joint pain for 10 years, urinary and bowel incontinence for the last 3-6 months	na	na
**CTE #11**	M80s	M	North American professional football player	Full Back/ Half-Back, and Running Back	11	3	+	Sport related- hit his head numerous time in his sports career	-		Bowel resection, bilateral rotator cuff injury repair	na	Left-sided stiffness, moderate bradykinesia, a slow shuffling gait, dragging of the left leg for 4 years after a stroke	na		Moderate bowel and urinary incontinence in the last 3 months	-	No findings on a single exam
**CTE #12**	L70s	M	Professional hockey player	Rt. Winger	28	10	+++	Sport related	-		Knee and shoulder surgery	+	1-2 years- Impaired balance and gait, Mild tremor, bradykinesia, handwriting changed (messier)	-		Moderate fatigue and lightheadedness for the last 2 years. Bowel and urinary continence for the last 3-6 months.	na	na

© 2026 Tanaka H et al. *JAMA Neurology*.

		**CTE #13**	L60s	M	Non-professional hockey player	Defence	30	10	+++	Mainly sport related- hit the opponent with head	-		Right knee replacement	+	Impaired balance and gait, repeated falls last 5 years, bradykinesia, muscle weakness and rigidity, trouble using utensils, handwriting changed, Involuntary movements, myoclonic jerks, trouble swallowing	+	muscle weakness	Fatigue, joint pain for >10 years, urinary incontinence for 5 years, bowel incontinence for a year.	na	na
		**CTE #14**	L70s	M	Non-professional rugby union player	Position unknown	32	> 2	+	Related to rugby	-		Cardiac surgery	-	None	-		None	na	na
**CTE-NC**	**<65 years**	**M-CTE #1**	M40s	M	Professional hockey player	Rt. Winger	11	10	+++	Related to the sports	-		Ankle surgery, surgery for broken arm	-	None	-		Headache and fatigue for > 10 years	na	na
		**M-CTE #2**	E50s	M	Professional hockey player	Forward	23	>10	+++	Sport related- hit his head numerous time in his sports career	+-	Low back pain	Shoulder surgery	+-	Mild tremor and bradykinesia for 2 years	-		Headache, fatigue, nausea, and light-headedness, muscle and joint pain for >10 years	na	na
**Non-CTE-NC**	**≥65 years**	**nonCTE-RHI #1**	E70s	M	Military	Navy	32	> 4	++	Related to water landings in parachute support role, falling off a horse, hit by a car while cycling	-		Hernia repairs	-	None	-		None	na	na

© 2026 Tanaka H et al. *JAMA Neurology*.

	**nonCTE-RHI #2**	E70s	M	professional hockey player	Defence	14	>10	+++	Sport related- hit his head and smashed face, fall.	+	Spinal cord surgery	Shoulder and jaw surgery; laminectomy	+	Gait difficulty; impaired balance and recurrent falls; mild to moderate muscle weakness; Intention tremor; Muscle fasciculation; Myoclonic jerk; Handwriting had changed; Dysphagia	+	Fasciculation, muscle weakness	Fatigue for>10 y; Muscle ache> 10 y; Joint ache 10 y; Urinary incontience- 5 y	+	Decreased sesnation L4-5
	**nonCTE-RHI #3**	E80s	M	Non-professional hockey player	Forward/ winger	40	8	++	Hit with oppnent players, got run over and wound up on the ground.	-		Surgery for broken ankle	+	Gait difficulty; impaired balance; falls; mild to moderate muscle weakness; rigidity; dysphagia; trouble using utensils; handwriting had changed	+	muscle weakness	Moderate to severe fatigue; Bowel and bladder incontinence for a year	-	No findings on a single exam
	**nonCTE-RHI #4**	M70s	M	North American professional football player	Center/tackle/linebacker	8	4	++	Took blow in his head with the ball, hit with other player's helmet, got run over and wound up on the ground.	+	Spinal stenosis (surgery)	Spinal cord surgery	+	Gait difficulty; impaired balance; repeated falls; bradykinesia; muscle weakness, handwriting changed	+	muscle weakness	Muscle ache and joint aches for 10 y; light-headedness for 5 years, and severe fatigue in a year	-	No findings on a single exam
**<65 years**	**nonCTE-RHI #5**	E20s	M	Non-professional hockey player	na	na	4	++	Bodychecking (ice hockey)	-		-	-	None	-		None	na	na

© 2026 Tanaka H et al. *JAMA Neurology*.

**nonCTE-RHI #6**	L40s	M	Semi-professional hockey player and wrestler	Forward	17.5 (Hockey: 16, wrestling: 1.5)	9	++	Sport related- hit his head numerous time in his sports career	-		Tonsillectomy	-	None	-	None	na	na
**nonCTE-RHI #7**	E30s	F	Non-professional hockey player	Defence	10	12	+++	Mainly sport related- hit the opponent with head	+-	Moderate back pain	-	+-	Recurrent falls; Dyspraxia; Muscle twitching in her hand and severe body twitching while sleeping; Moderate tremor	-	Migraine for >10 y; Fatigue for 10 y; Moderate urinary incontinence for 2 y	-	No findings on a single exam

Abbreviations: Age, age at death; E, early; M, mid; L, late (e.g., E70s = early 70s [70-73], M70s = mid-70s [74-76], L70s = late 70s [77-79]); M, male; F, female; Duration, duration of the exposure (contact sports, years); Concussion, documented number of concussions. For the clinical findings of number of concussions, spinal cord injury, and motor symptoms, semi-quantitative scores were applied as follows: Number of concussions was categorized based on available descriptions. Since the most affected cases were described only as “multiple” without an exact count, we defined: (+) = 1–3 episodes; (++) = 4–9; (+++) = 10–20; (++++) >20; (+++++) = multiple (exact number unknown). Spinal cord injury was scored as (+-) when symptoms possibly related to injury could not be ruled out, and (+) when a history of definitive spinal cord/spinal column injury or surgery was present. Regarding motor symptoms, we scored cases as “(+-)” when any motor symptoms, including involuntary movements, were present. Cases were scored as “(+)” when motor symptoms more suggestive of spinal cord involvement—such as lower motor neuron (LMN) signs or gait disturbances—were observed.

© 2026 Tanaka H et al. *JAMA Neurology*.

eTable 20. Clinicopathological Correlation in CTE-RHI Cases Aged ≥65

		Clinical features									
		Age at death		Duration of exposure		# of Concussion		Spinal cord injury		Motor symptoms	
		r	p	r	p	r	p	r	p	r	p
Spinal cord pathology	Tau	0.438	0.117	0.086	0.768	-0.014	0.950	0.245	0.397	**0.608**	**0.049**
	-N	0.467	0.099	-0.134	0.656	-0.328	0.257	0.260	0.419	0.422	0.268
	-A-GM	**0.546**	**0.046**	-0.174	0.547	0.069	0.815	0.402	0.148	**0.671**	**0.021**
	-A-WM	0.233	0.418	0.137	0.637	0.001	0.997	0.178	0.532	0.310	0.279
	TDP-43	0.206	0.481	-0.435	0.124	-0.357	0.221	0.230	0.435	0.075	0.913
	α-Syn	0.184	0.527	0.103	0.723	0.040	0.891	-0.108	0.788	0.165	0.706
	Aβ	-0.278	0.331	-0.362	0.203	-0.073	0.806	0.253	0.400	0.138	0.625
	HLA-DR	**0.564**	**0.038**	0.084	0.774	-0.273	0.345	0.117	0.684	0.224	0.615
	APP	0.164	0.572	-0.462	0.098	-0.034	0.912	**0.687**	**0.012**	0.210	0.549
Brain pathology	AD-NC	0.105	0.720	-0.146	0.614	-0.355	0.213	-0.034	0.923	0.008	1.000
	Aβ (A)	0.359	0.212	-0.111	0.714	-0.386	0.167	0.212	0.431	0.123	0.664
	CAA	0.495	0.078	0.027	0.945	-0.376	0.199	0.104	0.846	-0.054	0.944
	AGD	-0.051	0.860	-0.141	0.626	-0.127	0.659	0.235	0.444	0.118	0.706
	LBD	0.053	0.867	0.324	0.278	0.036	0.910	-0.288	0.347	0.071	0.962
	LATE	0.288	0.314	0.188	0.515	-0.124	0.673	-0.104	0.743	0.014	1.000
	CTE-level	0.108	0.737	-0.212	0.501	-0.217	0.503	-0.069	1.000	-0.064	1.000

Abbreviations: Tau, total tau pathology; N, neuronal tau pathology; A-GM, astrocytic tau pathology in the gray matter; A-WM, astrocytic tau pathology in the white matter; TDP-43, TAR DNA-binding protein 43; α-Syn, α-synuclein; Aβ, amyloid-β; APP, APP-positive axonal spheroids; HLA-DR, HLA-DR-positive microglia; AD-NC, Alzheimer's disease neuropathologic change[4]; Aβ (A), A score of AD-NC[4]; CAA, cerebral amyloid angiopathy [5]; AGD, argyrophilic grain disease[6]; LBD, Lewy body disease [7,9]; LATE, limbic predominant age-related TDP-43 encephalopathy[8]; CTE-level, the level of severity of chronic traumatic encephalopathy neuropathologic change [2]; r, Spearman's correlation coefficient; p, p value.
Bold values indicate statistically significant correlations ($p < 0.05$, two-tailed Spearman's rank correlation).

© 2026 Tanaka H et al. *JAMA Neurology*.

eReferences

1. Alosco ML, Cherry JD, Huber BR, et al. Characterizing tau deposition in chronic traumatic encephalopathy (CTE): utility of the McKee CTE staging scheme. *Acta Neuropathologica*. 2020;140(4):495–512. doi:10.1007/s00401-020-02197-9
2. Bieniek KF, Cairns NJ, Crary JF, et al. The Second NINDS/NIBIB Consensus Meeting to Define Neuropathological Criteria for the Diagnosis of Chronic Traumatic Encephalopathy. *Journal of Neuropathology & Experimental Neurology*. 2021;80(3):210–219. doi:10.1093/jnen/nlab001
3. Danics K, Forrest SL, Kovacs GG. Chronic traumatic encephalopathy neuropathologic change in homeless. *Acta Neuropathologica*. 2025;149(1):28. doi:10.1007/s00401-025-02867-6
4. Montine TJ, Phelps CH, Beach TG, et al. National Institute on Aging-Alzheimer's Association guidelines for the neuropathologic assessment of Alzheimer's disease: a practical approach. *Acta Neuropathologica*. 2012;123(1):1–11. doi:10.1007/s00401-011-0910-3
5. Thal DR, Ghebremedhin E, Rüb U, Yamaguchi H, Del Tredici K, Braak H. Two Types of Sporadic Cerebral Amyloid Angiopathy. *Journal of Neuropathology & Experimental Neurology*. 2002;61(3):282–293. doi:10.1093/jnen/61.3.282
6. Saito Y, Ruberu NN, Sawabe M, et al. Staging of Argyrophilic Grains: An Age-Associated Tauopathy. *Journal of Neuropathology & Experimental Neurology*. 2004;63(9):911–918. doi:10.1093/jnen/63.9.911
7. Braak H, Del Tredici K, Rüb U, De Vos RA, Steur ENJ, Braak E. Staging of brain pathology related to sporadic Parkinson's disease. *Neurobiology of aging*. 2003;24(2):197–211.
8. Nelson PT, Dickson DW, Trojanowski JQ, et al. Limbic-predominant age-related TDP-43 encephalopathy (LATE): consensus working group report. *Brain*. 2019;142(6):1503–1527. doi:10.1093/brain/awz099
9. Attems J, Toledo JB, Walker L, et al. Neuropathological consensus criteria for the evaluation of Lewy pathology in post-mortem brains: a multi-centre study. *Acta Neuropathologica*. 2021;141(2):159–172. doi:10.1007/s00401-020-02255-2

© 2026 Tanaka H et al. *JAMA Neurology*.
