# Supplementary material for: Spinal Cord Tau and Protein Copathologies Associated With Chronic Traumatic Encephalopathy
Source: JAMA Neurol. 2026 Jan 26;83(3):231–41. doi: 10.1001/jamaneurol.2025.5421 (PMC12836280; doi:10.1001/jamaneurol.2025.5421)
Supplement: Supplement 2. — Data Sharing Statement [file jamaneurol-e255421-s002.pdf]

Data Sharing Statement

Tanaka. Spinal Cord Tau and Protein Copathologies Associated With Chronic Traumatic Encephalopathy. *JAMA Neurol*. Published January 26, 2026.
doi:10.1001/jamaneurol.2025.5421

Data
Data available: No

Additional Information
Explanation for why data not available: We provide all data in the online supplement file
